# SIRP**γ**-expressing cancer stem-like cells promote immune escape of lung cancer via Hippo signaling

**DOI:** 10.1172/JCI141797

**Published:** 2022-03-01

**Authors:** Chuan Xu, Guoxiang Jin, Hong Wu, Wei Cui, Yu-Hui Wang, Rajesh Kumar Manne, Guihua Wang, Weina Zhang, Xian Zhang, Fei Han, Zhen Cai, Bo-Syong Pan, Che-Chia Hsu, Yiqiang Liu, Anmei Zhang, Jie Long, Hongbo Zou, Shuang Wang, Xiaodan Ma, Jinling Duan, Bin Wang, Weihui Liu, Haitao Lan, Qing Xiong, Gang Xue, Zhongzhu Chen, Zhigang Xu, Mark E. Furth, Sarah Haigh Molina, Yong Lu, Dan Xie, Xiu-Wu Bian, Hui-Kuan Lin

**Affiliations:** 1 Department of Cancer Biology, Wake Forest School of Medicine, Winston-Salem, North Carolina, USA.; 2 Institute of Pathology and Southwest Cancer Center, Key Laboratory of Tumor Immunopathology of Ministry of Education of China, Southwest Hospital, Third Military Medical University, Chongqing, China.; 3 Integrative Cancer Center and Cancer Clinical Research Center, Sichuan Cancer Hospital & Research Institute, School of Medicine, University of Electronic Science and Technology of China, Chengdu, China.; 4 Guangdong Provincial People’s Hospital, Guangdong Academy of Medical Sciences, Guangzhou, China.; 5 School of Life Science and Biopharmaceutics, Shenyang Pharmaceutical University, Shenyang, China.; 6 Sun Yat-sen University Cancer Center, State Key Laboratory of Oncology in South China, Collaborative Innovation Center for Cancer Medicine, Guangzhou, China.; 7 Sichuan Academy of Medical Sciences, Sichuan Provincial People’s Hospital, University of Electronic Science and Technology of China, Chengdu, China.; 8 Immunotherapy Platform, University of Texas MD Anderson Cancer Center, Houston, Texas, USA.; 9 Department of Microbiology and Immunology, Wake Forest School of Medicine, Winston-Salem, North Carolina, USA.; 10 Chongqing Engineering Laboratory of Targeted and Innovative Therapeutics, Chongqing Key Laboratory of Kinase Modulators as Innovative Medicine, IATTI, Chongqing University of Arts and Sciences, Chongqing, China.; 11 Wake Forest Innovations, Wake Forest Baptist Medical Center, Winston-Salem, North Carolina, USA.

**Keywords:** Immunology, Cancer

## Abstract

Cancer stem-like cells (CSLCs) acquire enhanced immune checkpoint responses to evade immune cell killing and promote tumor progression. Here we showed that signal regulatory protein **γ** (SIRP**γ**) determined CSLC properties and immune evasiveness in a small population of lung adenocarcinoma (LUAD) cancer cells. A SIRP**γ**^hi^ population displayed CSLC properties and transmitted the immune escape signal through sustaining CD47 expression in both SIRP**γ**^hi^ and SIRP**γ**^lo/–^ tumor cells. SIRP**γ** bridged MST1 and PP2A to facilitate MST1 dephosphorylation, resulting in Hippo/YAP activation and leading to cytokine release by CSLCs, which stimulated CD47 expression in LUAD cells and consequently inhibited tumor cell phagocytosis. SIRP**γ** promoted tumor growth and metastasis in vivo through YAP signaling. Notably, SIRP**γ** targeting with genetic SIRP**γ** knockdown or a SIRP**γ**-neutralizing antibody inhibited CSLC phenotypes and elicited phagocytosis that suppressed tumor growth in vivo. *SIRPG* was upregulated in human LUAD and its overexpression predicted poor survival outcome. Thus, SIRP**γ**^hi^ cells serve as CSLCs and tumor immune checkpoint–initiating cells, propagating the immune escape signal to the entire cancer cell population. Our study identifies Hippo/YAP signaling as the first mechanism by which SIRP**γ** is engaged and reveals that targeting SIRP**γ** represents an immune- and CSLC-targeting strategy for lung cancer therapy.

## Graphical abstract

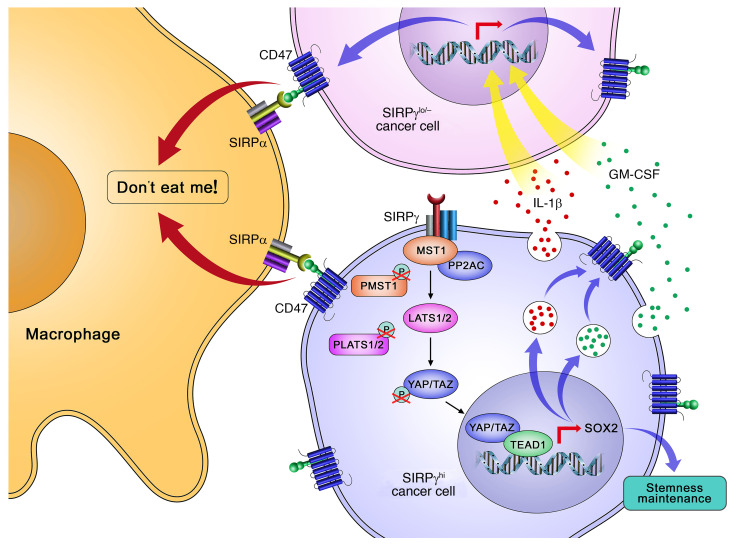

## Introduction

Non–small cell lung cancer (NSCLC) accounts for approximately 85% of all lung cancer. The 5-year survival rate is about 14% for stage IIIA NSCLC, while it is about 5% for stage IIIB. However, once NSCLC has reached stage IV and metastasized to different places, it is very difficult to treat. The 5-year survival rate for stage IV NSCLC is just about 6% (1, 2). Anti-EGFR and anti-ALK targeted therapies are the frontline treatments for advanced NSCLC with EGFR mutations and ALK mutations, respectively, while platinum-based chemotherapy is the first line of treatment for advanced NSCLC without targetable mutations (1, 2). Interestingly, recent studies suggest that anti–PD-1/PD-L1 immunotherapy is a new and effective strategy for advanced NSCLC with noticeable expression of PD-L1 (2). While NSCLC patients initially show great benefit from these treatments, the response is only transient, with relatively short duration, likely due to acquired resistant mechanisms and/or changes in microenvironments within cancer and immune cells leading to treatment failures (2). Once this scenario occurs, there is no other promising way to deal with these recurring NSCLCs, which will cause mobility and mortality in NSCLC patients. Identification of effective therapeutic strategies is therefore an urgent need for advanced NSCLC. While the resistance mechanisms underlying recurring NSCLC after therapy are not well understood, it has been proposed that a unique cell population with cancer stem-like cell (CSLC) properties that either preexists before the therapy or occurs during the treatment is responsible for NSCLC aggressiveness, metastasis, and the resistance to current treatments. Identification of a unique CSLC population in NSCLC and its regulatory mechanisms is of significance to develop an effective strategy for advanced NSCLC and/or for overcoming the resistance to current standard of care.

Cancer cells acquire immune inhibitory checkpoints to escape from killing by immune cells, such as T cells and macrophages (3–5). CSLCs might acquire enhanced immune checkpoint responses to evade the immune killing and promote tumor progression (6–8). An important gap in our knowledge concerns whether and how CSLCs may propagate signals for immune evasion to the bulk of non-CSLC tumor cells. Identifying this small cell population with CSLC properties and the underlying mechanism is instrumental for developing a new strategy for targeting cancer by attacking both CSLC and immune escape properties, thus representing a unique CSLC and immune targeting strategy.

The signal regulatory proteins (SIRPs) primarily associated with the modulation of immune functions are involved in the negative regulation of receptor tyrosine kinase–coupled signaling processes. SIRPα (SHPS1, CD172a), the most intensively studied family member, is expressed mainly on myeloid lineages, including macrophages and dendritic cells. It interacts with the cell surface protein CD47 to mediate “don’t eat me” signaling, thereby abrogating phagocytosis of various CD47-positive cells by macrophages (9, 10). Expression of CD47 in cancer cells contributes to immune evasion and subsequent tumor progression (3). While SIRPα is also expressed by certain cancer cells, it appears to inhibit their proliferation independently of its role in modulating antitumor immunity (11, 12). SIRPγ, also known as SIRPβ2 and CD172g, is a transmembrane glycoprotein with extracellular immunoglobulin-like domains. It belongs to the SIRP family of paired receptors, encoded by a set of genes mapping closely together on human chromosome 20p13, which also includes SIRPα, SIRPβ (SIRP-β1), and soluble SIRPδ (9, 10). SIRPγ is expressed preferentially in T lymphocytes and activated natural killer (NK) cells (13). However, the functional roles of SIRPγ and its signaling mechanisms are less well understood. To the best of our knowledge, previous studies have not evaluated the expression of SIRPγ in solid tumor cells, nor implicated the protein in the regulation of any cancer phenotypes.

A small population with CSLC properties has been identified in leukemias and solid tumors (14–18). CSLCs, which are capable of self-renewal and differentiation, are thought to play a pivotal role in tumor initiation, progression, and metastasis, and often contribute to resistance to therapy and cancer relapse (19–22). Specifically targeting CSLCs therefore offers a promising strategy to augment current cancer therapies. CSLCs express characteristic cell surface markers (e.g., CD44 and CD133), which potentially could serve as targets for cancer therapy. However, implementation of this approach has been limited to date either by side effects due to important roles played by the target proteins in a variety of normal tissues and/or by modest efficacy in eliminating CSLCs (23). We therefore sought to identify a targetable CSLC surface marker that plays a critical role in maintaining cancer stemness and transmitting immune escape signals to the bulk cancer cells.

## Results

### SIRPγ serves as a CSLC marker and promotes tumor growth.

**Figure 1:**
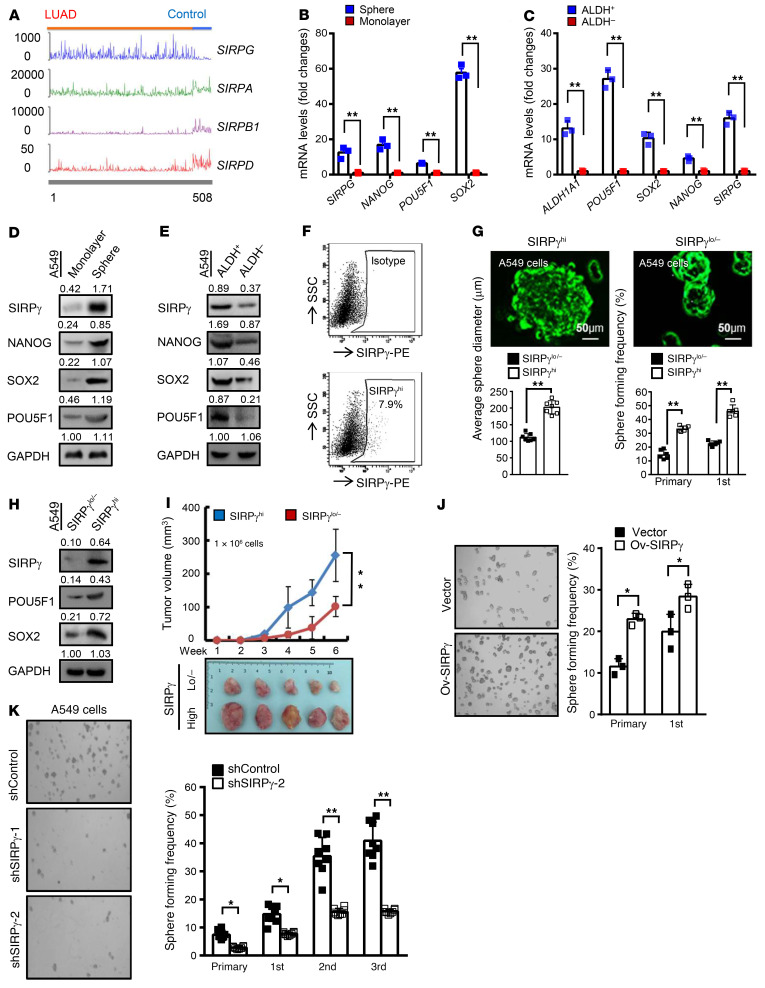
SIRPγ serves as a CSLC marker and promotes tumor growth. (**A**) The normalized gene expression values of *SIRPG*, *SIRPA*, *SIRPB1*, and *SIRPD* in LUAD and adjacent normal tissues. (**B** and **C**) qRT-PCR analysis of the indicated genes in A549 cells grown in monolayer or spheres (**B**) or ALDH^+^ and ALDH^–^ A549 cells (**C**). (**D** and **E**) Immunoblotting analysis of indicated proteins in A549 monolayers and spheres (**D**) or ALDH^+^ and ALDH^–^ (**E**) A549 cells. (**F**) Flow cytometric analysis of the number of SIRPγ^hi^ A549 cells. (**G**) Stem cell sphere assay of SIRPγ^lo/–^ and SIRPγ^hi^ A549 cells (1 × 10^3^ cells/well). (**H**) Immunoblotting analysis of indicated proteins in SIRPγ^lo/–^ and SIRPγ^hi^ A549 cells. (**I**) Tumor xenograft growth of SIRPγ^hi^ vs. SIRPγ^lo/–^ A549 cells (1 × 10^6^ inoculated cells/mouse, mean ± SD, *n* = 5 mice). (**J**) Stem cell sphere assay of vector- and SIRPγ-overexpressing A549 cells (1 × 10^3^ cells/well). (**K**) Stem cell sphere assay of control and SIRPγ-knockdown A549 cells (1 × 10^3^ cells/well). All experiments were carried out at least in triplicate and the data are presented as the mean ± SD or mean ± SD. *P < 0.05; **P < 0.01 by paired or unpaired, 2-tailed Student’s *t* test. See complete unedited blots in the supplemental material.

We used The Cancer Genome Atlas (TCGA) data set to focus on differential expression analysis of the SIRP family members in 450 lung adenocarcinoma (LUAD) tumor samples, along with 50 adjacent normal tissues (TCGA LUAD data set was downloaded from GDC Data Portal at https://portal.gdc.cancer.gov/projects/TCGA-LUAD). This revealed marked upregulation of *SIRPG* mRNA in the lung tumors, while the other family members, *SIRPA*, *SIRPB1*, and *SIRPD*, were downregulated (Figure 1A). To identify a targetable CSLC surface marker that plays a critical role in maintaining cancer stemness, we performed transcriptomic analysis to identify genes preferentially expressed in CSLCs, enriched by growth in nonadherent sphere cultures relative to adherent monolayer cultures (Supplemental Figure 1A; supplemental material available online with this article; https://doi.org/10.1172/JCI141797DS1). As an independent method to enrich the CSLC population, we used flow cytometry and a fluorogenic enzyme substrate to select cells expressing aldehyde dehydrogenase (ALDH^+^) (24–26). Utilizing both fractionation methods with the established A549 and H1975 human LUAD cell lines, we found that the level of *SIRPG* mRNA was markedly higher in the CSLC-enriched populations compared with control unfractionated monolayer cells or ALDH^–^ cells (Figure 1, B and C, and Supplemental Figure 1, B and C). The relative increase in *SIRPG* mRNA expression, in the range of 10- to 15-fold, exceeds that seen for several transcription factors that are typically associated with CSLCs, namely *POU5F1* (*OCT4*), *SOX2*, and *NANOG*. Immunoblotting confirmed that SIRPγ protein, along with the POU5F1, SOX2, and NANOG proteins, is overexpressed in the A549 and H1975 cells selected by both methods (Figure 1, D and E, and Supplemental Figure 1, D and E).

**Figure 2:**
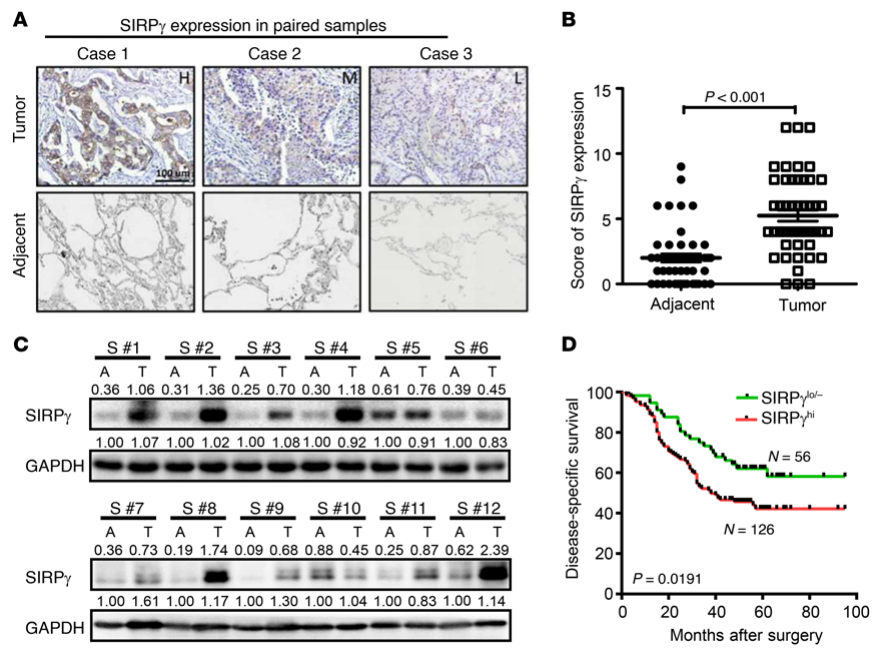
SIRPγ is highly expressed in LUAD and predicts the poor prognosis of patients. (**A**) IHC staining of SIRPγ in LUAD and adjacent normal tissues from 50 cases of patients with LUAD. Scale bar: 100 μm. (**B**) Expression score of SIRPγ in LUAD and adjacent normal tissues. (**C**) Immunoblotting analysis of SIRPγ expression in 12 fresh LUAD and adjacent normal tissues. (**D**) High SIRPγ expression is correlated with poor survival of patients with LUAD. Data were analyzed by paired, 2-tailed Student’s *t* test (**B**) or log-rank test for survival (**D**).

Until recently, SIRPγ was recognized as presenting mainly on T cells, some B cells, and activated NK cells (13). However, the function of SIRPγ and its signaling mechanism remain unknown. Although earlier studies have not revealed expression and functions of SIRPγ in any cancer types, the discovery of increased *SIRPG* mRNA in LUAD CSLCs led us to search for a possible role in tumor progression (27). Data from the Broad Firehose (http://firebrowse.org) showed that *SIRPG* is upregulated in 15 of 36 cancer types (Supplemental Figure 1F). We then performed qRT-PCR and Western blot assays using various commercial antibodies against SIRPγ to validate the expression of SIRPγ in lung cancer cells (Supplemental Figure 2, A–D). Although *SIRPG* mRNA expression was much higher in immune cells than A549 and H1975, its mRNA and protein expression was highly enriched in cancer spheres compared with the monolayer culture (Supplemental Figure 2, E, F, and J). We verified the specificity of various commercial SIRPγ antibodies, which recognized recombinant SIRPγ but not SIRPα, in a dot blot assay (Supplemental Figure 2I) and detected obvious SIRPγ protein expression in control NSCLC A549 and H1975 cells, but not in SIRPγ-knockdown cells in Western blot assays (Supplemental Figure 2, B and C). Overexpression of *SIRPG* markedly enhanced the SIRPγ signal (Supplemental Figure 2D). We also assessed SIRPγ protein expression semiquantitatively by immunohistochemistry in specimens from a cohort of 182 LUAD patients followed clinically for more than 9 years and set objective criteria for high (SIRPγ^hi^) versus low (SIRPγ^lo/–^) expression phenotypes. We consistently observed elevated SIRPγ-specific staining in the LUAD tissues compared with adjacent normal tissues (Figure 2, A and B). Immunoblotting of 12 fresh LUAD specimens also confirmed higher SIRPγ protein expression in tumors relative to adjacent nontumor tissues (Figure 2C and Supplemental Table 1). Significantly, Kaplan-Meier analysis indicated that high expression of SIRPγ protein in LUADs correlates with poorer disease-specific survival (Figure 2D and Supplemental Table 2).

The enhanced expression of SIRPγ in enriched A549 and H1975 CSLCs together with the clinical data in LUAD patients led us to hypothesize that the SIRPγ protein contributes to stem cell maintenance and LUAD progression. Therefore, we used flow cytometry with a monoclonal antibody (mAb) specific for SIRPγ to select A549 and H1975 cells expressing this protein and tested them for CSLC phenotypic characteristics. By this method we found that 7.9% of A549 monolayer-grown cells and 5.1% of cells from another human LUAD cell line, H1975, express SIRPγ, while CD47 expression was detected in the majority of A549 and H1975 cell populations (Figure 1F and Supplemental Figure 1G). Consistent with CSLCs, the sorted SIRPγ^hi^ A549 and H1975 cells showed increased expression of POU5F1, SOX2, CD133, and CD44, and also formed more and larger spheres in nonadherent culture than the sorted SIRPγ-negative (SIRPγ^lo/–^) fraction of A549 and H1975 cells (Figure 1, G and H, and Supplemental Figure 1, H and K). When equal numbers of cells (1 × 10^6^) were inoculated into immune-deficient mice, the SIRPγ^hi^ cells displayed accelerated tumor formation compared with SIRPγ^lo/–^ cells (Figure 1I). We also performed limiting-dilution assays to estimate the frequency of tumor-initiating cells in the SIRPγ^hi^ and SIRPγ^lo/–^ A549 populations. We found that the SIRPγ^hi^ population, but not the SIRPγ^lo/–^ population, gave rise to tumors in a majority of the recipient mice at a dose of 50,000 cells (Supplemental Figure 1I and Supplemental Table 3). Thus, SIRPγ^hi^ cells represent CSLC populations with tumorigenic potential. These results indicate that SIRPγ is likely a CSLC marker for NSCLC.

To determine whether SIRPγ is important for CSLC function, we carried out complementary overexpression and knockdown experiments. We observed that overexpression of SIRPγ via a lentiviral expression vector increased sphere formation in nonadherent cultured cells (Figure 1J). Conversely, knockdown of *SIRPG* using lentiviral shRNAs reduced sphere formation (Figure 1K). In the in vivo xenograft model, we observed that knockdown of SIRPγ inhibited tumor growth (Supplemental Figure 1J). To determine whether SIRPγ is likely a general CSLC marker, we conducted qRT-PCR and Western blot analyses in a variety of cancer cell lines with distinct cancer cell origins and found that *SIRPG* mRNA and protein expression was also enriched in cancer spheres compared with the monolayer culture (Supplemental Figure 2, F and J). Moreover, knockdown of SIRPγ in the liver cancer cell line LM3 also impaired cancer sphere formation (Supplemental Figure 2G), while its overexpression promoted it (Supplemental Figure 2H). Collectively, our data with human lung cancer cell lines validate SIRPγ as a putative CSLC marker and indicate that this protein positively regulates key elements of the stem cell phenotypes, including tumorigenicity.

### SIRPγ serves as a negative upstream regulator of the MST1/LATS1 axis to promote YAP activation and cancer organoid growth.

To investigate the molecular mechanisms by which SIRPγ might impact CSLC biology, we performed a systematic bioinformatics analysis of gene-gene interaction networks based on mutations, copy number alterations, mRNA expression profiles, and protein expression profiles from 522 TCGA LUAD samples using the cBio Cancer Genomics Portal (http://cbioportal.org). We observed that SIRPγ and the transcriptional regulator YAP1 (Yes-associated protein 1, YAP65) are located in a network containing 53 nodes (Supplemental Figure 3A), suggesting a potential link between SIRPγ and YAP signaling. In support of this hypothesis, unbiased transcriptomic analysis revealed that numerous YAP target genes, including *BIRC5*, *LAMC2*, *MMP10*, *SLUG*, *SOX2*, *GLI2*, and *CYR61* were repressed when shRNA was used to knock down SIRPγ expression in A549 cells (Supplemental Table 4).

YAP acts as an oncoprotein; its signaling plays a critical role in CSLCs, cancer progression, and metastasis by inducing the expression of diverse target genes involved in biological processes such as cell polarity, survival, epithelial-mesenchymal transition, and cell migration (28–31). YAP functions as the crucial nuclear effector of the Hippo signaling pathway. Its activity is modulated by an upstream protein kinase cascade that controls its translocation from the cytoplasm (inactive) to the nucleus (active). Phosphorylation by the Hippo kinase large tumor suppressor kinase 1 (LATS1) keeps YAP sequestered in the cytoplasm. LATS1 kinase activity depends on phosphorylation by (macrophage stimulating 1 (MST1; also known as serine/threonine kinase 4, STK4). Thus, the MST1/LATS1 axis inhibits YAP signaling activation by triggering YAP phosphorylation and nuclear exclusion (32–34). Although it is known that the Hippo kinases are regulated by extracellular cues such as cell-cell contact, the underlying mechanisms accounting for their activation and inactivation remain elusive. Adaptor proteins such as Merlin and Scribble participate in sensing extracellular signals to induce MST1/LATS1 activation and consequent YAP inactivation (29). However, less is known about upstream signals and regulators that may inhibit the MST1/LATS1 axis to promote active YAP signaling.

**Figure 3:**
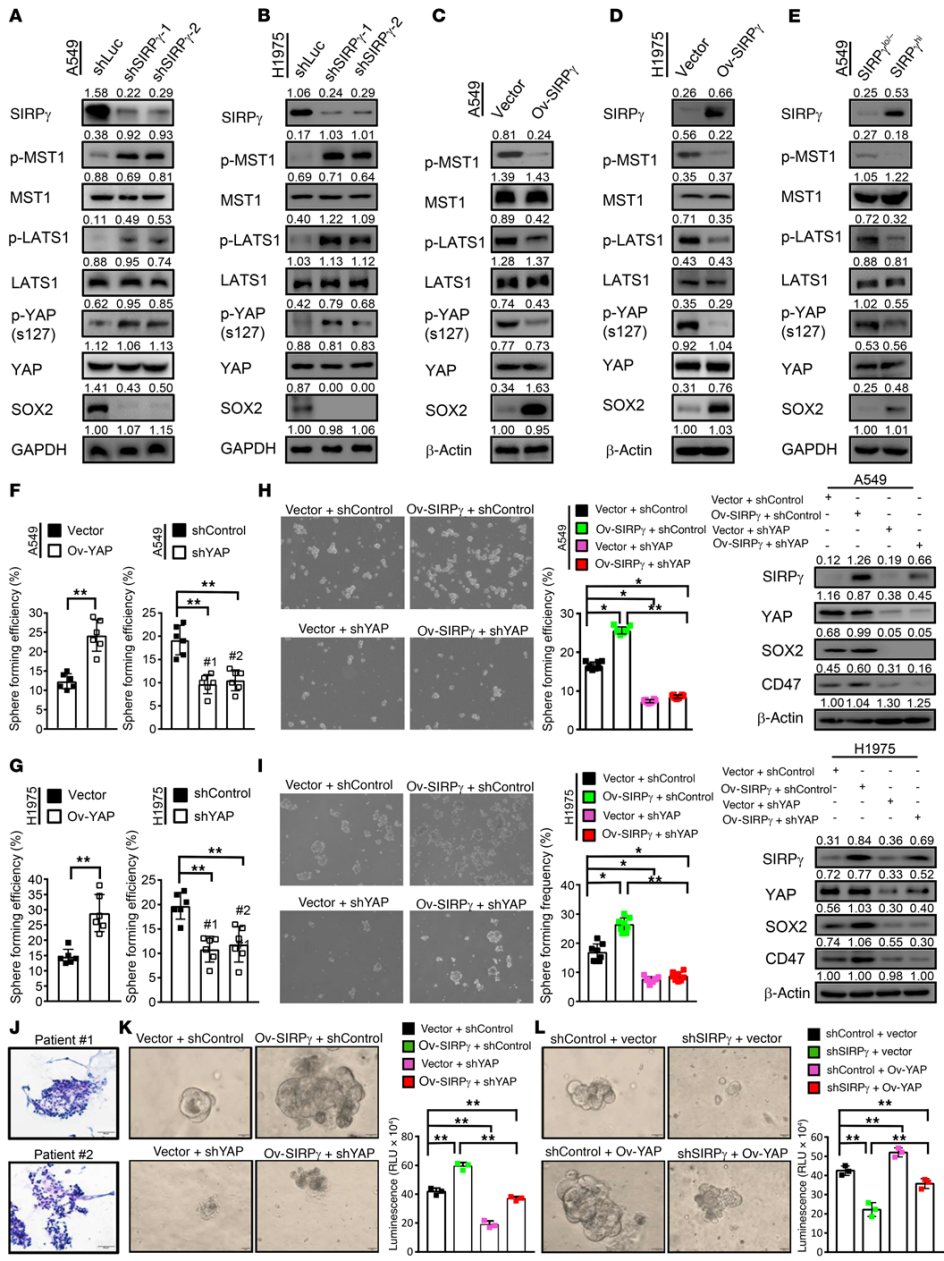
SIRPγ serves as a negative upstream regulator of the MST1/LATS1 axis to promote YAP activation and cancer organoid growth. (**A** and **B**) Immunoblotting analysis of indicated proteins in control and SIRPγ-knockdown A549 (**A**) and H1975 (**B**) cells. (**C** and **D**) Immunoblotting analysis of indicated proteins in control and SIRPγ-overexpressing cells. (**E**) Immunoblotting analysis of indicated proteins in SIRPγ^lo/–^ and SIRPγ^hi^ A549 cancer cells. (**F** and **G**) Stem cell sphere assay of YAP-overexpressing or -knockdown A549 (**F**) or H1975 (**G**) cells (1 × 10^3^ cells/well). (**H** and **I**) Stem cell sphere formation assay and immunoblotting analysis of vector- and SIRPγ-overexpressing A549 (**H**) or H1975 (**I**) cells with or without YAP knockdown. (**J**) H&E staining of tumor tissue derived from patients with LUAD. Scale bars: 50 μm. (**K** and **L**) Representative images are shown for the growth of LUAD-derived organoids of indicated groups grown in matrigel-supplemented media for 7 days. Quantification of the growth of organoids. Scale bars: 20 μm. All experiments were carried out at least in triplicate and the data are presented as the mean ± SD. *P < 0.05; **P < 0.01 by paired, 2-tailed Student’s *t* test (**F** and **G**, left panels) or 1-way ANOVA (**F**, **G** [right panels in both], **H**, **I**, **K**, and **L**).

We asked whether SIRPγ could serve as a negative upstream regulator of the MST1/LATS1 axis and thereby promote YAP activation. Consistent with this idea, we observed that knockdown of SIRPγ in A549 and H1975 cells led to enhanced phosphorylation of MST1 (p-MST1), LATS1 (p-LATS1), and YAP (p-YAP), accompanied by reduced expression of the YAP target SOX2 (Figure 3, A and B). SIRPγ overexpression decreased levels of the phosphorylated proteins p-MST1, p-LATS1, and p-YAP, leading to enhanced expression of SOX2 (Figure 3, C and D). Furthermore, SIRPγ^hi^ cancer cells displayed decreased p-MST1, p-LATS1, and p-YAP, and enhanced SOX2 expression compared with SIRPγ^lo/–^ cells (Figure 3E).

As a transcriptional coactivator, YAP is activated upon its dephosphorylation and translocation from the cytosol to the nucleus, where it cooperates with DNA-binding transcription factors, mainly members of the TEAD family, to drive the expression of its targets genes (29, 35). Consistent with the observed increase in YAP phosphorylation, we observed that knockdown of SIRPγ significantly decreased the amount of YAP in the nucleus by immunofluorescence. Similarly, the amount of nuclearly localized SOX2 also decreased substantially in SIRPγ-knockdown cells (Supplemental Figure 3, H–O).

As the YAP/SOX2 axis is required for CSLC self-renewal, we asked whether SIRPγ acts through YAP signaling to promote CSLC properties. In line with this idea, we found that YAP overexpression increased sphere formation, whereas its knockdown had the opposite effect in 2 NSCLC cell lines (Figure 3, F and G). While SIRPγ overexpression increased SOX2 expression and promoted CSLC phenotypes, YAP depletion compromised this effect (Figure 3, H and I). These data collectively suggest that SIRPγ is an upstream inhibitor of Hippo kinases to maintain YAP signaling activation, thereby promoting CSLC properties.

We then conducted experiments to define the role of SIRPγ in the growth of organoids from NSCLC patient–derived tumors and found that SIRPγ or YAP overexpression promoted cancer organoid growth, while SIRPγ or YAP knockdown reduced it (Figure 3, J–L). Of note, we found that YAP knockdown compromised the promoting effect of SIRPγ on cancer organoid growth (Figure 3K and Supplemental Figure 3F). Overexpression of YAP sufficed to overcome the inhibition of organoid growth caused by SIRPγ knockdown (Figure 3L and Supplemental Figure 3G). We concluded that SIRPγ acts through YAP signaling to promote tumor growth.

### SIRPγ recruits PP2A to dephosphorylate MST1 and promote YAP signaling activation.

**Figure 4:**
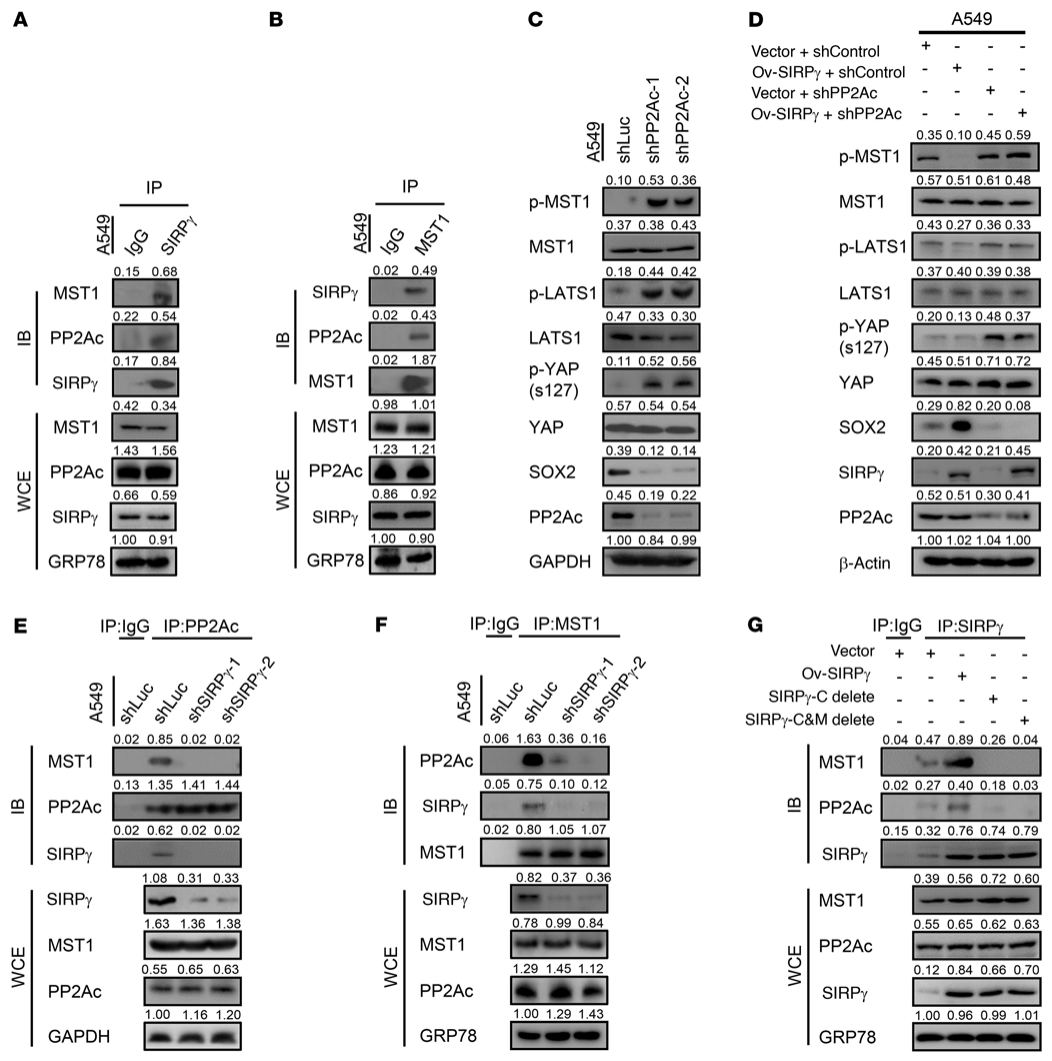
SIRPγ recruits PP2A to dephosphorylate MST1 and promotes YAP signaling activation. (**A** and **B**) Immunoprecipitation analysis of the interaction between SIRPγ, PP2A, and MST1. PP2Ac is the catalytic subunit of PP2A. IB, immunoblot; WCE, whole-cell extract. (**C**) Immunoblotting analysis of indicated proteins in control and PP2A-knockdown cells. (**D**) Immunoblotting analysis of indicated proteins in vector- and SIRPγ-overexpressing cells with or without PP2Ac knockdown in A549 cells. (**E** and **F**) Immunoprecipitation analysis of the interaction between PP2A and MST1 in control and SIRPγ-knockdown A549 cells. (**G**) Immunoprecipitation analysis of the interaction between SIRPγ, PP2A, and MST1 in vector-, SIRPγ-, SIRPγ C terminus–, or SIRPγ C terminus and transmembrane domain (C&M) deletion mutant–overexpressing A549 cells.

To dissect the mechanism by which SIRPγ inhibits Hippo kinases for YAP activation, we pulled down SIRPγ or MST1 from A549 and H1975 cell lysates using specific antibodies to seek evidence for a complex containing these 2 proteins. We observed reciprocal coimmunoprecipitation of endogenous SIRPγ and MST1, indicating that these proteins interact. Interestingly, protein phosphatase 2A (PP2A), which dephosphorylates and inactivates MST1 (29, 35, 36), was also detected in both SIRPγ and MST1 immunocomplexes (Figure 4, A and B, and Supplemental Figure 3, B and C).

The coimmunoprecipitation data led us to hypothesize that SIRPγ may serve as a bridging factor that recruits PP2A to inactivate MST1, ultimately leading to activation of YAP and YAP-dependent transcriptional activation. We first tested the effect of PP2A knockdown in A549 and H1975 cells to confirm its key role in regulating the phosphorylation status of components of the Hippo/YAP signaling cascade. Depletion of PP2A expression increased the levels of phosphorylated MST1, LATS1, and YAP. As would be expected, this correlated with strong inhibition of expression of the YAP-dependent factor SOX2 (Figure 4C). We then asked whether the ability of SIRPγ to activate Hippo/YAP signaling depends on PP2A. As shown in Figure 4D, overexpression of SIRPγ inhibited phosphorylation of MST1, LATS1, and YAP and stimulated expression of SOX2. However, these effects were lost entirely in SIRPγ-overexpressing cells treated with shRNA that knocks down PP2A; in this case, the phenotype was identical to cells treated with the *PP2A* shRNA alone, without SIRPγ overexpression. We carried out further immunoprecipitation studies to determine whether SIRPγ regulates the formation of a protein complex involving PP2A and its substrate MST1. Precipitation of cancer cell lysates with an antibody against PP2A also enriched for precipitated MST1 and SIRPγ. However, in cells with SIRPγ knockdown, the precipitates generated by the PP2A antibody no longer showed enrichment for MST1 (Figure 4E and Supplemental Figure 3D). Similarly, immunoprecipitates obtained with an anti-MST1 antibody in the SIRPγ-knockdown cells showed greatly decreased levels of coprecipitated PP2A (Figure 4F and Supplemental Figure 3E). Moreover, by conducting domain-mapping experiments, we found that the C-terminal region of SIRPγ and the region of MST1 that includes amino acids 433–486 are required for the SIRPγ, MST1, and PP2A interaction (Figure 4G and Supplemental Figure 4, A and B).

Taken together, our data support a model in which SIRPγ directly mediates the interaction of MST1 with PP2A. SIRPγ thus acts at an early upstream step to negatively control the Hippo signaling cascade by promoting dephosphorylation of MST1 kinase, thereby limiting phosphorylation of LATS1 and YAP. The net result is increased nucleus-associated YAP, leading to enhanced expression of CSLC- and cancer-promoting genes like *SOX2*.

### SIRPγ promotes cytokine release to sustain CD47 expression through YAP signaling.

**Figure 5:**
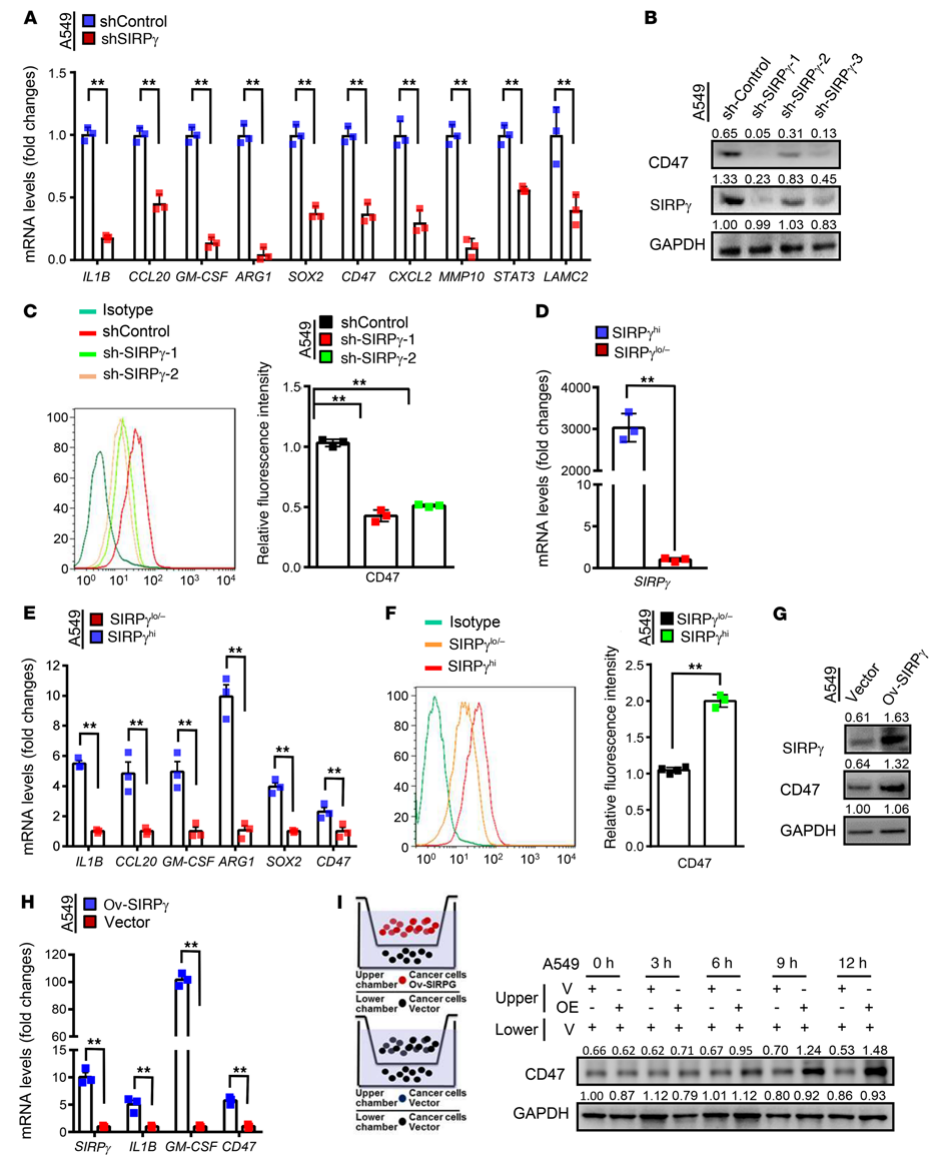
SIRPγ sustains CD47 expression. (**A**) qRT-PCR analysis of indicated genes in control and SIRPγ-knockdown A549 cells. (**B**) Immunoblotting analysis of indicated proteins in control and SIRPγ-knockdown A549 cells. (**C**) Flow cytometric analysis of CD47 in control and SIRPγ-knockdown A549 cells. (**D**) qRT-PCR analysis of *SIRPG* in SIRPγ^hi^ and SIRPγ^lo/–^ A549 cells. (**E**) qRT-PCR analysis of indicated genes in SIRPγ^hi^ and SIRPγ^lo/–^ A549 cells. (**F**) Flow cytometric analysis of CD47 protein expression in SIRPγ^lo/–^ and SIRPγ^hi^ A549 cells. (**H**) qRT-PCR analysis of indicated genes in vector- and SIRPγ-overexpressing A549 cells. (**G**) Immunoblotting analysis of indicated proteins in vector- and SIRPγ-overexpressing A549 cells. (**I**) Left: Schematic diagram of vector- (V, control) and SIRPγ-overexpressing (OE) A549 cell coculture. Right: Immunoblotting analysis of indicated proteins in control cells cocultured with control cells and control cells cocultured with SIRPγ-overexpressing cells. All experiments were carried out at least in triplicate and the data are presented as the mean ± SD. **P < 0.01 by paired, 2-tailed Student’s *t* test (**A**, **D**–**F**, and **H**) or 1-way ANOVA (**C**).

CD47 has been identified as a key component of a checkpoint inhibitor for the innate immune system. Cancer cells can escape macrophage-mediated phagocytosis through expression of CD47, which interacts with SIRPα on the surface of macrophages to trigger the “don’t eat me” signal (3). It was therefore of particular interest that A549 cells with SIRPγ knockdown show significant downregulation of CD47. Assessment of our quantitative PCR data also showed decreased expression of a number of additional genes involved in cell proliferation, cytokine–cytokine receptor interaction, apoptosis, cell death, and wound response in SIRPγ-knockdown cells (Figure 5A). We validated these observations for genes such as *CD47* shown in Supplemental Figure 5A. Downregulation of *CD47* mRNA and protein expression in response to knockdown of SIRPγ was confirmed by qRT-PCR, immunoblotting, and flow cytometric analyses (Figure 5, B and C, and Supplemental Figure 5, A and D). By contrast, *CD47* was among a set of genes showing increased expression in SIRPγ^hi^ A549 and H1975 cells compared with SIRPγ^lo/–^ cells selected by flow cytometry (Figure 5, D–F, and Supplemental Figure 5B) and SIRPγ-overexpressing cancer cells (Figure 5, G and H, and Supplemental Figure 5, C and E).

Because we observed detectable surface SIRPγ protein expression in less than 10% of A549 and H1975 cells cultured under standard conditions, comprising the CSLC populations, we were puzzled to account for how SIRPγ might control CD47 expression by the large majority of (non-stem) cancer cells. We hypothesized that the SIRPγ^hi^ CSLCs may regulate CD47 expression in CSLCs and bulk cancer cell population through autocrine or paracrine signaling, particularly as previous studies revealed that cytokines such as TNF-α and IL-1β can stimulate CD47 expression (37). We employed Transwell coculture experiments and found that SIRPγ overexpression in A549 and H1975 cells (representing SIRPγ^hi^ cells) in comparison with vector expression in cancer cells (representing SIRPγ^lo/–^ cells) in the upper chamber showed increased CD47 expression in vector-expressing A549 and H1975 cells (representing SIRPγ^lo/–^ cells) from the lower chamber in a time-dependent manner (Figure 5I and Supplemental Figure 5F), suggesting that the SIRPγ^hi^ cancer cell population transmits a paracrine signal to induce protein CD47 expression in the SIRPγ^lo/–^ cancer cell population.

**Figure 6:**
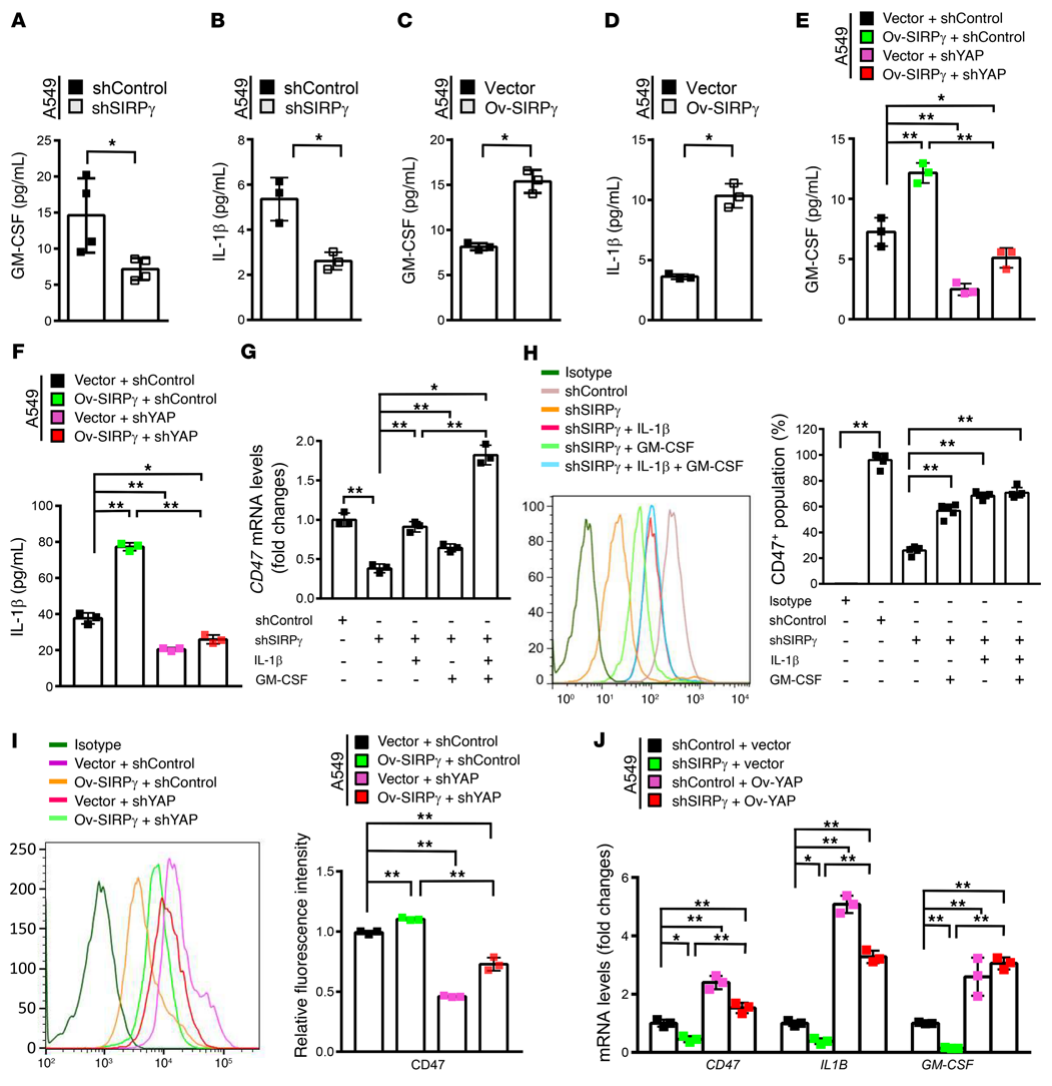
SIRPγ promotes IL-1β and GM-CSF release to sustain CD47 expression through YAP signaling. (**A** and **B**) ELISA analysis of indicated cytokines in control and SIRPγ-knockdown A549 cells. (**C** and **D**) ELISA analysis of indicated cytokines in vector- and SIRPγ-overexpressing A549 cells. (**E** and **F**) ELISA analysis of indicated cytokines in vector- and SIRPγ-overexpressing cells with or without YAP knockdown. (**G**) qRT-PCR analysis of *CD47* expression in control and SIRPγ-knockdown cells with or without IL-1β (100 ng/mL) and GM-CSF (30 ng/mL) treatment for 24 hours. (**H**) Flow cytometric analysis of CD47 expression in control and SIRPγ-knockdown cells with or without IL-1β (100 ng/mL) and GM-CSF (30 ng/mL) treatment for 24 hours. (**I**) Flow cytometric analysis of CD47 protein expression in vector- and SIRPγ-overexpressing A549 cells with or without YAP knockdown. (**J**) qRT-PCR analysis of indicated genes in control and SIRPγ-knockdown cells with or without YAP overexpression. All experiments were carried out at least in triplicate and the data are presented as the mean ± SD. *P < 0.05; **P < 0.01 by paired, 2-tailed Student’s *t* test (**A**–**D**) or 1-way ANOVA (**E**–**J**).

Transcript analysis showed that mRNAs for a group of cytokines including IL-1β and GM-CSF (also known as CSF-2) were downregulated upon SIRPγ knockdown (Figure 5A and Supplemental Figure 5A). This phenomenon was confirmed by Western blot assay and ELISA analysis showed decreased protein expression of IL-1β and GM-CSF in A549 and H1975 cells upon SIRPγ knockdown (Figure 6, A and B, and Supplemental Figure 5, A and D). Consistently, cells in which we overexpressed SIRPγ displayed enhanced expression and secretion of IL-1β and GM-CSF, along with increased surface expression of CD47 (Figure 5, H and I, Figure 6, C–F, and Supplemental Figure 5, C and F). Of note, we found that IL-1β and GM-CSF were selectively expressed in the sorted SIRPγ^hi^ cell population (Figure 5, E and F, and Supplemental Figure 5B). Notably, the addition of exogenous IL-1β and GM-CSF together rescued the decrease in *CD47* mRNA and protein expression caused by SIRPγ knockdown, as determined by qRT-PCR and flow cytometry analysis (Figure 6, G and H).

Gene-gene interaction network analysis revealed that both *SIRPG* and *YAP* display potential connections to *CD47* (Supplemental Figure 3A), raising the possibility that SIRPγ may regulate cytokine-dependent CD47 expression and phagocytosis through YAP signaling. We therefore investigated whether SIRPγ acts through the Hippo/YAP pathway to regulate the expression of IL-1β and GM-CSF. We observed that SIRPγ overexpression enhanced IL-1β and GM-CSF cytokine and CD47 expression, whereas YAP knockdown compromised this effect (Figure 6, E, F, and I). Overexpression of YAP rescued the defect in the expression of IL-1β and GM-CSF cytokines and CD47 expression in SIRPγ-knockdown cancer cells (Figure 6J), supporting the notion that SIRPγ acts through the Hippo/YAP pathway to regulate the expression of IL-1β, GM-CSF, and CD47. Our data therefore support the notion that SIRPγ^hi^ cancer cells, which are CSLCs, can maintain CD47 expression in bulk cancer cells through autocrine/paracrine signaling.

### SIRPγ helps cancer cells to escape from phagocytosis by macrophages through YAP signaling.

**Figure 7:**
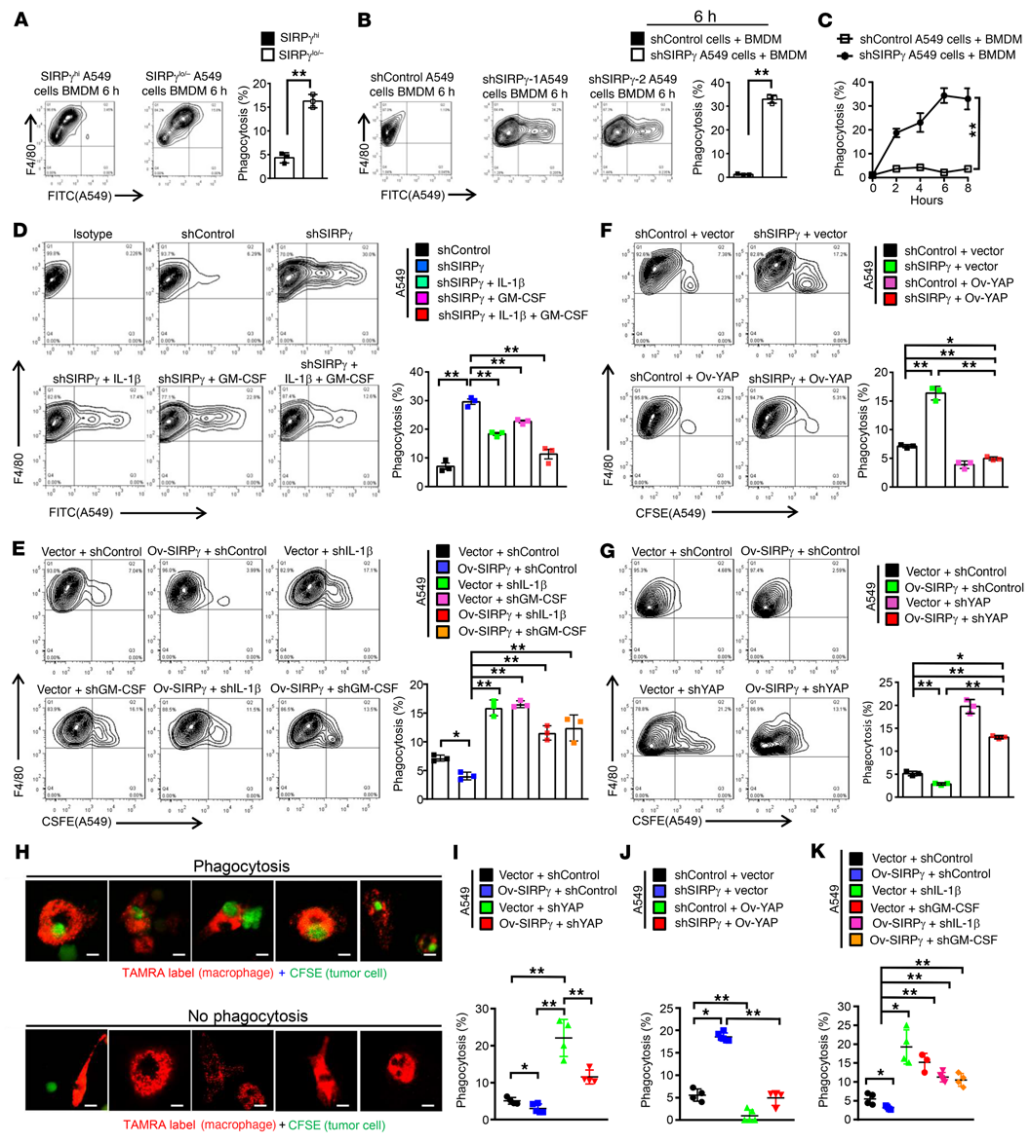
SIRPγ helps cancer cells to escape from phagocytosis by macrophages through YAP signaling. (**A**) Phagocytosis of SIRPγ^lo/–^ and SIRPγ^hi^ A549 cells by bone marrow–derived macrophages (BMDMs). (**B**) Flow cytometric analysis of phagocytosis of control and SIRPγ-knockdown A549 cells (2 × 10^4^ cells/tube) by BMDMs (2 × 10^4^ cells/tube). (**C**) Statistical analysis of phagocytosis of control and SIRPγ-knockdown A549 cells (2 × 10^4^ cells/tube) by BMDMs (2 × 10^4^ cells/tube). (**D**) Phagocytosis of control and SIRPγ-knockdown cells with or without IL-1β (100 ng/mL) and GM-CSF (30 ng/mL) treatment for 24 hours. (**E**) Phagocytosis of vector- and SIRPγ-overexpressing CFSE-labeled A549 cells with or without IL-1β or GM-CSF knockdown. (**F**) Phagocytosis of control and SIRPγ-knockdown CFSE-labeled A549 cells with or without YAP overexpression. (**G**) Phagocytosis of vector- and SIRPγ-overexpressing CFSE-labeled A549 cells with or without YAP knockdown. BMDMs were induced with 50 ng/mL M-CSF for 7 days. (**H**) Phagocytosis of CFSE-labeled A549 cells by PKH26-labeled BMDMs was assessed by confocal microscopy. Red, macrophages; green, targets. Scale bars: 50 μm. (**I**) Phagocytosis assay of vector- and SIRPγ-overexpressing A549 cells with or without YAP knockdown based on CFSE-labeled A549 cells and PKH26-labeled BMDMs. (**J**) Phagocytosis assay of control and SIRPγ-knockdown A549 cells with or without YAP overexpression based on CFSE-labeled A549 cells and PKH26-labeled BMDMs. (**K**) Phagocytosis assay of vector- and SIRPγ-overexpressing A549 cells with or without IL-1β or GM-CSF knockdown based on CFSE-labeled A549 cells and PKH26-labeled BMDMs. All experiments were carried out at least in triplicate and the data are presented as the mean ± SD. *P < 0.05; **P < 0.01 by paired, 2-tailed Student’s *t* test (**A**–**C**) or 1-way ANOVA (**D**–**G** and **I**–**K**).

In light of our observation that SIRPγ is critical for CD47 expression in CSLCs, we asked whether SIRPγ expression influences phagocytosis of the cancer cells by macrophages. We sorted A549 and H1975 human LUAD cells using an antibody against SIRPγ, labeled the cells with GFP, and assessed the rate of their phagocytosis by RFP-labeled human bone marrow–derived macrophages (BMDMs) by flow cytometry, using the simultaneous presence of green and red labels to identify macrophages that had taken up a cancer cell. We observed that the BMDMs phagocytosed SIRPγ^lo/–^ cells significantly more rapidly compared with the SIRPγ^hi^ cells (Figure 7A and Supplemental Figure 6A). Furthermore, SIRPγ knockdown in the A549 cells significantly promoted their phagocytosis by BMDMs (Figure 7, B and C). We repeated the phagocytosis assay using 2 additional methods, carboxytetramethylrhodamine (TAMRA) and carboxyfluorescein succinimidyl ester (CFSE) fluorescent cell staining (38–40), and we observed consistent results between these methods (Figure 7H and Supplemental Figure 6, B and C). These findings suggest that SIRPγ not only plays a critical role in determining CSLC phenotype, but also helps cancer cells to escape from phagocytosis by macrophages. The increase in CD47 by exogenously supplied IL-1β and GM-CSF also sufficed to overcome the enhanced sensitivity of cancer cells to phagocytosis caused by knockdown of SIRPγ (Figure 7D).

Therefore, we investigated whether SIRPγ acts through the Hippo/YAP/CD47 pathway to affect phagocytosis. We observed that overexpression of YAP rescued the defect in the expression of IL-1β and GM-CSF cytokines and CD47 expression in SIRPγ-knockdown cancer cells (Figure 6J), and also reversed heightened phagocytosis of the SIRPγ-knockdown cancer cells by macrophages (Figure 7, F and J, and Supplemental Figure 6, D and H). While SIRPγ overexpression enhanced IL-1β, GM-CSF, and CD47 expression and inhibited phagocytosis, YAP knockdown compromised this effect (Figure 7, G and I, and Supplemental Figure 6, E and G). Moreover, depletion of IL-1β or GM-CSF by shRNA knockdown abrogated the effect of SIRPγ overexpression on reducing sensitivity of cancer cells to phagocytosis (Figure 7, E and K, and Supplemental Figure 6, F and I).

Similar to YAP knockdown, CD47 knockdown enhanced phagocytosis, and also compromised the suppressive effect of SIRPγ overexpression on phagocytosis (Supplemental Figure 7, A, C, E, and G). Conversely, CD47 overexpression decreased the sensitivity of cancer cells to phagocytosis, leading to reversal of the increased phagocytosis of the SIRPγ-knockdown cancer cells by macrophages (Supplemental Figure 7, B, D, F, and H). These data indicate that SIRPγ expression in CSLCs activates YAP signaling to elicit IL-1β and GM-CSF cytokine release that can induce CD47 expression to inhibit phagocytosis in the general tumor cell population, thereby supporting the notion that SIRPγ^hi^ cancer cells enable tumors to bypass an important aspect of innate immune surveillance. We repeated the phagocytosis assay using THP1 cell–derived M1 macrophages, and the results between these methods were consistent (Supplemental Figure 8A). TAMRA and CFSE fluorescent cell staining by confocal microscopy showed that SIRPγ overexpression decreased the phagocytosis by M1 macrophages (Supplemental Figure 8B). SIRPγ knockdown in A549 cells significantly increased the phagocytosis by M1 macrophages (Supplemental Figure 8C). We further assessed the rate of phagocytosis by flow cytometry and found that SIRPγ or YAP1 knockdown in A549 and H1975 cells increased phagocytosis by M1 macrophages (Supplemental Figure 8, D and E). Furthermore, overexpression of YAP compromised the increased phagocytosis in SIRPγ-knockdown cancer cells (Supplemental Figure 8, F and G).

### The SIRPγ/YAP axis promotes tumor growth and metastasis.

**Figure 8:**
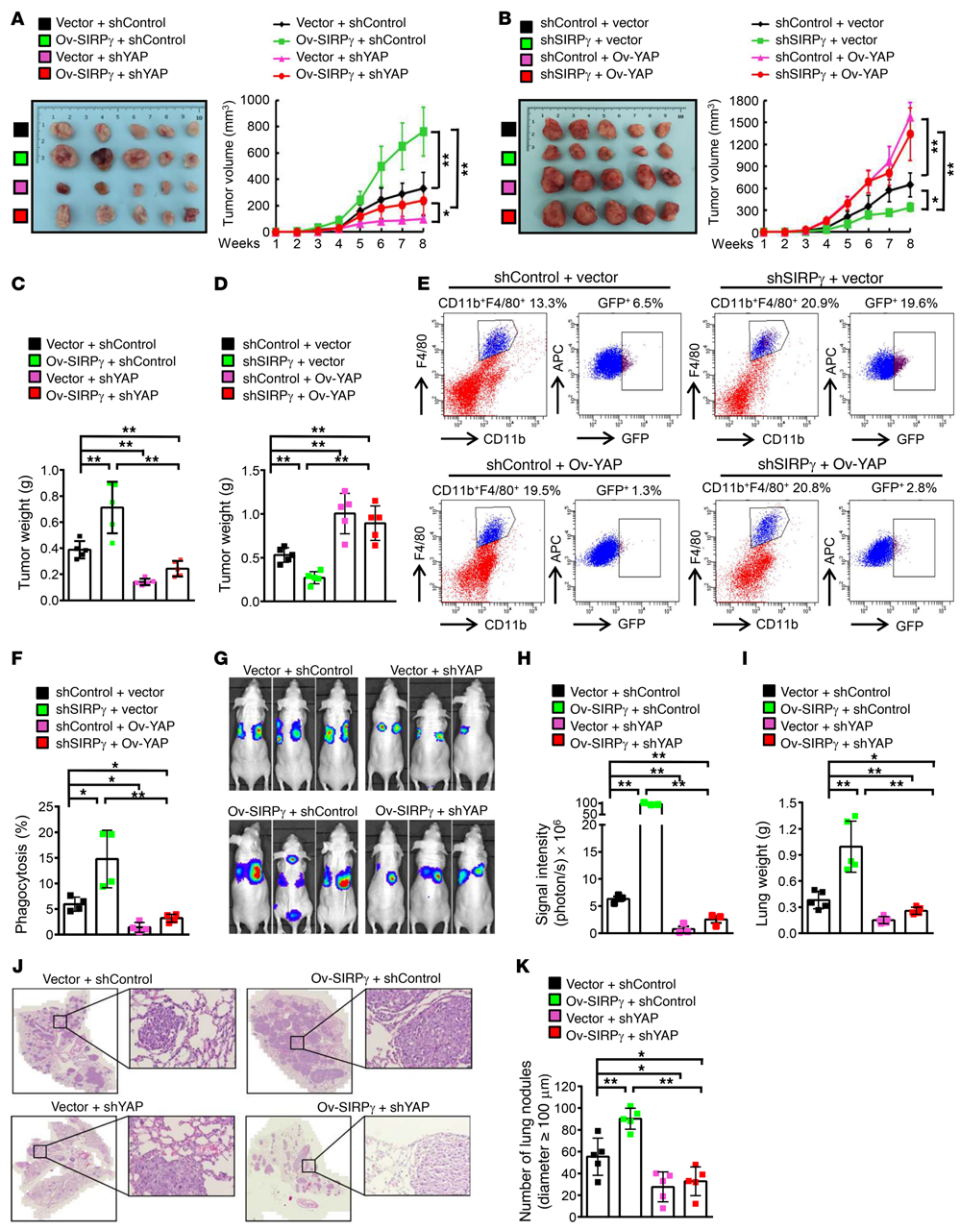
The SIRPγ/YAP axis promotes tumor growth and metastasis. (**A** and **B**) A549-xenograft growth of indicated groups (1 × 10^6^ inoculated cells/mice, n = 5 mice per group). (**C** and **D**) A549-xenograft weight of indicated groups. (**E** and **F**) Phagocytosis in A549 xenografts, represented by the percentage of GFP^+^F4/80^+^CD11b^+^ cells in total F4/80^+^CD11b^+^ cells. (**G** and **H**) A549 lung metastasis of indicated groups (1 × 10^5^ tail vein–injected cells per mouse). (**I**) Lung weight of indicated groups. (**J**) H&E staining of lung sections from indicated groups. Scale bars: 3000 μm and 50 μm (zoomed-in images on right). (**K**) The number of metastatic lung nodules of indicated groups. Cells were injected into the lateral tail vein of 6-week-old female nude mice (1 × 10^5^ cells per mouse, *n* = 5 mice per group). All experiments were carried out at least in triplicate and the data are presented as the mean ± SD. *P < 0.05; **P < 0.01 by 1-way ANOVA.

We next sought to determine whether YAP signaling is central to the ability of SIRPγ to regulate tumorigenesis in vivo. In accordance with this idea, we observed that SIRPγ overexpression promoted growth of human LUAD tumors in the xenograft model, while knockdown of YAP inhibited tumor growth and abrogated the tumor-promoting effect upon SIRPγ overexpression (Figure 8, A and C, and Supplemental Figure 9, A–C). Conversely, YAP restoration sufficed to rescue the defect in tumorigenesis caused by SIRPγ knockdown (Figure 8, B and D, and Supplemental Figure 9, D–F). We conclude that SIRPγ acts through YAP signaling to promote tumorigenesis.

To investigate whether the functional role of the SIRPγ/YAP axis in promoting tumor development results partly from inhibition of phagocytosis, we utilized GFP-labeled A549 cells to assess their engulfment by macrophages in vivo. We identified phagocytic events by flow cytometry, assessing the percentage of F4/80^+^CD11b^+^GFP^+^ cells among total F4/80^+^CD11b^+^ murine macrophages. We observed that SIRPγ knockdown in the A549 cells enhanced in vivo phagocytosis, while YAP overexpression inhibited phagocytosis. Notably, YAP restoration abrogated heightened phagocytosis upon SIRPγ deficiency (Figure 8, E and F), indicative of the role of the SIRPγ/YAP axis in suppressing phagocytosis in vivo.

To determine whether heightened phagocytosis partly accounts for tumor suppression upon SIRPγ knockdown, we performed an in vivo macrophage depletion assay using clodronate liposomes (41, 42) and found that depletion of macrophages partially rescued the reduction in tumorigenicity of SIRPγ-knockdown cancer cells (Supplemental Figure 10, A–E), supporting the notion that SIRPγ orchestrates tumorigenesis partly through inhibiting phagocytosis.

In support of the role of CD47 in SIRPγ-mediated tumorigenesis, we found that CD47 knockdown abrogated the ability of SIRPγ to promote in vivo tumorigenesis (Supplemental Figure 11, A and B). As IL-1β and GM-CSF are responsible for SIRPγ-mediated CD47 expression and phagocytosis inhibition, we performed rescued experiments in an in vivo tumorigenesis assay and found that adding back IL-1β or GM-CSF reversed the increase in phagocytosis and partially rescued the defect in tumorigenesis upon SIRPγ deficiency (Supplemental Figure 11, C–E), suggesting that IL-1β and GM-CSF are relevant downstream effectors for SIRPγ-mediated phagocytosis suppression and tumorigenesis.

We further assessed the impact of the SIRPγ/YAP axis on tumor metastasis, a property often associated with CSLCs. We administered luciferase-labeled A549 and H1975 cells to nude (*nu/nu*) mice by tail vein injection and 6 weeks later acquired bioluminescence images to identify lung metastases. We found that knockdown of either SIRPγ or YAP in A549 and H1975 cells markedly inhibited the tumor signal intensity and the number of tumor nodules formed in the lungs of recipient mice (Supplemental Figure 12, A and B) and correlated with reduced lung weight (Supplemental Figure 12C).

Notably, depletion of YAP by shRNA knockdown abrogated the tumor metastasis and increased lung weight promoted by SIRPγ overexpression (Figure 8, G–I, and Supplemental Figure 12, D–H) and correspondingly improved survival of the recipient mice (Supplemental Figure 12F). Quantitative assessment of tumor nodules by standard pathology (i.e., microscopic inspection of biopsy sections stained with hematoxylin and eosin [H&E]) confirmed the results obtained by in vivo imaging (Figure 8, J and K). Thus, along with other phenotypes described above, our data indicate that SIRPγ acts through YAP signaling to suppress phagocytosis and promote tumor growth and metastasis.

**Figure 9:**
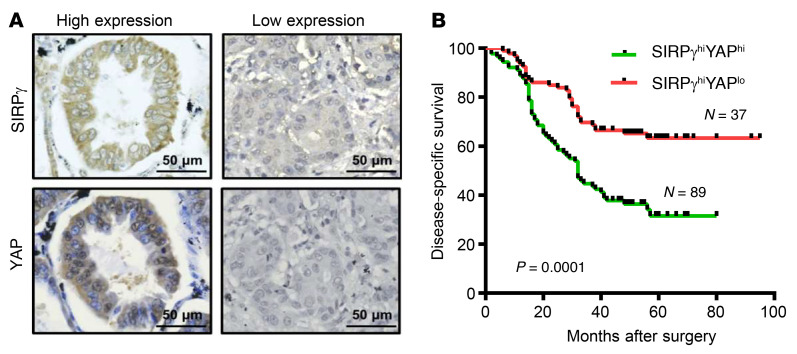
Overexpression of both SIRPγ and YAP predicts poor outcome of patients with LUAD. (**A**) Representative images of SIRPγ and YAP expression in LUAD samples. Scale bars: 50 μm. (**B**) High expression of both SIRPγ and YAP predicts poor survival of patients with LUAD. Log-rank test for survival.

Do our findings have relevance to human cancer? We examined the correlation between SIRPγ and YAP expression and their prognostic value in our cohort of 182 LUAD patients. We found that concomitant high expression of SIRPγ and YAP occurred in 89 cases (48.9%) and was significantly associated with clinicopathologic features (Figure 9A and Supplemental Tables 2 and 5). Most importantly, Kaplan-Meier survival analysis revealed that the combination of elevated SIRPγ and YAP expression in the lung tumors significantly predicted poor survival outcome (Figure 9B). These findings underscore the clinical importance of the SIRPγ/YAP axis in LUAD.

### SIRPγ-neutralizing antibody targets both CSLCs and immune evasion to inhibit tumor growth in vivo.

**Figure 10:**
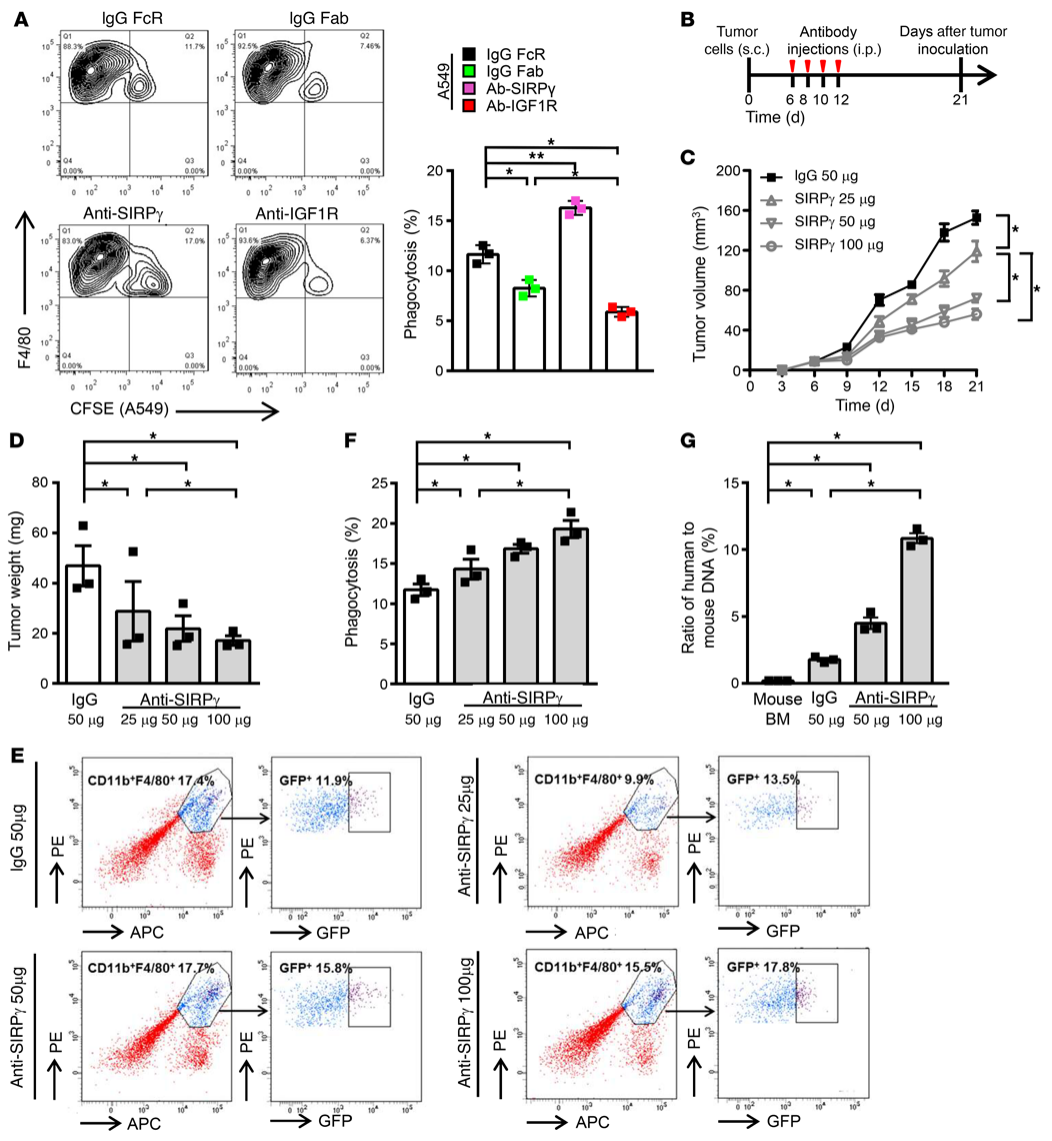
SIRPγ-neutralizing antibody targets both CSLCs and immune evasion to inhibit tumor growth in vitro and in vivo. (**A**) Phagocytosis of A549 cells with or without anti-SIRPγ LSB2.20 or anti–IGF-1R antibody treatment (4 μg/mL, 6 hours). (**B**) Strategy of LSB2.20 treatment of A549 tumor xenografts in female nude mice (*n* = 5 per group). (**C** and **D**) LSB2.20 inhibits A549 xenograft growth (**C**) and reduces the xenograft weight (**D**). (**E** and **F**) LSB2.20 promotes phagocytosis in A549 xenografts, represented by the enhanced percentage of GFP^+^F4/80^+^CD11b^+^ cells in total F4/80^+^CD11b^+^ cells. (**G**) The ratio of human DNA to mouse DNA in sorted tumor macrophages. All experiments were carried out at least in triplicate and the data are presented as the mean ± SD. *P < 0.05; **P < 0.01 by 1-way ANOVA.

Our study implies that SIRPγ could be a valuable target for therapy of LUAD with the potential to both directly attack tumor- and metastasis-initiating CSLCs and to inhibit an important mechanism of immune evasion. To obtain proof-of-principle evidence for the efficacy of SIRPγ targeting in LUAD, we utilized a well-characterized SIRPγ-specific mAb, LSB2.20, isolated by Piccio et al. (43), which recognized SIRPγ but not SIRPα (Supplemental Figure 2I). We found that incubation of A549 cells with this mAb in cell culture inactivated YAP signaling, as determined by enhanced phosphorylation of MST1, LATS1, and YAP, accompanied by reduced expression of YAP downstream targets, SOX2, IL-1β, GM-CSF, and CD47 (Supplemental Figure 13, A and B). As in the case of downmodulation by shRNA, expression of *CD47* in anti-SIRPγ mAb–treated tumor cells was restored by exposure to IL-1β and GM-CSF (Supplemental Figure 13C). In addition to antagonizing the effect of SIRPγ on signal transduction, treatment with the anti-SIRPγ mAb decreased stem cell sphere formation by A549 cells and enabled macrophage-mediated phagocytosis (Figure 10A, Supplemental Figure 13D, and Supplemental Figure 14, A–C).

We next investigated the efficacy of the neutralizing mAb in the xenograft model, utilizing *nu/nu* mice inoculated with GFP-labeled A549 cells (note that *nu/nu* mice, while lacking T cells, are known to retain a functional macrophage population). Beginning 6 days after tumor cell injection, the mice received 4 doses of the anti-SIRPγ mAb, administered every second day, and tumors were assessed on day 21 after inoculation (Figure 10B). We observed that the SIRPγ mAb treatment markedly inhibited tumor growth in vivo in a dose-dependent manner, as determined by reduced tumor size and weight (Figure 10, C and D). We further showed that treatment with the SIRPγ mAb significantly increased phagocytosis in the in vivo tumor model, as assessed by 2 distinct methods: (a) quantification of F4/80^+^CD11b^+^ macrophages containing GFP, indicative of having engulfed labeled A549 cells; and (b) the ratio of human-specific sequences (derived from tumor cells) to mouse-specific sequences in DNA extracted from macrophages isolated by flow cytometry from the tumor-bearing mice (Figure 10, E–G). We rule out the possibility that indirect FcR-mediated phagocytosis accounts for phagocytosis induction by the anti-SIRPγ mAb, as other anti-FcR antibodies did not similarly induce phagocytosis (Supplemental Figure 14, B and C). Using rescue experiments, we showed that restoration of CD47 compromised the increase in phagocytosis upon SIRPγ targeting by its neutralizing mAb, leading to rescue of the defect in tumorigenesis (Supplemental Figure 14, D–H) and supporting the notion that SIRPγ acts through CD47-dependent phagocytosis suppression to promote tumorigenesis.

**Figure 11:**
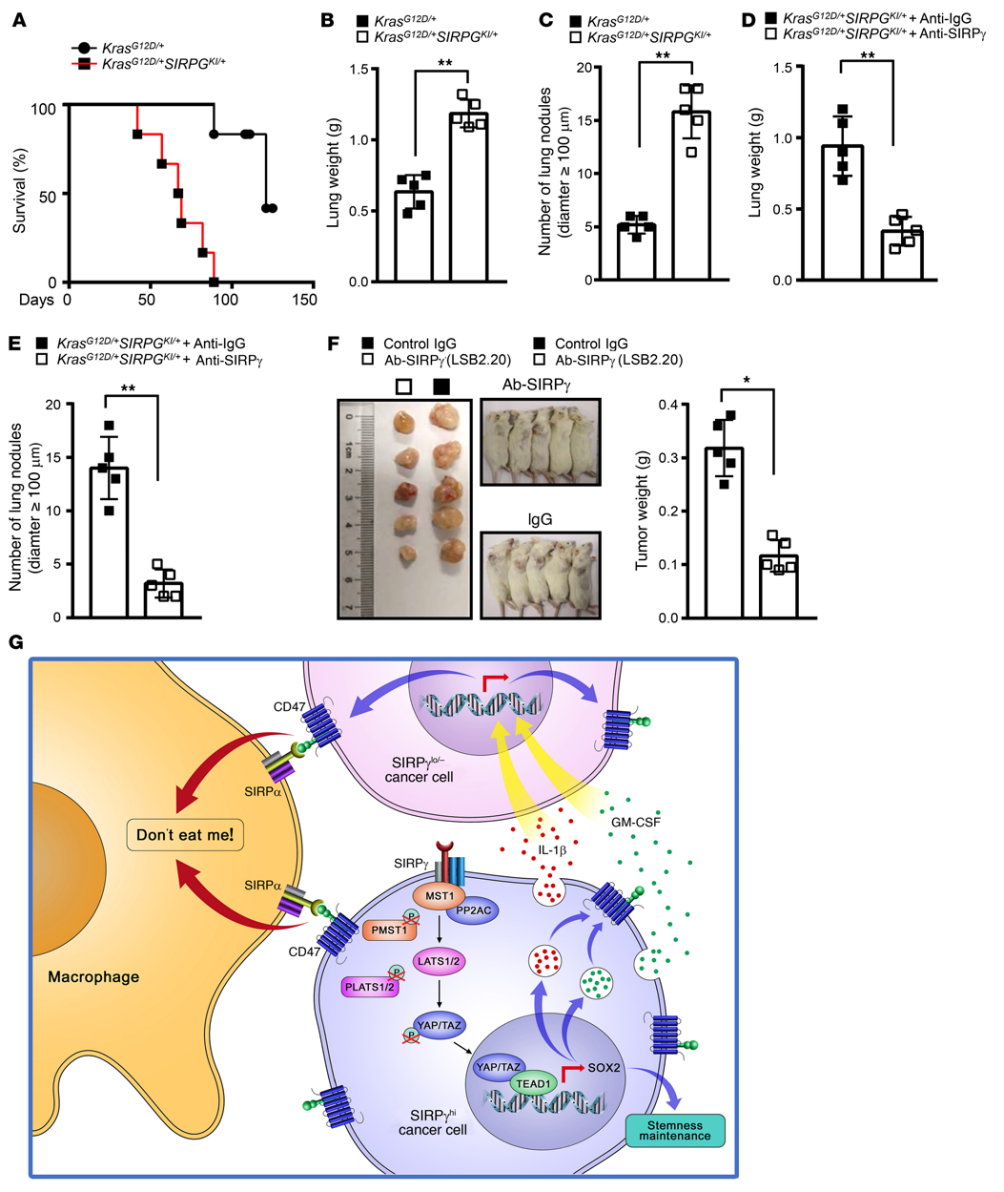
SIRPγ-neutralizing antibody inhibits tumor growth in vivo. (**A**) The overall survival of *Kras^LSL-G12D/+^SIRPG^KI/+^* (*n* = 5) and *Kras^LSL-G12D/+^* mice (*n* = 5). (**B**) Lung weight of indicated groups. (**C**) The number of metastatic lung nodules of indicated groups. (**D**) Lung weight of indicated groups. (**E**) The number of metastatic lung nodules of indicated groups. (**F**) LSB2.20 inhibits in vivo growth of lung adenocarcinoma PDX and reduces the xenograft weight. (**G**) SIRPγ^hi^ tumor cells sustain CD47 expression in bulk cancer cells to escape from macrophage-mediated phagocytosis through a paracrine-dependent manner by promoting YAP-dependent cytokine release. All experiments were carried out at least in triplicate and the data are presented as the mean ± SD. *P < 0.05; **P < 0.01 by log-rank test for survival (**A**) or unpaired, 2-tailed Student’s *t* test (**B**–**F**).

SIRPγ is highly expressed in T lymphocytes and activated NK cells (13). However, the function of SIRPγ in T cells and its underlying mechanisms are not well understood. A previous study showed that targeting SIRPγ by its neutralizing antibody (LSB2.20) blocks T cell transendothelial migration (37), raising a concern about using SIRPγ targeting for treating patients with NSCLC. However, we found that SIRPγ targeting by LSB2.20 did not obviously affect transendothelial migration of primary human T cells (Supplemental Figure 14I). We noticed that the previous study used a much higher dose of SIRPγ antibody (20 μg/mL) than in our study (4 μg/mL), which may have caused the discrepancy. Moreover, we found that targeting SIRPγ did not impair human primary T cell proliferation and activation (Supplemental Figure 14, J and K). We would like to point out that targeting SIRPγ with either 2 or 4 μg/mL LSB2.20 could markedly inhibit YAP signaling, cancer sphere formation, and elicit increased phagocytosis (Figure 10A and Supplemental Figure 13, A and D). Thus, our study suggests that targeting SIRPγ would not likely cause a concern for dampening T cell immunity. To further address this concern, we generated a human *SIRPG*-knockin mouse model by CRISPR/Cas9-mediated homology-directed repair and studied the role of SIRPγ and its targeting in a *Kras^LSL-G12D/+^* lung adenoma model with intact immunity (Supplemental Figure 15, A and B). *SIRPG* knockin enhanced the lung adenoma growth in vivo induced by the *Kras^LSL-G12D/+^* mutation (Figure 11, A–C, and Supplemental Figure 15C). In *Kras^LSL-G12D/+^SIRPG^KI/+^* compound mice treated with SIRPγ-blocking antibody, we found that targeting SIRPγ by LSB2.20 reduced the in vivo tumor growth of lung adenoma compared with the anti-IgG–treated group (Figure 11, D and E).

We also generated humanized NDG mice by injecting human PBMCs into NDG mice via the tail vein. Flow cytometric analysis showed that CD45^+^, CD4^+^, and CD8^+^ lymphocytes were in the blood of mice (Supplemental Figure 16, A–D). When equal numbers of A549 cells (1 × 10^6^) were inoculated into NDG mice reconstituted with or without PBMCs for tumorigenesis assays, targeting SIRPγ with LSB2.20 resulted in better efficacy in suppressing tumor growth in humanized NDG mice than in NDG mice without PBMC reconstitution (Supplemental Figure 16, E–G). It is important to note that many CD4^+^, CD8^+^, and CD68^+^ cells were infiltrated into tumor tissues from humanized NDG mice (Supplemental Figure 16H). Moreover, SIRPγ-overexpressing Lewis lung carcinoma cells were injected into C57BL/6 mice via the tail vein. The results showed that the anti-SIRPγ blocking antibody decreased tumor growth compared with anti-IgG treatment (Supplemental Figure 16, I–N). Treatment with the SIRPγ mAb significantly increased phagocytosis in C57BL/6 mice (Supplemental Figure 16O). Importantly, we showed that targeting SIRPγ with LSB2.20 also reduced in vivo tumor growth of a LUAD patient–derived xenograft (PDX) model (Figure 11F). Taken together, our data using xenografts, PDX models, and syngeneic and genetic mouse models with intact immunity provide the important proof of principle that targeting SIRPγ is a promising strategy for NSCLC treatment.

## Discussion

SIRPγ was previously shown to be expressed primarily in T cells and NK cells (13), but its expression and functional roles in cancer cells have never been reported to the best of our knowledge. Our study reveals the expression and functional role of SIRPγ in cancer cells. We show that SIRPγ is upregulated in patients with NSCLC, and its overexpression predicts poor survival outcome, highlighting the potential role of SIRPγ in NSCLC progression. Importantly, we showed that SIRPγ not only serves as a CSLC marker of NSCLC by using numerous in vitro and in vivo approaches, but also plays a key role in maintaining CSLCs of NSCLC, in turn promoting cancer progression and metastasis of NSCLC in animal models. Thus, our study not only opens up a promising avenue for studying SIRPγ in cancer, but also offers a potential target for NSCLC treatment.

Targeting CSLCs represents a promising strategy for cancer therapy. However, there is thus far no effective strategy to eliminate CSLCs. Identification of a unique transmembrane protein playing a key role in CSLC maintenance would offer a promising strategy to target CSLCs. We demonstrate in this study that SIRPγ^hi^ lung tumor cells represent unique CSLCs, which are critical for tumorigenesis and metastasis through activating YAP signaling, and that targeting SIRPγ by genetic and pharmacological approaches markedly inhibits lung tumor growth in xenograft, genetic, and PDX models, as well as growth of cancer organoids. Thus, SIRPγ targeting represents a promising strategy for CSLC and lung cancer targeting. Importantly, we also showed that SIRPγ is enriched in spheres of diverse cancer cell lines other than those derived from lung cancer and that its targeting also suppresses sphere formation and YAP signaling in a liver cancer cell line, suggesting that targeting SIRPγ may serve as a strategy for cancer other than lung cancer.

Hippo/YAP signaling is identified as one of the key pathways that plays a critical role in cancer initiation, progression, and metastasis. Although the Hippo kinase MST1/LATS1 is activated by adaptor proteins such as Merlin and Scribble in response to extracellular signals (29), little is known about upstream signals and regulators that may inhibit the MST1/LATS1 axis to induce YAP signaling activation. Our study identifies SIRPγ as an upstream regulator and/or signal to shut off MST1/LATS1 kinase activation. SIRPγ achieves this activity by serving as a scaffold to bridge MST1 and PP2A, thereby enabling PP2A to induce MST1 dephosphorylation and its subsequent inactivation. Inactivation of MST1/LATS1 kinase signaling by SIRPγ maintains YAP in a hypophosphorylated state, thereby facilitating YAP nuclear translocation and expression of YAP’s target genes. Our study therefore highlights the critical role of SIRPγ in YAP signaling activation that leads to promoting cancer progression and metastasis. Thus, targeting SIRPγ not only represents an effective strategy to abrogate YAP-dependent signaling, but also serves as a CSLC- and immune-targeting strategy to block cancer progression and metastasis.

Intriguingly, recent studies have connected Hippo/YAP signaling to cancer immune response and inflammation (44–46). Genetic deletion of Hippo kinases MST1 and MST2 in hepatocytes led to hepatic cellular carcinoma by promoting YAP-dependent MCP1 expression and massive infiltration of macrophages (47). Furthermore, the Hippo pathway effector TAZ also elicits liver inflammation to promote liver cancer development (48). As the SIRPγ/YAP axis empowers NSCLC cells to escape from phagocytosis, we speculate that SIRPγ may act through YAP-dependent inflammation to promote macrophage infiltration and/or M2 macrophage conversion, leading to immune escape and tumorigenesis. Future studies are warranted to further test this hypothesis.

The expression of CD47 in cancer cells enables cancer cells to initiate a “don’t eat me” signal for their escape from phagocytosis by macrophages. However, how CD47 expression in cancer cells is regulated has not been well understood. Our study reveals that SIRPγ enriched in CSLCs is a key mediator to maintain CD47 expression both in CSLCs and bulk cancer cells by inducing expression and secretion of IL-1β and GM-CSF from CSLCs in a YAP-dependent manner, offering mechanistic insight into how CD47 expression is orchestrated during cancer progression. Importantly, adding back IL-1β and GM-CSF to SIRPγ-deficient cancer cells rescued CD47 expression, leading to inhibition of phagocytosis by macrophages and restoration of tumorigenesis. Our study uncovers that YAP-dependent IL-1β and GM-CSF cytokine release induced by SIRPγ serves as a key mechanism to maintain CD47 expression, leading to the inhibition of phagocytosis and promotion of tumorigenesis.

Although cancer cells generally acquire immune escape capability, how they receive such an immune evasion signal remains largely unclear. It is postulated that a small subset of cancer cell populations with CSLC properties may transmit such a signal to bulk cancer cells, but the identity of this small cell population and the underlying mechanism are puzzling. Our findings provide the critical answers to these long-standing puzzles. Specifically, we identify a SIRPγ^hi^ cell population as a small subset with CSLC properties that transmits the immune escape signal through sustaining CD47 expression in CSLCs and bulk cancer cells, empowering them to escape from macrophage-mediated phagocytosis and leading to tumorigenesis. This action of SIRPγ^hi^ cells to enhance expression of an immune checkpoint in the tumor as a whole is achieved through autocrine/paracrine-dependent signaling via cytokines, such as IL-1β and GM-CSF, regulated by YAP (Figure 11G). Hence, targeting SIRPγ represents a potential therapeutic strategy to inhibit YAP signaling activation, thereby both attacking CSLCs and preventing an important mode of immune escape.

In summary, our study identifies SIRPγ, previously considered a protein with restricted expression and function in the immune system, as a putative CSLC marker in human LUAD, and potentially many other cancers, that exerts a potent regulatory influence on the critical Hippo/YAP signaling system, providing the first molecular mechanism by which SIRPγ is engaged. The effects of SIRPγ on both promoting the intrinsic properties of CSLCs and on the capacity of bulk tumor cells to evade innate immune surveillance imply that the protein could be a significant therapeutic target. This innovative concept receives support from in vitro and in vivo studies with genetic and pharmacological mAb approaches in xenograft models, cancer organoids, genetic models, and PDX models.

## Methods

See the Supplemental Methods for a detailed description of all experimental procedures. The microarray sequencing data are available in the NCBI’s Gene Expression Omnibus GEO database (GEO GSE192790).

### Study approval.

The study was approved by the Medical Ethics Committee of the Sun Yat-Sen University Cancer Center and Third Military Medical University. For in vivo tumor experiments, all procedures were approved by the Institutional Animal Care and Use Committees of Wake Forest School of Medicine, Third Military University, and Sun Yat-Sen University Cancer Center.

## Author contributions

CX, GJ, HW, WC, and HZ performed the experiments. GW, WZ, XZ, FH, ZC, BSP, Y Liu, AZ, JL, YW, RKM, CCH, SW, XM, JD, WL, BW, GX, HL, ZC, ZX, MEF, QX, SEHM, Y Lu, and DX provided technical support, critical comments, and suggestions. MEF, CX, HW, and HKL edited the manuscript. CX, HW, and QX analyzed the data. CX, HW, GJ, XB, and HKL designed the experiments, analyzed the data, and wrote the manuscript.

## Supplementary Material

Supplemental data

## Version 1 03/01/2022

Electronic publication
