# Supplementary material for: SIRPγ-expressing cancer stem-like cells promote immune escape of lung cancer via Hippo signaling
Source: J Clin Invest. 2022 Mar 1;132(5):e141797. doi: 10.1172/JCI141797 (PMC8884909; doi:10.1172/JCI141797)
Supplement: Supplemental data [file jci-132-141797-s012.pdf]

Supplemental Materials

Methods

Patient samples. The tissues used in this study were collected from a total of 194 patients with primary lung adenocarcinoma (LUAD) who received surgical resection in Sun Yat-sen University, China and Third Military Medical University, China. All patients consented before surgery or any other clinically indicated procedure. Tumor grades were defined according to the criteria of the World Health Organization. The Tumor-Node-Metastasis (pTNM) status of all LUADs was assessed according to the criteria of the sixth edition of the TNM classification of the International Union Against Cancer. The clinicopathological characteristics of the fresh tissues used in immunoblotting assay were summarized in Supplemental Table 1, and clinicopathological characteristics of patients used in immunohistochemical staining assay were summarized in Supplemental Table 2.

Cell lines and cell culture. Human embryonic kidney cell line 293T, murine fibroblast cell line L-929 (CCL-1™), murine lewis cells (LLCs) and human lung carcinoma A549 and H1975 cells were obtained from the American Type Culture Collection (ATCC, Rockville, MD, USA). 293T, A549 and H1975 cells were cultured in Dulbecco's modified Eagle's medium (DMEM) containing 10% fetal bovine serum (FBS) in a humidified 37°C incubator with 5% CO_2 atmosphere. The resources of the other cancer cell lines used in the study were shown in Supplemental Table 6. Cancer cells were infected with lentiviruses which were generated with a pCDH-CMV-MCS-EF1 lentiviral vector expressing a luciferase–eGFP fusion protein and were sorted by flow cytometry with a BD FACSAria II cell sorter to establish GFP^+ A549 cells. L-929 cells were cultured until confluent and L-929 conditioned medium was harvested at confluence and then used as a source of M-CSF for culture of bone marrow derived macrophages (1). Tumor sphere culture was performed as described previously (2). Briefly, A549 and H1975 cells were grown in serum-free medium (SFM) composed of DMEM/F12 (Gibco, USA), basic fibroblast growth factor (bFGF, 20 ng/ml; Upstate, USA), epidermal growth factor (EGF, 20 ng/ml; Sigma-Aldrich, USA), and B27 supplement (20 µl/ml; Life Technologies, USA). Reagents and antibodies used in this study were listed in Supplemental Table 7.

Virus production. Full-length human *SIRPG*, *YAP* and *CD47* in the pLVX-CMV-EGFP-3FLAG-PGK-Puro vector were purchased from SunBio (Shanghai. China) for the overexpression assay. pMAG ic3.1.CMV.NeoR.U6.shRNA vector and pMAGic2.1.CMV.HygroR.U6.shRNA vector was purchased from SunBio (Shanghai, China) for the knockdown assay. The sequences of the short hairpin RNA (shRNA) are listed in Supplemental Table 8. For producing virus stocks, the human HEK293T (obtained from the American Type Culture Collection) was kept in high-glucose DMEM containing 10% FBS, 2 Mm glutamine, and 100 units/ml penicillin and streptomycin. Nearly confluent cells in six-well plates were transfected with 4 μg of retroviral vector by using Lipofectamine 2000 (Invitrogen, USA) according to the manufacturer's protocol. Batches of virus containing supernatant were collected at 24h and 48h. The supernatant was passed through a 0.45μm syringe filter and frozen in liquid nitrogen. Where indicated, the virus was concentrated immediately before infection as described.

Target cell transduction. Cancer cells used in this research were cultured in DMEM with 10% FBS, 2mM glutamine and 100 units/ml penicillin and streptomycin. For infection,$1.5*10^5$ cells were mixed with 450μl of virus containing supernatant in the presence of 4 μg/ml polybrene (Sigma, USA), seeded into a well of a 6-well plate, and incubated at 37°C. Twelve hours post infection, the medium was changed to normal culture medium, 48 h post infection, cells were trypsinized and seeded into a well of a 10cm dish in the presence of 4 μg/ml puromycin or 800 μg/ml hygromycin (BD Biosciences, USA). Selection was done for 48h or 60h, respectively. Six days post infection, the overexpression or knockdown efficiency was detected by qRT-PCR and immunoblotting. For double knockdowns, cells were sequentially infected with pLVX-puro virus, selected with puromycin, infected with pMAG ic3.1-NeoR virus, selected with neomycin, infected with pMAGic2.1-Hygro virus, selected with hygromycin as above.

cDNA microarray and data analysis. was performed and genes were determined to be differentially expressed when logarithmic gene expression ratio was more than 2-fold different, and the *p*-values were less than 0.05. For data validation, mRNA levels of the interested genes were analyzed by quantitative RT-PCR. All target genes and primer sequences used for validation were provided in supplementary table 9. To examine genes that might be systemically altered, both Kyoto Encyclopaedia of

Genes and Genomes (KEGG) Pathways Analysis and Gene Set Enrichment Analysis (GSEA) were used for pathway analysis.

Flow cytometry analysis. Flow cytometry was performed on a FACS Calibur (BD Biosciences, USA). Cancer cells or tumor xenograft cells were dissociated into single-cell suspensions, washed and incubated in staining solution containing 1% BSA and 2 mM EDTA with the fluorescent monoclonal antibodies or respective isotype controls at 4 °C for 30 min. Antibodies were conjugated directly with FITC, PE or if not available, fluorophore-conjugated secondary antibodies were used. SYTOX Blue was used to exclude dead cells. The cells were then analyzed and results were calculated using Cell Quest software (BD Biosciences, USA).

Depletion of macrophages. Mice were injected intravenously with liposomal clodronate or liposomal vehicle (200 µl per mice; Yeasen, Shanghai, *n*=6 per group) as described previously (3). The effect of macrophage depletion was analyzed by flow cytometry analysis.

Cell proliferation assay. Cancer cells were seeded in 96-well plates at 2×10^3 cells per well in 0.2 ml DMEM medium containing 10% FBS. The cells were incubated at 37°C and 5% CO_2 for 8 days. In the following 8 days. The MTT assay was performed daily. MTT [3-(4,5-dimethylthiazol-2-yl)-2,5-diphenyltetrazolium bromide] solution (50 µl, 2 mg/mL, Sigma-Aldrich, USA) was added to cell culture, followed by incubation at 37°C for 4 h. Culture medium was replaced by 150 µl DMSO and optical density was measured at 570 nm with an automated BioTek Microplate Reader and Spectrophotometers (Bio-Rad, USA).

Self-renewal assay. Adherent monolayer cancer cells were dissociated into single-cell suspension. Equal number of cells was seeded in a 96-well plate. Cells were cultured in stem cell media to obtain primary spheres and second-generation spheres. Floating spheres and the total cell numbers were counted under light microscopy.

Co-culture assay. The Millicell Cell Culture Insert (0.4µm, sigma, USA) was used for the co-culture assay, 5×10^4 empty vector or SIRPG overexpressing A549 and H1975 cells were placed in upper chamber and equal number of empty vector-expressing A549 and H1975 cells were placed in the lower chamber in a time-dependent manner.

At the different time points, cells in the lower chamber were harvested for the Immunoblotting analysis.

Phagocytosis assay in vitro. FACS-based phagocytosis assays were performed to evaluate the phagocytic abilities of macrophages. Bone marrow derived macrophages (BMDM) were harvested, and immediately plated in petri dishes in DMEM supplemented 20% L-929-conditioned medium. Cells were fed on day 2 and day 5. 7 days later cells were stimulated by LPS 200 ng/ml together with IFNγ 40 ng/ml for 48 hrs to induce M1 type macrophages. THP1 were treated with PMA 100ng/ml for 48 hrs, then stimulated by IFN-gamma (20ng/ml) and LPS (240ng/ml) for 48 hrs to induce human M1 type macrophages. The induced M1 macrophages were then cultured in serum-free DMEM for 12 hrs and trypsinized, 2×10^4 M1 macrophages and an equal number of target cancer cells were added into the FACS tube in a total volume of 200 μl. All tubes were incubated at 37°C for 2 h to 24 hrs under the indicated conditions. After co-incubation, cells were incubated for 30 mins with PE or PE cy7-conjugated anti-mouse F4/80 antibody to stain macrophages. Cells were washed for 3 times with serum-free DMEM in 200 μl of serum-free DMEM. Phagocytosis was assessed by flow cytometry (BD FACS) using an enhanced green fluorescent protein (GFP) or PE or PE-cy7 filter set. At least 10000 macrophages were counted per tube. The phagocytic index based on FACS was calculated as the number of $F4/80^+GFP^+$ or $PKH26^+GFP^+$ cells divided by the total number of $F4/80^+$ cells. In each experiment, phagocytic indices were normalized to the maximal indexes. For an alternative flow cytometry-based assay, cancer cells were incubated with 2.5μM of carboxyfluorescein succinimidyl ester (CFSE) using a CFSE Cell Proliferation Kit (C34554; Life Technologies, Burlington, Ontario, Canada) and then cocultured with macrophages treated by M-CSF (50 ng/ml, Sigma-Aldrich, USA) for 7 days in the FACS tubes. Once the phagocytosis period was completed, cells were then stained on ice for 30 min with APC or PE-cy7 conjugated anti-mouse monoclonal antibody F4/80, and analyzed by flow cytometry. Phagocytosis efficiency was determined as the percentage of $F4/80^+CFSE^+$cells in total $F4/80^+$ macrophage cells. For the microscopy-based assay, bone marrow derived macrophages were treated by M-CSF for 7 days and THP1 were induced to M1 macrophage as mentioned above. Macrophages were labeled with 20μM of PKH26 (Sigma-Aldrich, USA) according to the manufacturer's protocol and 2×10^4 macrophages were seeded overnight in a 24-well tissue culture plate. The next day, target cells were washed and

labelled with 2.5μM of CFSE. After incubating macrophages in serum-free medium for 2h, 2×10^4 CFSE-labelled cancer cells were added to the macrophages and incubated at 37 °C. Macrophages were extensively washed and imaged with a Fluorescence microscope 2 hours later (Carl Zeiss Axiovert S100 TV). The phagocytosis efficiency was calculated as the number of macrophages containing $CFSE^+$ cancer cells per 100 macrophages.

In vivo phagocytosis assays. BALB/c athymic female nude mice (6 weeks old) were randomly assigned into four groups (n=5/group). 1×10^6 A549 cells of indicated groups (shControl&Vector, shSIRPG&Vector, shControl&Ov-YAP1 and shSIRPG&Ov-Y AP1) were inoculated subcutaneously in the right flank. 21 days later, mice were sacrificed, tumor xenografts were isolated, and tumor volume (TV) and tumor weight were measured. Tumor volume (TV) was calculated using the following formula: TV (mm3) = d2×D/2, where d and D represent the shortest and the longest diameters. Tumor tissues were harvested in 1X PBS, minced and digested into a single cell suspension in a mixed solution containing collagenase type 1 and type 4 (1.5 mg/ml) in DMEM for 1.5hrs at 37°C. The suspension was filtered through a 70μm-cell strainer, and cells were washed 3 times with DMEM and resuspended in cold flow buffer. Single-cell suspensions were incubated with APC labeled rat anti-mouse CD11b and PE-cy7-labeled rat anti-mouse F4/80 at 4°C for 30 mins, washed and re-suspended in cold flow buffer. Flow cytometric data were obtained using a FACS Calibur (BD Biosciences, USA) and analyzed with FlowJo software. For the other set of phagocytosis assay in vivo, 1×10^6 A549 cells (n=5/group) were inoculated subcutaneously in the right flank. Six days after inoculation, the mice were treated with an intraperitoneal injection of control mouse IgG or the SIRPG monoclonal LSB2.20 every 2 days for a total of 4 injections (IgG 50μg per injection; LSB2.20 at a dose of 25μg, 50μg or 100μg per injection). 21 days later, mice were sacrificed and the phagocytosis assay was conducted as details described above. In addition, total DNA was extracted from sorted macrophage cells using TIANamp Genomic DNA Kit (TIANGEN, China). Mouse DNA was amplified as an internal control, and the ratio of human to mouse DNA in sorted mouse xenograft macrophages was determined by qRT-PCR. For the other set of phagocytosis assay in vivo A549 control and shSIRPG cells were injected subcutaneously to six-week-old male nude mice (1×10^6 cells per mouse; *n*=5 mice per group). Six days after inoculation, the mice were treated with an intraperitoneal injection of control mouse

IL-1β or GM-CSF every 2 days for a total of 4 injections (IL1β 100 μg per injection; GM-CSF at 100 μg per injection). 5 weeks later, mice were sacrificed, and then analyzed the phagocytosis as details described above. And for another set of phagocytosis assay in vivo, A549 vector and ov-CD47 cells were diluted in serum-free DMEM medium and injected subcutaneously to six-week-old male nude mice (1×10^6 cells per mouse; *n*=5 mice per group). Six days after inoculation, the mice were treated with an intraperitoneal injection of control mouse IgG or the SIRPG monoclonal LSB2.20 every 2 days for a total of 4 injections (IgG 50 μg per injection; LSB2.20 at a dose of 50 μg per injection). 5 weeks later, mice were sacrificed and analyzed the phagocytosis as details described before.

In vivo tumorigenesis and metastasis assays. A549 cells with stable expression of firefly luciferase (Xenogen) were infected with lentiviruses carrying empty vector, ovSIRPG, sh-Control, sh-SIRPG, or sh-SIRPG&ovYAP1 expression construct. Cancer cells were mixed with Matrigel (1:1) and inoculated subcutaneously (1×10^6 cells per mouse, *n*=5 mice per group) or injected into the lateral tail vein (1×10^5 cells per mouse, *n*=6 mice per group) of 6-week-old immunodeficient female nude mice (*n*=6 per group). $SIRPG^{high}$ or $SIRPG^{low/-}$ cells were sorted from A549 monolayer cells by flow cytometry. The serial dilutions of viable cells with equal number (1×10^2; 1×10^3; 1×10^4; 5×10^4; 1×10^5 and 5×10^5) were diluted in serum-free DMEM medium and injected subcutaneously to four-week-old male nude mice (n=6 for each group, Center of Experimental Animals, Third Military Medical University, Chongqing, China). Lewis cells were infected with lentiviruses carrying empty vector, ovSIRPG expression construct. Cancer cells were inoculated subcutaneously (1×10^6 cells per mouse, *n*=6 mice per group) or injected into the lateral tail vein (1×10^5 cells per mouse, *n*=6 mice per group) of 6-week-old immunodeficient female C57 mice (*n*=6 per group). Mice were monitored every week for the appearance of subcutaneous tumors. At the end of 6 weeks, mice were sacrificed, and tumor xenografts were removed to measure tumor volume (TV) and tumor weight. Tumor volume (TV) was calculated using the following formula: TV (mm^3) = $d^2\times D/2$, where d and D represent the shortest and the longest diameters.

Generation of SIRPG cas9-ki mice. *$SIRPG^{KI/+}$* mice were generated by GemPharmatech Co., Ltd (Nanjing, Jiangsu Province) through CRISPR/Cas9 mediated homology-directed repair. Firstly, one gRNA-targeting the near sequence of

inserted site constructed and transcribed in vitro. And the donor vector with the inserted fragment was designed and constructed in vitro. Then, Cas9 mRNA, gRNA and donor will be co-injected into zygotes. Thereafter, the zygotes were transferred into the oviduct of pseudopregnant ICR females at 0.5 dpc. And F0 mice were born after 19~21 days of transplantation, all the offsprings of ICR females (F0 mice) were identified by PCR and sequencing of tail DNA. Finally, breeding positive F0 mice with wild-type mice to build up heterozygous mice. Genotypes of Cas9 mice were determined from purified mouse tail DNA.

Generation of KRAS$^{G12D/+}$SIRPG$^{KI/-}$ mice for lung tumorigenesis assay. The *B6.129S4-Kras^{tm4Tyj}/NJU* mice were donated from Sichuan University. *KRAS* $^{LSL\text{-}G12D/+}$ mice were crossed with 8 weeks old LSL-*SIRPG* $^{KI/-}$ mice to generate *KRAS* $^{LSL\text{-}G12D/+}$*SIRPG* $^{KI/-}$mice. Genotypes were determined by PCR. Only littermate mice were used in all experiments. 8-week-old male and female *KRAS* $^{LSL\text{-}G12D/+}$ and *KRAS* $^{LSL\text{-}G12D/+}$*SIRPG* $^{KI/-}$mice were intranasally injected with Ad-Cre and treated with a neutralizing antibody (IgG 50 μg per injection; LSB2.20 at a dose of 50 μg per injection) every other day for a total of 4 injections, followed by H&E staining of lung tissues collected from mice for lung tumorigenesis assay.

hPBMC reconstructed NDG mice. NOD.CB17-PrkdcscidIl2rgtm1Il15tm1(IL15)/Bcgen (NDG) mice were purchased from GemPharmatech Co., Ltd. Human peripheral blood mononuclear cells (hPBMC) were extracted by ficoll density gradient centrifugation from the peripheral blood of healthy blood donors. Then, 5×10^6 human PBMCs were injected intravenously into each NDG mice. 1 weeks later, we detected the proportion of human CD45+, CD8+, CD4+ immune cells in the mouse peripheral blood by flow cytometry analysis. After 2 weeks post-implantation, 1×10^6 A549 cells in 100ul PBS were inoculated subcutaneously into mice to establish subcutaneous transplantation tumor models of humanized mice. 1 week after transplantation, mice were intraperitoneally injected with blocking antibody (IgG 50 μg per injection; LSB2.20 at a dose of 50 μg per injection) every other day for a total of 4 injections. 3 weeks after transplantation, transplanted tumors of mice were collected to detect tumor size and immune cell infiltration status.

Patient-derived ex vivo organoid model. *Ex vivo* organoids from resected human non-small cell lung carcinoma samples were generated and expanded as described

previously, with minor adaptations (3-5) Dissociated cell clusters (5×10^5 cells in total) were spun down and resuspended in 60% Matrigel (Corning)/organoid culture media and plated in the middle of one well of a pre-coated 24-well plate (Corning) with 60% Matrigel. All donors provided written informed consent, and the experiments were approved by Institutional Review Board of Sun Yat-Sen University Cancer Center (SYSUCC). Cell viability assay was performed 48-72 hours after infection of lentivirus carrying shRNA or overexpressing constructs via Cell Titer-Glo 3D Viability Assay (Promega, USA) luminescence on a Glomax microplate reader (Promega, USA).

Patient-derived xenografts (PDX). All procedures involving mouse were approved by the Institutional Animal Care and Use of SYSUCC. All mice were obtained from Nanjing Biomedical Research Institute of Nanjing University (NBRI). For LUAD PDX model (6), each sample was cut into approximately 2.5 mm × 2.5 mm × 2.5 mm pieces. Fragments were coated in Matrigel basement membrane matrix (Corning) and implanted in subcutaneous pockets in the posterior flanks of 6-week-old NOD-Prkdc^{em26Cd52}Il2rg^{em26Cd22}/Nju (NCG) mice (NBRI, Nanjing, China). Treatment was started after engrafted tumors grew to a median tumor size of approximately 50 mm^3. Five mice were included in each treatment group for each PDX tumor line. Each group of mice were treated with an intraperitoneal injection of control mouse IgG or the SIRPG mAb LSB2.20 every 2 days for a total of 4 injections (IgG 50 µg per injection; LSB2.20 50 µg per injection). The first day of treatment was designated as day 0 and treatment were continued for 4 weeks. Mice were sacrificed before tumors reached a critical size at the ethical end point (V = 1,000 - 4,000 mm^3). Tumor xenografts were removed, and tumor volume (TV) and tumor weight were measured. End-of-treatment tumor tissues were fixed in 10% formalin-fixed and FFPE tissue blocks were sectioned for HE staining.

Immunohistochemistry. Immunohistochemical staining of tumor xenografts was performed on sections using the streptavidin-biotin peroxidase complex (SABC) method as described previously[2] Briefly, xenograft samples were fixed in 4% paraformaldehyde at 4°C for 72 hrs and embedded in paraffin. The paraffin sections were prepared and incubated with primary antibodies at 4°C overnight. The slides were then reacted with biotinylated goat anti-mouse/rabbit IgG and avidin-biotin complex (ABC). For visualization of the antibody-antigen complex, chromogen

reaction was carried out with diaminobenzidine (DAB) and the slides were examined under a light microscope. For quantification, IPP software (image-pro plus 6.0) was used to analyze the optical density of the images within 5 random fields at 400× magnification. The average optical density (AOD), namely IOD/area, was calculated.

IHC staining intensity was independently scored by two anatomical pathologists. The staining intensity (negative=0, weak=1, moderate=2, or strong=3 scores) and the proportion of immunostaining positive cells of interest (<25%=1, 25 to 50%=2, >50% to <75%=3, >=75%=4) were scored. The immunostaining was semi-quantitatively categorized by combining the intensity and the quantity scores, which yield a staining index (values from 0 to 12). The staining index of 5-12 was regarded as high expression, while staining index of 0-4 was considered as low expression.

Immunofluorescence (IF) staining. For immunofluorescence staining, cells were attached to poly-L-lysine-coated coverslips in DMEM containing 10% FBS for 24 hrs and subsequently fixed in 4% paraformaldehyde for 20 mins. Cells were blocked with pre-immune goat serum at 37°C for 30 mins, and then incubated with primary antibodies at 4°C overnight. Antibody information used in the experiments will provided in supplementary table 7. The cells were subsequently washed in PBS and incubated at 37°C for 1 h with Cy3 or Cy5-conjugated goat anti-rabbit or anti-mouse IgG antibodies (1:1000; Invitrogen, USA). Nuclei were counterstained with Hochest 33258. Cells were observed under laser confocal scanning microscopy (Leica TCS-SP5, Germany). Additionally, freshly frozen human surgical biopsy specimens obtained from eight LUAD patients were used in immunofluorescence staining to detect protein expression. The immunofluorescence images were imported into Image-Pro Plus 6.0 software for further analysis.

Immunoblotting and immunoprecipitation. Cell lysates of cancer cells and tissue tumor cells were prepared using RIPA buffer with protease inhibitors and quantified using a BCA protein assay (Pierce, Rockford, IL). Proteins (20 μg) were loaded onto a 10% SDS- poly-acrylamide gel (SDS-PAGE) and transferred onto PVDF membranes (Millipore, USA). Membranes were incubated with TBS blocking buffer containing 2% milk and subsequently incubated with the membranes following specific primary antibodies: anti-SIRPγ (diluted 1:500; Santa cruz, catalog sc-53604 and sc-53112; diluted 1:1000; R&D, catalog MAB4486 and 595337), anti-GRP78 Bip (diluted 1:10000; Abcam, catalog ab32618), anti-CD47(diluted 1:1000; Abcam,

catalog ab3283), anti-YAP1 (diluted 1:1000; Abcam, catalog ab52771), anti-phospho-YAP1(S127) (diluted 1:1000; Abcam, catalog ab76252), anti-SOX2 (diluted 1:1000; Cell Signaling, catalog 3579), anti-MST1 (diluted 1:1000; Cell Signaling, catalog 3682), anti-phospho-MST1(Thr183)/MST2(Thr180) (diluted 1:1000; Cell Signaling, catalog 49332), anti-LATS1 (diluted 1:1000; Cell Signaling, catalog 3477), anti- phospho-LATS1(Ser909) (diluted 1:1000; Cell Signaling, catalog 9157), anti-Nanog (diluted 1:1000; Cell Signaling, catalog 4903), anti-POU5F1 (diluted 1:1000; Cell Signaling, catalog 2750), anti-β-Actin (diluted 1:1000; Cell Signaling, catalog 4970), anti-GAPDH (diluted 1:1000; Cell Signaling, catalog 5174), anti-PP2Ac (diluted 1:1000; Cell Signaling, catalog 2038), anti-IL-1β (diluted 1:1000; Abcam, catalog Ab226918), anti-CD44 (diluted 1:1000; Cell Signaling, catalog 3578), anti-CD133 (diluted 1:1000; Cell Signaling, catalog 64326), anti-SIRPα (diluted 1:1000; Santa cruz Sc-136067), anti-SIRPγ (diluted 1:1000; R&D 595337), anti-SIRPγ (diluted 1:1000; R&D MAB4486), anti-CD47 (diluted 1:1000; Abcam, catalog Ab108415), anti-GM-CSF (diluted 1:1000; Abcam, catalog Ab226918), anti-IL-1β (diluted 1:1000; Abcam, catalog Ab226918). Immunoreactive proteins were visualized using SuperSignal West Femto Trial Kit (Pierce, Rockford, IL) by an enhanced chemiluminescence detection system, followed by X-ray film exposure. The signals of proteins of interest were measured by densitometry. For immunoprecipitation, cell lysates were prepared and incubated with antibodies overnight, followed by incubation with 40 μl Protein A/G agarose beads for 2 hrs. The beads were then washed 4 times with lysis buffer and subjected to immunoblotting of specific antibodies. See the complete unedited blots in the Supplemental Figure 17.

Quantitative Real-time PCR (qRT-PCR). Total RNA was extracted from cancer cells using Trizol Reagent (Invitrogen, USA). qRT-PCR was performed using SYBR PrimeScript RT-PCR kit (TaKaRa, Japan) on a Rotor-Gene 6000 real-time genetic analyzer (Corbett Life Science, USA). Primers of cancer genes and the product sizes were listed in Supplemental Table 9 and 10. Glyceraldehyde-3-phosphate dehydrogenase (GAPDH) was amplified as an internal control. The PCR protocol included denaturation (95°C for 2 mins), followed by 40 cycles of amplification and quantification (95°C for 5 s, 55°C–57°C for 30 seconds) and melting curve (55°C–95°C, with 0.5°C increment each cycle). Each sample was tested at least in triplicates.

Statistical analysis. All experiments were performed in triplicate and repeated at least three times. Data were presented as the mean ± S.D. Statistical analysis was performed using SPSS software or GraphPad software. Differences between 2 groups were tested for statistically significant difference using unpaired, 2-tailed *Student's* t test or paired, 2-tailed *Student's* t test. Differences among 3 groups or more were tested using 1-way ANOVA test for univariate survival analysis, survival curves were obtained using the Kaplan-Meier method and comparisons were made using log rank test. Multivariate survival analysis was carried out on all parameters found to be significant in univariate analysis using Cox proportional hazards regression model. For analysis of contingency tables, Fisher's exact test was applied. Statistical difference was considered significant if *P*-values were <0.05.

Reference:

1. Zhang X, Goncalves R, and Mosser DM. The isolation and characterization of murine macrophages. *Current protocols in immunology.* 2008;Chapter 14:Unit 14.1.
2. Xu C, Xie D, Yu SC, Yang XJ, He LR, Yang J, et al. β-Catenin/POU5F1/SOX2 transcription factor complex mediates IGF-I receptor signaling and predicts poor prognosis in lung adenocarcinoma. *Cancer research.* 2013;73(10):3181-9.
3. Lu CH, Yeh DW, Lai CY, Liu YL, Huang LR, Lee AY, et al. USP17 mediates macrophage-promoted inflammation and stemness in lung cancer cells by regulating TRAF2/TRAF3 complex formation. *Oncogene.* 2018;37(49):6327-40.
4. Boj SF, Hwang CI, Baker LA, Chio, II, Engle DD, Corbo V, et al. Organoid models of human and mouse ductal pancreatic cancer. *Cell.* 2015;160(1-2):324-38.
5. Lee SH, Hu W, Matulay JT, Silva MV, Owczarek TB, Kim K, et al. Tumor Evolution and Drug Response in Patient-Derived Organoid Models of Bladder Cancer. *Cell.* 2018;173(2):515-28.e17.
6. Ruess DA, Heynen GJ, Ciecielski KJ, Ai J, Berninger A, Kabacaoglu D, et al. Mutant KRAS-driven cancers depend on PTPN11/SHP2 phosphatase. *Nat Med.* 2018;24(7):954-60.

Supplemental Table 1. Clinicopathological features of LUAD patients used in immunoblotting analysis.

Accession number	Gender	Age (years)	pT status	pN status	pM status	Stage
0000270544	M	65	3	0	0	IIA
0000273232	M	62	2a	N3	0	IIIB
0000273538	F	57	2a	N2	0	IIIA
0000275851	M	59	3	0	0	IIA
0000276279	F	76	2a	0	0	IB
0000277203	F	68	1c	0	0	IA
0000277802	M	71	2b	N2	0	IIIA
0000278221	M	64	2a	0	0	IB
0000271838	M	70	2a	0	0	IB
0000390679	M	57	1b	0	0	IA
0000399091	F	47	1b	0	0	IA
0000396470	M	67	1c	0	0	IA

Supplemental Table 2. Clinicopathological features and IHC staining index in 182 cases of LUAC patients.

Accession number	Gender	Age (years)	pT status	pN status	pM status	Tumor grade	Stage	Status	Survival time after diagnosis (month)	SIRPγ score	YAP score
201140086	M	69	2b	0	0	1	IIA	0	48	9	6
201108409	F	56	1c	0	0	2	IA3	0	55	4	2
201032093	F	64	2a	0	0	3	IB	0	61	0	1
201142460	F	61	1a	0	0	2	IA	0	47	4	3
201009243	M	51	1b	0	0	1	IA	0	67	2	1
201019300	M	70	1c	0	0	2	IA3	0	64	0	1
201142037	M	63	1c	0	0	2	IA3	0	48	1	2
201216662	F	65	1c	0	0	2	IA3	1	12	1	1
201117373	F	65	2a	0	0	2	IB	0	53	2	0
201128463	M	43	2a	0	0	3	IB	0	51	9	8
201130494	F	72	2a	0	0	2	IB	1	42	6	3
201230665	F	67	1c	2	0	2	IIIA	1	20	12	9
201035207	F	79	1c	2	0	2	IIIA	1	17	8	9
201234908	F	41	1c	2	0	1	IIIA	1	15	4	8
201012647	M	63	1a	2	1	3	IV	1	30	6	2
201003332	F	60	1a	0	0	2	IA	1	32	4	3
201140744	F	62	1b	0	0	2	IA	1	38	4	2
201222616	F	67	1b	0	0	2	IA	1	15	6	4
201116452	F	51	1c	0	0	2	IA3	0	53	4	0
201120743	F	72	1c	0	0	2	IA3	0	52	3	2

201133397	F	52	1c	0	0	2	IA3	0	50	9	1
201140740	M	78	1c	0	0	2	IA3	1	28	12	2
201143543	M	47	1c	0	0	2	IA3	1	12	8	2
201231053	M	46	2a	0	0	2	IB	1	24	0	2
201000582	M	68	2b	0	0	2	IIA	0	70	8	0
201007679	F	59	4	1	0	3	IIIA	1	17	4	9
201026462	F	52	2a	2	0	2	IIIA	0	62	0	2
201234942	M	45	2a	2	0	2	IIIA	1	27	1	2
201113395	F	81	1b	0	0	2	IA	0	54	8	1
201017997	F	63	1c	0	0	2	IA3	1	12	1	2
201023571	F	69	1c	0	0	2	IA3	1	57	4	12
201124516	F	65	1c	0	0	2	IA3	0	52	4	2
201136269	M	51	1b	1	0	2	IIA	0	49	6	2
201009976	M	68	1b	2	0	3	IIIA	1	11	8	12
201142038	M	47	3	2	0	2	IIIA	1	25	1	3
201215355	F	48	1a	1	0	2	IIA	1	21	6	4
201105101	F	65	1b	0	0	2	IA	0	56	2	6
201130295	M	75	1b	0	0	2	IA	0	50	2	6
201131132	F	58	1b	0	0	2	IA	0	50	4	3
201131935	F	64	1b	0	0	3	IA	1	5	4	9
201201660	M	54	1b	0	0	2	IA	1	40	4	9
201022090	M	71	1c	0	0	2	IA3	1	41	12	12
201036898	F	52	2b	0	0	2	IIA	0	59	8	3
201217470	F	70	2b	0	0	2	IIA	1	20	9	4
201221596	F	78	2b	0	0	2	IIA	1	12	6	6
201244292	M	44	1b	2	0	2	IIIA	1	15	9	3
201103810	M	43	1a	2	0	2	IIIA	1	12	12	6
201136289	M	68	1a	2	0	2	IIIA	1	33	4	9
201240589	F	58	2a	2	0	2	IIIA	1	35	0	0
201208528	F	48	4	0	0	3	IIIA	1	2	9	6
201115057	M	63	1c	3	0	3	IIIB	1	2	6	6
201228319	F	51	1c	2	0	2	IIIA	1	16	4	6
201144436	M	43	2a	2	0	2	IIIA	1	34	4	8
201107102	F	62	1b	1	0	2	IIA	0	55	8	6
201141630	F	66	1b	0	0	2	IA	0	48	2	1
201145516	F	64	1b	0	0	2	IA	0	47	8	3
201146900	M	40	1b	0	0	3	IA	1	18	1	1
201003467	F	70	1c	0	0	2	IA3	1	29	4	3
201109870	F	44	1c	0	0	2	IA3	0	55	9	3
201127587	F	75	1c	2	0	2	IIIA	1	28	9	3
201147554	F	52	2a	0	0	2	IB	0	46	9	6
201216993	F	61	2a	1	0	3	IIB	1	6	9	8
201009935	M	81	2b	0	0	3	IIA	1	18	4	9

201144384	M	60	2b	0	0	2	IIA	1	16	12	9
201229358	M	66	2a	1	0	2	IIB	1	15	6	4
201228122	F	69	2a	2	0	2	IIIA	1	20	4	8
201115523	F	61	2a	2	1a	2	IV	1	4	8	3
201139151	F	52	1c	0	0	2	IA3	0	48	3	0
201140764	F	66	2b	0	0	2	IIA	0	48	2	4
201209153	F	62	2b	0	0	2	IIA	1	18	9	3
201023763	F	56	1c	2	0	2	IIIA	0	63	4	3
201032926	F	77	1b	2	0	3	IIIA	0	60	3	0
201117800	F	63	4	0	0	3	IIIA	1	24	4	12
201240985	F	51	3	2	0	3	IIIA	1	15	9	8
201149658	M	62	1b	0	0	2	IA	1	41	6	6
201210905	F	75	1b	0	0	3	IA	1	31	12	8
201143740	F	64	2a	0	0	2	IB	1	14	8	3
201131715	F	53	2b	2	0	2	IIIA	1	16	8	3
201130137	M	69	1b	2	0	2	IIIA	1	10	6	0
201150556	M	48	2a	0	0	3	IB	1	32	4	6
201146731	M	47	2a	2	0	3	IIIA	1	27	9	6
201118761	F	46	1c	0	0	3	IA3	1	23	8	8
201136709	F	70	2a	0	0	2	IB	1	16	9	12
201143535	F	41	2b	0	0	2	IIA	0	47	6	6
201150771	F	75	2b	0	0	2	IIA	1	8	6	9
201036923	M	66	1b	0	0	2	IA	0	59	6	6
201004000	M	76	2a	0	0	1	IB	0	69	0	0
201010384	M	63	1b	1	0	1	IIA	0	66	2	0
201036138	M	63	1b	0	0	2	IA	0	59	0	1
201146307	M	60	1b	0	0	3	IA	0	47	8	12
201001192	M	65	1c	0	0	2	IA3	0	70	4	3
201004140	M	69	1c	0	0	2	IA3	1	6	9	0
201024963	F	67	1c	0	0	2	IA3	0	63	4	3
201040320	M	65	1b	2	0	2	IIIA	1	22	6	6
201019816	F	75	1c	2	0	1	IIIA	0	64	0	8
201114844	F	52	1b	0	0	2	IA	0	54	6	3
201208113	M	44	1c	0	0	2	IA3	1	32	12	9
201105495	M	65	1b	0	0	1	IA	0	56	6	0
201023244	M	60	1b	0	0	1	IA	0	63	9	8
201004305	M	68	1a	0	0	1	IA	0	69	8	9
201013478	F	52	1a	0	0	1	IA	0	66	6	4
201026124	F	46	1a	0	0	2	IA	0	62	2	2
201102493	F	74	1a	0	0	2	IA	0	57	3	1
201032729	M	61	1b	0	0	2	IA	0	60	6	6
201102465	M	59	1b	0	0	2	IA	0	57	8	6
201119431	F	69	1b	0	0	1	IA	0	53	8	0

201021415	F	53	1c	0	0	2	IA3	0	63	6	0
201039071	F	52	1c	0	0	2	IA3	1	47	1	4
201110409	M	58	1c	0	0	2	IA3	0	55	1	9
201116005	F	48	1c	0	0	2	IA6	1	24	3	1
201116498	F	70	1a	0	0	2	IA7	0	53	0	9
201117847	F	68	1c	0	0	1	IA3	0	53	9	1
201001712	F	53	2a	0	0	2	IB	1	11	6	0
201004396	M	66	2a	0	0	1	IB	0	69	4	2
201033746	F	51	2a	0	0	2	IB	0	60	6	2
201034512	F	62	2a	0	0	2	IB	1	37	8	8
201105321	F	74	2a	0	0	2	IB	0	56	4	9
201105861	M	68	2a	1	0	2	IIB	0	56	4	8
201134718	F	57	2a	0	0	1	IB	0	49	9	3
201024653	F	47	2b	0	0	1	IIA	0	63	4	0
201026584	F	70	3	0	0	2	IIB	0	62	8	1
201034467	F	60	1c	1	0	2	IIA	1	29	4	2
201103413	F	59	2a	1	0	3	IIB	0	57	3	4
201105279	F	75	1b	1	0	2	IIA	1	30	4	1
201002835	F	62	1b	2	0	1	IIA	1	32	4	9
201030896	F	49	2b	2	0	2	IIIA	0	61	8	1
201111176	M	76	1b	2	0	2	IIIA	1	32	8	9
201121180	M	78	1c	2	0	2	IIIA	1	32	6	9
RRsLug 0609A0201	M	60	2a	0	0	2	IB	1	62	2	1
RRsLug 0705A0317	F	57	4	2	0	3	IIIB	1	3	2	4
RRsLug 0612A0210	F	58	2a	0	1	2	IV	1	49	1	3
RRsLug 0706A0349	M	60	2a	0	0	2	IB	0	62	2	0
RRsLug 0411A0034	F	71	3	0	0	2	IIB	0	95	2	0
RRsLug 0801A0449	M	61	2b	3	0	2	IIIB	1	15	3	4
RRsLug 0701A0255	F	68	1	0	0	2	IA	0	67	2	0
RRsLug 0706A0343	M	59	2a	0	0	1	IB	0	62	0	2
RRsLug 0801A0448	F	60	3	3	0	1	IIIB	1	40	1	0
RRsLug 0411A0036	F	59	3	1	0	2	IIIA	1	38	1	3
RRsLug 0802A0471	M	63	3	2	0	2	IIIA	1	25	2	2

RRsLug 0905A0703	F	77	3	3	0	2	IIIB	1	29	0	1
RRsLug 0902A0640	F	53	2a	0	0	2	IB	0	43	3	0
RRsLug 0705A0321	F	52	3	3	0	3	IIIB	1	44	3	2
CRsLug 0612A0222	M	57	1	0	0	2	IA	0	72	2	2
RRsLug 0701A0252	F	50	1	0	0	2	IA	0	68	2	0
RRsLug 0710A0364	F	65	2a	0	0	2	IB	1	15	3	2
CRsLug 0612A0249	F	40	2a	0	0	2	IB	0	69	3	3
RRsLug 0802A0464	M	60	3	1	0	2	IIIA	0	55	3	2
RRsLug 0807A0546	F	73	4	2	0	2	IIIB	0	50	1	3
RRsLug 0811A0584	F	50	1	0	0	2	IA	1	17	3	1
RRsLug 0803A0482	M	61	2a	0	0	3	IB	1	39	2	0
RRsLug 0806A0523	M	64	2a	1	0	3	IIB	0	50	3	0
RRsLug 0612A0208	M	47	3	1	0	3	IIIA	1	33	1	4
CRsLug 0506A0108	F	50	2a	1	1	3	IV	0	86	3	3
RRsLug 0408A0013	F	84	1	0	0	1	IA	0	95	4	0
CRsLug 0609A0182	F	64	2a	0	0	1	IB	1	15	8	6
RRsLug 0807A0552	M	37	2a	0	0	3	IB	0	49	4	2
RRsLug 0803A0476	F	65	2a	1	0	2	IIB	0	54	8	3
RRsLug 0805A0519	M	65	2a	2	0	3	IIIA	0	51	8	1
RRsLug 0808A0556	M	61	1	0	0	1	IIB	0	48	4	3
RRsLug 0904A0682	F	65	2a	0	0	1	IIB	0	41	4	2
RRsLug 0804A0486	F	81	2a	1	0	2	IIB	0	53	8	2
RRsLug 0710A0363	M	68	2a	0	0	2	IB	1	48	4	4

RRsLug 0705A0322	M	67	2a	1	0	3	IIB	1	38	8	3
RRsLug 0809A0565	M	55	2b	2	0	3	IIIA	1	14	4	6
RRsLug 0808A0557	M	63	1	0	0	2	IA	0	25	8	3
RRsLug 0711A0405	M	65	2a	0	0	2	IB	0	59	4	2
RRsLug 0706A0333	M	65	2a	1	0	3	IIB	1	25	4	6
RRsLug 0712A0423	M	67	2a	2	0	2	IIIA	0	57	6	4
RRsLug 0905A0707	F	67	1	0	0	2	IA	0	40	8	2
RRsLug 0706A0347	M	74	2a	0	0	3	IB	1	56	4	3
RRsLug 0712A0426	M	71	3	1	0	2	IIIA	1	33	4	0
RRsLug 0807A0550	M	52	2a	2	0	2	IIIA	1	14	4	6
RRsLug 0905A0699	M	65	3	2	0	3	IIIA	1	8	4	4
CRsLug 0609A0172	M	57	2a	0	0	2	IB	0	80	4	6
CRsLug 0412A0059	M	60	2b	0	0	3	IIA	0	92	4	2
RRsLug 0803A0481	M	58	2a	1	0	2	IIB	0	53	4	0
RRsLug 0612A0209	M	65	3	1	0	3	IIIA	1	14	4	0
RRsLug 0901A0617	M	48	2a	2	0	2	IIIA	0	44	4	6
RRsLug 0609A0199	F	53	2a	1	0	2	IIB	0	72	4	2
RRsLug 0709A0353	F	51	2a	2	0	2	IIIA	1	29	4	2
RRsLug 0612A0211	F	67	2a	1	0	2	IIB	1	13	4	3
RRsLug 0709A0355	M	53	2a	2	0	3	IIIA	1	16	4	6

Supplemental Table 3. Limiting dilution in vivo tumor growth of SIRPγ^{high} and SIRPγ$^{low/-}$ A549 cells.

Injected cell number	Tumor incidence (tumors/injected mice)	
	SIRPG^{high} cells	SIRPG$^{low/-}$ cells
5.0×10^{5}	6/6	2/6
1 .0×10^{5}	4/6	1/6
5.0×10^{4}	2/6	0/6
1.0×10^{4}	0/6	0/6
5.0×10^{3}	0/6	0/6
1.0×10^{3}	0/6	0/6

(*n*=6 mice per group).

Supplemental Table 4. Gene profiles of YAP target genes in control and SIRPG knockdown cells.

Hippo-Yap signaling associated genes	
Gene symbol	shSIRPG vs shControl<br>Fold change
BIRC5	-2.47
LAMC2	-2.32
MMP10	-2.23
SLUG	-1.91
SOX2	-1.78
GLI2	-1.73
CYR61	-1.94

Supplemental Table 5. Correlation of SIRPG-YAP expression in LUAD samples.

IHC staining	SIRPG positive	SIRPG negative	Total
YAP positive	89(48.9%)	17(9.3%)	106
YAP negative	37(20.3%)	39(21.4%)	76
Total	126	56	182

Associate significant, $P<0.001$, two tailed Fisher's exact test.

Supplemental Table 6. Sources of cell lines.

Cell Name	*Tissue Type*	*Tissue*	*Phenotype*	*Primary*
U937	Human	Macrophage	Suspension	ATCC
JURKAT	Human	Lymphocyte	Suspension	ATCC
THP-1	Human	Blood	Suspension	ATCC
HBE	Human	Bronchus	Adherent	ATCC
H1299	Human	Lung	Adherent	ATCC
H460	Human	Lung	Adherent	ATCC
HEB	Human	Brain	Adherent	ATCC
T98G	Human	Brain	Adherent	ATCC
A172	Human	Brain	Adherent	ATCC

L02	Human	Liver	Adherent	ATCC
PLC	Human	Liver	Adherent	ATCC
DLD1	Human	Colorectum	Adherent	ATCC
SW620	Human	Colorectum	Adherent	ATCC
Hela	Human	Cervix	Adherent	ATCC
LN18	Human	Brian	Adherent	ATCC
LN229	Human	Brian	Adherent	ATCC
A549	Human	Lung	Adherent	ATCC
H1975	Human	Lung	Adherent	ATCC
L-929	Mouse	Fibroblast	Adherent	ATCC
PANC	Human	Pancreas	Adherent	ATCC
T47D	Human	Breast/Mammary	Adherent	ATCC
LM3	Human	Liver	Adherent	ATCC
HUH7	Human	Liver	Adherent	ATCC
HEK293T	Human	Kidney	Adherent	ATCC
LLC	Mice	lung	Adherent	ATCC
1214	Human	Brain	Adherent	(Southwest Hospital, TMMU)*

*Optimized dissociation protocol for isolating human glioma stem cells from tumor spheres via fluorescence-activated cell sorting. Cancer Lett. 2016, 10;377(1).

Supplemental Table 7. Reagents and antibodies.

No	Catalog	Antibody	Sources	Dilution	Corp.
1	sc-53604	SIRPγ (LSB2.2)	Mouse	1:500	Santa cruz
2	sc-53112	SIRPγ (OX117)	Mouse	1:500	Santa cruz
3	Sc-69786	IgG	Mouse	100μg/ml	Santa cruz
4	Ab96064	Anti-CD172g	Rabbit	1:500	Abcam
5	Ab32618	Anti-GRP78 Bip	Rabbit	1:10000	Abcam
6	Ab3283	Anti-CD47	Mouse	1:1000	Abcam
7	Ab52771	Anti-YAP1	Rabbit	1:1000	Abcam
8	Ab76252	Anti-YAP1(phosphor S127)	Rabbit	1:1000	Abcam
9	#3579	SOX2	Rabbit	1:1000	Cell Signaling
10	#3682	MST1	Rabbit	1:1000	Cell Signaling
11	49332	Phospho-MST1(Thr183)/MST2(Thr180)	Rabbit	1:1000	Cell Signaling
12	#3477	LATS1	Rabbit	1:1000	Cell Signaling
13	#9157	Phospho-LATS1(Ser909)	Rabbit	1:1000	Cell Signaling
14	#4903	NANOG	Rabbit	1:1000	Cell Signaling
15	#2750	FOU5F1	Rabbit	1:1000	Cell Signaling
16	#14074	YAP	Rabbit	1:400	Cell Signaling
17	#4970	β-actin	Rabbit	1:1000	Cell Signaling
18	#5174	GAPDH	Rabbit	1:1000	Cell Signaling
19	#2038	PP2Ac	Rabbit	1:1000	Cell Signaling
20	556046	PE Mouse Anti-Human CD47	Mouse	1×10^6/20μl	BD Biosciences

21	05-1561-11	Rat Anti-Mouse CD11b-APC	Rat	1:50	SouthernBiotech
22	565410	PE Rat Anti-Mouse F4/80	Rat	1:50	BD Bioscience
23	SAB4500497	Anti-M-CSF	Mouse	50 ng/ml	Sigma Aldrich
24	Ab226918	Anti-IL-1β	Rat	1:1000(IB)	Abcam
25	#3578	CD44	Rat	1:1000(IB)	Cell Signaling
26	#64326	CD133	Rat	1:1000(IB)	Cell Signaling
27	Sc-136067	SIRPα	Mouse	1:600(IB)	Santa cruz
28	#9750	IGF-I Receptor β	Rat	4μg/200μl	Cell Signaling
29	595337	SIRPγ	Mouse	1:1000(IB)	R&D
30	MAB4486	SIRPγ	Mouse	1:1000(IB)	R&D
31	Ab108415	Anti-CD47	Mouse	1:1000(IB)	Abcam
32	Ab54429	Anti-GM-CSF	Mouse	1:1000(IB)	Abcam
33	SC-52003	Antibody IgG	Mouse	4μg/200μl	Santa cruz
34	11857-1-AP	SIRPγ	Rabbit	1×10^{6}/20μl	Proteintech
36	13584-1-AP	YAP	Rabbit	1:1000	Proteintech
37	ab79351	Anti-SOX2	mouse	1:200	Abcam
38	AF-315-05	IFN-γ		40ng/ml	Peprotech
39	300-03	GM-CSF		30ng/ml	Peprotech
40	200-01B	IL-1β		100ng/ml	Peprotech
41	297-473-0	LPS		200ng/ml	Sigma Aldrich
42	A21422	Goat anti-Mouse IgG (H+L) Cross-Adsorbed Secondary Antibody, Alexa Fluor 555		1:1000	Invitrogen
43	A32733	Goat anti-Rabbit IgG (H+L) Highly Cross-Adsorbed Secondary Antibody, Alexa Fluor Plus 647		1:1000	Invitrogen
44	TR-1003	Polybrene Infection		4 μg/ml	Sigma Aldrich
45	A1113802	Puromycin Dihydrochloride		4 μg/ml	Thermo Fisher
46	10687010	Hygromycin B		800 μg/ml	Thermo Fisher
47	PKH26PCL	PKH26 Red Fluorescent Cell Linker Kit		4μl/10^{7}cell	Sigma Aldrich
48	21888	CFSE		1:5000	Sigma Aldrich
49	AF-100-15	EGF			Peprotech
50	AF-100-18B	FGFb			Peprotech
51	#72302	RHO\ROCK pathway inhibitor Y-27632			STEMCELL Tech
52	#07913	Dispase			STEMCELL Tech
53	#07426	Collagenase Type IV			STEMCELL Tech
54	# 352350	Cell strainer 70um Nylon			BD Falcon
55	#555899	lysing Buffer			BD Biosciences
56	#354234	Matrigel			BD BioCoat
57	G9681	CellTiter-Glo® 3D Cell Viability Assay			CellTiter-Glo
58	354249	Coning®Collagen I，High Concentration，Rat Tail			BD BioCoat
59	L3000015	Lipofectmine™ 3000 Transfection Reagent			Invitrogen

60	LT007	Lenti-Pac™ Lentivirus Concentration Solution			Genecopoeia
61	Ab174448	Human GM-CSF ELISA Kit（CSF2）			Abcam
62	Ab46052	Human IL-1 beta ELISA Kit			Abcam
63	H3569	Hoechst 33258			Thermo Fisher
64	01700	ALDEFLUOR stem identification kit			Stem cell

Supplemental Table 8. Target sequences and ORF expression clone for *SIRPG*, *YAP*, *IL1B*, *GM-CSF*, *CD47* and *PP2Ac*.

Gene (Accession no.)		Primer sequence (5’→3’)
SIRPG (NM_080816)	Target sequence	1-CCGGGCTCCTGTTGGTCACAGTTCTCAAGAGAAACTGTGACCAACAGGAGCTTTTTG<br>2-CCGGCCTGTGGTTCAGAGGAGTTCTCAAGAGAAACTCCTCTGAACCACAGGTTTTTG<br>3-CCGGGCCGGGAATTAATCTACAATTCAAGAGATTGTAGATTAATTCCCGGCTTTTTG
	Delete C terminal	F:5‘ CCCATCTACGTCCCCTGGTGACCAACTTTCTTGTAC 3’<br>R:5‘GGGTAGATGCAGGGGACCACTGGTTGAAAGAACATG 3’
	DeleteC&M terminal	F:5‘ GAGCTCAGATGCTACCCCTTGACCAACTTTCTTGTAC 3’<br>R:5‘CTCGAGTCTACGATGGGGAACTGGTTGAAAGAACATG 3’
MST1	Delete C terminal	F:5‘ATGGAGACGGTACAGCTGAG 3’
		R:5‘ GTAGTCTCCATCCTGTGGTA 3’
YAP (NM_006106)	Target sequence	1-CCGGCCCAGTTAAATGTTCACCAATCTCGAGATTGGTGAACATTTAACTGGGTTTTTG<br>2-CCGGCAGGTGATACTATCAACCAAACTCGAGTTTGGTTGATAGTATCACCTGTTTTTG<br>3-CCGGGCCACCAAGCTAGATAAAGAACTCGAGTTCTTTATCTAGCTTGGTGGCTTTTTG
PP2Ac (NM_002715)	Target sequence	1-CCGGCACACAAGTTTATGGTTTCTACTCGAGTAGAAACCATAAACTTGTGTGTTTTT<br>2-CCGGTGGAACTTGACGATACTCTAACTCGAGTTAGAGTATCGTCAAGTTCCATTTTT<br>3-CCGGCCCATGTTGTTCTTTGTTATTCTCGAGAATAACAAAGAACAACATGGGTTTTT
IL1B (NM_000576)	Target sequence	1-CCGGCTGACTTCACCATGCAATTTGCTCGAGCAAATTGCATGGTGAAGTCAGTTTTTG<br>2-CCGGTCACCTCTCCTACTCACTTAACTCGAGTTAAGTGAGTAGGAGAGGTGATTTTTG<br>3-CCGGATCAATAACAAGCTGGAATTTCTCGAGAAATTCCAGCTTGTTATTGATTTTTTG
GM-CSF (NM_000758)	Target sequence	1-CCGGATGATGGCCAGCCACTACAAGCTCGAGCTTGTAGTGGCTGGCCATCATTTTTTG<br>2-CCGGCCCAGATTATCACCTTTGAAACTCGAGTTTCAAAGGTGATAATCTGGGTTTTTG<br>3-CCGGGCAACCCAGATTATCACCTTTCTCGAGAAAGGTGATAATCTGGGTTGCTTTTTG
CD47 (NM_001777)	Target sequence	1-CCGGGCCTTGGTTTAATTGTGACTTCCACACCAAGTACAATTAAGCCAAGGCTTTTTTA<br>2-CCGGGCACAATTACTTGGACTAGTTGGTGTGGAACTAGTCCATAATGTGTTTTTTA<br>3-CCGGGCATCTCTGTTAGTTCTATTGGTGTGGAATAGAACTAAGCAGAGATGCTTTTTTA<br>4-CCGGATATCTCTGGGTAATCACCAGCTCGAGCTGGTGATTACCCAGAGATATTTTTTTA
SIRPG (NM_080816)	ORF clone	Forward: 5' CTCCATAGAAGACACCGAC 3'<br>Reverse: 5' CATATAGACAAACGCACAC3'
YAP (NM_001130145)<br>*CD47* (NM_001777.3)	ORF clone<br>ORF clone	Forward:5' CCCAGTCACGACGTTGTAAAACG-3'<br>Reverse:5' TAACATCAGAGATTTTGAGACAC 3'<br>Forward:5' GGACTTTCCAAAATGTCG 3'<br>Reverse:5' ATTAGGACAAGGCTGGTGGG 3'

Supplemental Table 9. Sequences of the primers used for qRT-PCR.

Gene (Accession no.)	Primer sequence (5'→3')	Amplification size (bp)
ARG1	F: CCACAGTCTGGCAGTTGGAAG<br>R: GGTTGTCAGGGGAGTGTTGATG	106
CCL20	F: TCCTGGCTGCTTTGATGTCA<br>R: CAAAGTTGCTTGCTGCTTCTGA	69
CD47	F: GCGATTGGATTAACCTCCTTCGTCA<br>R: CCATGCATTGGTATACACGCCGC	113
CXCL2	F: CTGCTGCTCCTGCTCCTG<br>R: TCCTTCTGGTCAGTTGGATTTG	276
CYR61	F: ACTTCATGGTCCCAGTGCTC<br>R: AAATCCGGGTTTCTTTCACA	100
GM-CSF	F: AAATGTTTGACCTCCAGGAGCC<br>R: GAGGGCAGTGCTGCTTGTAG	135
IL1B	F: GAGCAACAAGTGGTGTTCTCC<br>R: AACACGCAGGACAGGTACAG	110
LAMC2	F: ACTGGAGCAGAAGCTTTCCC<br>R: GTATTGTAGCAGCCTGGGGG	195
MMP10	F: CACAGTTTGGCTCATGCCTA<br>R: TGCCTGATGCATCTTCTGTC	94
NANOG	F: CAATGGTGTGACGCAGAAGG<br>R: TGCACCAGGTCTGAGTGTTC	197
POU5F1	F: GCAGCGACTATGCACAACGA<br>R: CCAGAGTGGTGACGGAGACA	195
SIRPG	F: ACTGCGCTGCTCCTCATA<br>R: GCTTCTCACTGGCTCATTCT	164
SOX2	F: CAGCCCATGCACCGCTACGACG<br>R: CACCGAACCCATGGAGCCAAGAGC	147
STAT3	F: TGCCGGAGAAACAGGATGGC<br>R: CTCCATTGGGAAGCTGTCACT	102
CD133	F: CACTACCAAGGACAAGGCGTTC<br>R: CAACGCCTCTTTGGTCTCCTTG	151
CD44	F: CCAGAAGGAACAGTGGTTTGGC<br>R: ACTGTCCTCTGGGCTTGGTGTT	130
GAPDH	F: CAAGCTCATTTCCTGGTATGAC<br>R: CAGTGAGGGTCTCTCTCTTCCT	142

Supplemental Table 10. Relevant species alignment using the *PTGER2* DNA and the PCR primers.

Gene (Accession no.)	Primer sequence (5'→3')	Amplification size (bp)
HUMAN *PTGER2*	F: GCTGCTTCTCATTGTCTCGG<br>R: GCCAGGAGAATGAGGTGGTC	189
MOUSE *PTGER2*	F: CCTGCTGCTTATCGTGGCTG<br>R: GCCAGGAGAATGAGGTGGTC	186

The primers were used to test the ratio of human to mouse DNA in sorted mouse xenograft macrophages.

Supplemental Figure Legends

Figure S1. SIRPγ serves as a CSLCs marker and promotes tumor growth.

(A) Gene expression profiles from microarray data of A549 monolayer cells and sphere cells. Genes with fold changes in mRNA expression were used to generate the heatmap. **(B** and **C)** qRT-PCR analysis of indicated genes in monolayer and sphere (B) or ALDH$^+$ and ALDH$^-$ H1975 cells (C). (**D** and **E**) Immunoblotting analysis of indicated proteins in monolayer and sphere (D) or ALDH$^+$ and ALDH$^-$ H1975 cells (E). **(F)** The differential plot of SIRPγ across 36 cancer types. Red: tumor samples; Blue: normal samples; Gray: missing data. **(G)** Flow cytometry analysis of the number of SIRPγ^{high} or CD47^{high} A549 and H1975 cells. **(H)** Stem cell sphere assay of SIRPγ$^{low/-}$ and SIRPγ^{high} H1975 cells. 1×10^3 cells/well. Scale bar, 50 μm. **(I)** Limiting dilution in vivo tumor growth of SIRPγ^{high} and SIRPγ$^{low/-}$ A549 cells (1×10^5 or 5×10^5 inoculated cells/mice mean ± s.d., *n*=6 mice per group). **(J)** Tumor xenograft growth of control and SIRPγ knockdown A549 cells (1×10^6 inoculated cells/mice, mean ± s.d., *n* =5 mice). **(K)** Immunoblotting analysis of indicated proteins in SIRPγ$^{low/-}$ and SIRPγ^{high} or monolayer and sphere H1975 cells. All experiments were carried out at least in triplicate and the data are presented as the mean ± s.d. * $P < 0.05$, ** $P < 0.01$, paired (B, C, J) or unpaired (H), 2-tailed Student's *t* test.

Figure S2. SIRPγ is highly expressed in various types of stem cell spheres.

(A) qRT-PCR analysis of *SIRPG* expression in control and SIRPγ knockdown A549 cells. **(B** and **C)** Immunoblotting analysis of indicated proteins in control and SIRPγ knockdown A549 (B) and H1975 (C) cells by using different well-characterized SIRPγ-specific monoclonal antibodies. **(D)** Immunoblotting analysis of indicated proteins in vector and SIRPγ overexpression A549 cells. (**E** and **F)** qRT-PCR analysis of *SIRPG* expression in JURKAT cells, normal cells, cancer cells and cancer sphere cells. **(G)** Stem cell sphere assay of control and SIRPγ knockdown LM3 cells (1×10^3cells/well). **(H)** Stem cell sphere assay of vector and SIRPγ overexpression LM3 cells (1×10^3 cells/well). **(I)** Dot blot analysis of SIRPα and SIRPγ expression in A549 cells. **(J)** Immunoblotting analysis of indicated proteins in monolayer and

sphere cells of different cancer types. All experiments were carried out at least in triplicate and the data are presented as the mean ± s.d. * $P < 0.05$, ** $P < 0.01$, paired, 2-tailed Student's *t* test (H) or 1-way ANOVA (A, F, G).

Figure S3. SIRPγ serves as a negative upstream regulator of the MST1/LATS1 axis to promote YAP activation.

(A) Gene-gene interaction network. **(B** and **C)** Immunoprecipitation analysis of the interaction between SIRPγ, PP2A and MST1 in H1975 cells. **(D** and **E)** Immunoprecipitation analysis of the interaction between SIRPγ, PP2A and MST1 in control and SIRPγ knockdown H1975 cells. Data are from one experiment representative of three independent experiments. **(F** and **G)** Immunoblotting analysis of SIRPγ and YAP expression of lung adenocarcinoma-derived organoids in indicated groups. **(H)** SIRPγ regulates YAP nuclear translocation and sustains SOX2 expression. Immunofluorescence staining of YAP (red) and SOX2 (green) in control and SIRPγ knockdown A549 cells (treated without MG132). Nuclei stained with DAPI (blue) are shown. Scale bars, 100μm. **(I-K)** Quantitative analysis of YAP and SOX2 fluorescence intensity in control and SIRPγ knockdown A549 cells. Quantitative analysis of YAP fluorescence intensity location in cytoplasm (cyto) and nucleus (neu) of A549 cells. **(L)** Immunofluorescence staining of indicated proteins in control and SIRPγ knockdown A549 cells (treated with MG132 10μM for 6 hours). Nuclei stained with DAPI (blue) are shown. Scale bars, 100μm. **(M-O)** Quantitative analysis of YAP fluorescence intensity location in cytoplasm and nucleus between control and SIRPγ knockdown A549 cells. Quantitative analysis of SOX2 fluorescence intensity location in nucleus between control and SIRPγ knockdown A549 cells. All experiments were carried out at least in triplicate and the data are presented as the mean ± s.d. * $P < 0.05$, ** $P < 0.01$, paired (I-K, M, N) or unpaired (O), 2-tailed Student's *t* test.

Figure S4. SIRPγ interacts with C-terminal region of MST1.

(A and **B)** Immunoprecipitation analysis of the interaction between SIRPγ, PP2A and MST1 in vector, MST1, MST1-C terminal deletion mutant overexpressing 293T and A549 cells.

Figure S5. SIRPγ positively regulates CD47, IL-1β and GM-CSF expression.

(A) qRT-PCR analysis of indicated genes in control and SIRPγ knockdown H1975 cells. **(B)** qRT-PCR analysis of indicated genes in SIRPγ$^{low/-}$ and SIRPγ^{high} H1975 cells. **(C)** qRT-PCR analysis of indicated genes in vector and SIRPγ overexpression H1975 cells. **(D)** Immunoblotting analysis of indicated proteins in control and SIRPγ knockdown H1975 cells. **(E)** Immunoblotting analysis of indicated proteins in vector and SIRPγ overexpression. **(F)** Co-culture schematic diagram of vector and SIRPγ overexpression H1975 cells and immunoblotting analysis of indicated proteins in vector co-cultured with vector and vector co-cultured with SIRPγ overexpression H1975 cells. All experiments were carried out at least in triplicate and the data are presented as the mean ± s.d. * $P < 0.05$, ** $P < 0.01$, paired, 2-tailed Student's *t* test.

Figure S6. SIRPγ inhibits phagocytosis through YAP signaling.

(A) Phagocytosis of SIRPγ$^{low/-}$ and SIRPγ^{high} FITC-positive H1975 cells by bone marrow derived macrophages (BMDMs). **(B)** Phagocytosis of SIRPγ$^{low/-}$ and SIRPγ^{high} CFSE-labeled A549 cells by bone marrow derived macrophages (BMDMs). **(C)** Phagocytosis of SIRPγ$^{low/-}$ and SIRPγ^{high} CFSE-labeled H1975 cells by bone marrow derived macrophages (BMDMs). **(D)** Phagocytosis of control and SIRPγ knockdown CFSE-labeled H1975 cells with or without YAP overexpression. **(E)** Phagocytosis of vector and SIRPγ overexpression CFSE-labeled H1975 cells with or without YAP knockdown. **(F)** Phagocytosis of vector and SIRPγ overexpressing CFSE-labeled H1975 cells with or without IL-1β or GM-CSF knockdown. **(G)** Phagocytosis assay of vector and SIRPγ overexpressing H1975 cells with or without YAP knockdown by based on CFSE-labeled H1975 cells and PKH26-labeled BMDM. **(H)** Phagocytosis assay of control and SIRPγ knockdown H1975 cells with or without YAP

overexpression based on CFSE-labeled H1975 cells and PKH26-labeled BMDM. **(I)** Phagocytosis assay of vector and SIRPγ overexpression H1975 cells with or without IL-1β or GM-CSF knockdown by PKH26 staining. All experiments were carried out at least in triplicate and the data are presented as the mean ± s.d. * $P < 0.05$, ** $P < 0.01$, paired, 2-tailed Student's *t* test (A-C) or 1-way ANOVA (D-I).

Figure S7. SIRPγ inhibits phagocytosis through CD47 signaling.

(A) Phagocytosis of vector and SIRPγ overexpression CFSE-labeled A549 cells with or without CD47 knockdown. **(B)** Phagocytosis of control and SIRPγ knockdown CFSE-labeled A549 cells with or without CD47 overexpression. **(C)** Phagocytosis of vector and SIRPγ overexpression CFSE-labeled H1975 cells with or without CD47 knockdown. **(D)** Phagocytosis of control and SIRPγ knockdown CFSE-labeled H1975 cells with or without CD47 overexpression. **(E)** Phagocytosis assay of vector and SIRPγ overexpressing A549 cells with or without CD47 knockdown based on CFSE-labeled A549 and PKH26-labeled BMDM. **(F)** Phagocytosis assay of control and SIRPγ knockdown A549 cells with or without CD47 overexpression based on CFSE-labeled A549 and PKH26-labeled BMDM. **(G)** Phagocytosis assay of vector and SIRPγ overexpression H1975 cells with or without CD47 knockdown based on CFSE-labeled H1975 and PKH26-labeled BMDM. **(H)** Phagocytosis assay of control and SIRPγ knockdown H1975 cells with or without CD47 overexpression based on CFSE-labeled H1975 and PKH26-labeled BMDM. All experiments were carried out at least in triplicate and the data are presented as the mean ± s.d. * $P < 0.05$, ** $P < 0.01$, 1-way ANOVA.

Figure S8. SIRPγ enables cancer cells to escape from phagocytosis by THP-1-derived macrophages through YAP signaling.

(A) Phagocytosis of CFSE-labeled A549 cells by PKH26-labeled THP1-derived M1 macrophage was assessed by confocal microscopy. Macrophages, red; targets, green. Scale bars, 50μm. THP1-derived macrophages by PMA (100ng/ml) treated for 48h and IFN-γ (20ng/ml), and LPS (240ng/ml) for 48h. **(B)** Statistic analysis of

phagocytosis of Vector and SIRPγ overexpression CFSE-labeled A549 cells for 24h by confocal microscopy. **(C)** Phagocytosis of control and SIRPγ knockdown CFSE-labeled A549 cells by confocal microscopy. **(D)** Flow cytometry analysis of phagocytosis of control, SIRPγ knockdown or YAP1 knockdown CFSE-labeled A549 cells by M1 macrophage. **(E)** Flow cytometry analysis of phagocytosis of control, SIRPγ knockdown or YAP1 knockdown CFSE-labeled H1975 cells by M1 macrophage. **(F)** Phagocytosis of control and SIRPγ knockdown CFSE-labeled A549 cells with or without YAP overexpression by confocal microscopy. **(G)** Flow cytometry analysis of phagocytosis of control, SIRPγ knockdown CFSE-labeled A549 cells with or without YAP overexpression by M1 macrophage. All experiments were carried out at least in triplicate and the data are presented as the mean ± s.d. * $P < 0.05$, ** $P < 0.01$, paired, 2-tailed Student's *t* test (B-E) or 1-way ANOVA (F, G).

Figure S9. SIRPγ-YAP axis promotes tumor growth.

(A) H1975 xenograft growth of indicated groups. H1975 cells that had been transfected to stably express firefly luciferase was infected with lentiviruses carrying *SIRPG* overexpression with or without YAP knockdown. Cells were injected subcutaneously to 6-week-old female nude mice (1×10^5 cells per mouse, *n*=4~5 mice per group. **(B** and **C)** Tumor volume (B) and tumor weight (C) of indicated groups. H1975 xenograft growth of indicated groups. **(D)** H1975 cells that had been transfected to stably express firefly luciferase was infected with lentiviruses carrying SIRPγ knockdown with or without YAP overexpression. Cells were injected subcutaneously to 6-week-old female nude mice (1×10^5 cells per mouse, *n*=4~5 mice per group. **(E** and **F)** Tumor volume (E) and tumor weight (F) of indicated groups. All experiments were carried out at least in triplicate and the data are presented as the mean ± s.d. * $P < 0.05$, ** $P < 0.01$, 1-way ANOVA.

Figure S10. Depletion of macrophages regulates SIRPγ-mediated tumorigenesis.

(A-C) Flow cytometry analysis of macrophage percentage in liver, bone marrow or tumor after clodronate-liposome treatment. **(D)** Clodronate-liposome affects the tumor

growth of indicated groups. (**E**) Tumor weight of indicated groups. Mice were injected intravenously with liposomal clodronate or liposomal vehicle (200ul per mice; Yeasen, Shanghai, *n*=6 per group). All experiments were carried out at least in triplicate and the data are presented as the mean ± s.d. * $P < 0.05$, ** $P < 0.01$, unpaired, 2-tailed Student's *t* test (A-C) or 1-way ANOVA (D).

Figure S11. SIRPγ acts through IL-1β and GM-CSF and CD47 axis to regulate phagocytosis and tumorigenesis.

(A and **B)** A549 tumor volume and tumor weight of each of the indicated groups (1×10^6 inoculated cells/mice). **(C** and **D)** A549 xenograft tumor volume and tumor weight of indicated groups (CFSE labelled 1×10^6 inoculated cells/mice, *n*=5 mice per group). **(E)** Phagocytosis in A549 xenografts, represented by the percentage of $CFSE^+F4/80^+$ cells to total $F4/80^+$ cells. All experiments were carried out at least in triplicate and the data are presented as the mean ± s.d. * $P < 0.05$, ** $P < 0.01$, 1-way ANOVA.

Figure S12. SIRPγ-YAP axis promotes lung metastasis.

(A and **B)** Lung metastasis of indicated groups. A549 cells stably expressed with firefly luciferase were infected with lentiviruses carrying control shRNA (shControl), shRNA for *SIRPG* (shSIRPγ), or shRNA for *YAP* (shYAP). Cells (1×10^5 cells per mouse) were injected into the lateral tail vein of 6-week-old female nude mice (*n*=6~7). **(C)** Lung metastasis of indicated groups and the lung weight of shControl, shSIRPγ, and shYAP groups (*n*=6~7). **(D** and **E)** A549 metastasis of indicated groups. Imaging of luciferase in individual mice by IVIS Spectrum and photographs of representative lung, brain and liver from each of the indicated groups (D) and statistical analysis of signal intensity of each of the indicated groups (E). **(F)** The survival of indicated groups (1×10^6 inoculated cells/mice, *n*=5 mice per group). **(G** and **H)** H1975 lung metastasis of indicated groups. Imaging of luciferase in individual mice (G) and statistical analysis of signal intensity of each of the groups (H). H1975 cells that had been transfected to stably express firefly luciferase was infected with lentiviruses carrying SIRPγ overexpression with or without YAP knockdown. Cells

were injected into the lateral tail vein of 6-week-old female nude mice (1×10^5 cells per mouse, *n*=3~5 mice per group. All experiments were carried out at least in triplicate and the data are presented as the mean ± s.d. * $P < 0.05$, ** $P < 0.01$, unpaired, 2-tailed Student's *t* test or log-rank test for survival.

Figure S13. SIRPγ neutralizing antibody targets YAP-SOX2 pathway and CSLCs in vitro.

(A) Immunoblotting analysis of indicated proteins with or without SIRPγ antibody LSB2.20 treatment (4 µg/ml, 2h). **(B)** qRT-PCR analysis of indicated genes with or without LSB2.20 treatment (4 µg/ml, 48h). **(C)** Real-time PCR analysis of *CD47* mRNA in control IgG and LSB2.20 treated cells (4 µg/ml, 48h) with or without IL-1β (100 ng/ml) and GM-CSF (30 ng/ml) treatment for 24h. **(D)** Stem cell sphere assay of A549 cells with or without LSB2.20 treatment. All experiments were carried out at least in triplicate and the data are presented as the mean ± s.d. * $P < 0.05$, ** $P < 0.01$, paired, 2-tailed Student's *t* test (B, D) or 1-way ANOVA (C).

Figure S14. SIRPγ neutralizing antibody promotes phagocytosis but does not regulate the growth and migration ability of T cells.

(A) Phagocytosis of CFSE-labeled A549 cells treated with IgG or SIRPγ antibody (LSB2.20) by PKH26-labeled BMDM was assessed by confocal microscopy. (**B** and **C)** Phagocytosis of IgG Fab, IgG FcR, anti-SIRPγ and anti-IGF1R treated CFSE-labeled A549 cells (B) or H1975 (C) by PKH26-labeled BMDM was assessed by confocal microscopy. (**D** and **E)** A549 xenograft growth of indicated groups (D) and tumor volume of indicated groups (E). CFSE labelled vector and CD47 overexpression A549 cells were inoculated into the abdominal mammary fat pad (1×10^6 inoculated cells/mice, 50µg/mice, *n*=5 mice per group). **(F)** Reduction of the weight of A549 xenografts by LSB2.20 is rescued by CD47 expression. (**G, H)** Promotion of in vivo phagocytosis of A549 xenografts by LSB2.20 is impaired by CD47 overexpression, represented by both enhanced percentage of $CFSE^+F4/80^+$ cells to total $F4/80^+$ cells. (**I)** Trans-endothelial migration ability of human $CD3^+$ T cells by

measuring the number of cells that transmigrating human umbilical vein endothelial cell layer in Boyden chamber after 16 hrs. Cell were treated with control IgG or SIRPγ antibody (4 μg/ml). (**J**) Flow cytometry analysis for cell division times of CFSE-labeled $CD8^+$ T cells from human blood treated with control IgG or SIRPγ antibody (4 μg/ml). (**K**) Proliferation of resting or CD3/CD28 stimulation human PBMCs following treatment with control IgG or SIRPγ antibody (4 μg/ml), cell numbers were counted using a Cell Counting Kit-8 at different time points. All experiments were carried out at least in triplicate and the data are presented as the mean ± s.d. * $P < 0.05$, ** $P < 0.01$, paired, 2-tailed Student's *t* test (I) or 1-way ANOVA (A-C, E, F, H).

Figure S15. SIRPγ promotes tumor growth and SIRPγ blocking antibody inhibits tumor growth in *SIRPG* knock-in mice model.

(**A** and **B**) LSL-human *SIRPG* targeting ES cells are ordered to generate *SIRPG* knock-in mice, which are then bred with $Kras^{LSL\text{-}G12D/+}$ mice. The $Kras^{G12D/+}SIRPG^{KI/+}$ and $Kras^{G12D/+}$ mice were intranasally injected with Ad-Cre for 8 weeks. (**C**) Representative H&E staining of serial sections from lung tumors of $Kras^{G12D/+}SIRPG^{KI/+}$ and $Kras^{G12D/+}$ mice after infection with Ad-Cre.

Figure S16. SIRPγ promotes tumor growth and SIRPγ neutralizing antibody inhibits tumor growth in immunocompetent mice.

(A-D) Flow cytometry analyzed $CD4^+$, $CD8^+$ and $CD45^+$ T cells in the blood before and post human PBMC infusion. **(E)** A549 xenograft growth of indicated groups in the NDG mice infusion with PBMC or not (1×10^6 inoculated cells/mice, *n*=5 mice per group). **(F** and **G)** A549 xenograft weight and volume of indicated groups. **(H)** Immunohistochemical staining of CD4, CD8 and CD68 in the xenograft. **(I-K)** Lung metastasis of indicated groups. SIRPγ overexpression LLC cells were injected into the lateral tail vein of 6-week-C57/BL6 mice and treated with IgG or SIRPG blocking antibody (*n*=5). Imaging of fluorescent in individual mice by IVIS Spectrum and photographs of representative lung from each of the indicated groups (J) and statistical analysis of signal intensity of the indicated groups (K). **(L)** Representative images of SIRPγ overexpression LLC xenograft of indicated groups in C57/BL6 mice

treated with IgG or SIRPG blocking antibody. **(M** and **N)** LLC xenograft tumor weight (M) and tumor volume (N) of indicated groups. **(O)** LSB2.20 promotes phagocytosis in SIRPγ overexpression LLC xenografts, represented by both enhanced percentage of $CFSE^{+}F4/80^{+}$ cells to total $F4/80^{+}$ cells. All experiments were carried out at least in triplicate and the data are presented as the mean ± s.d. * $P < 0.05$, ** $P < 0.01$, unpaired, 2-tailed Student's *t* test (B-D, K, M-O) or 1-way ANOVA (F, G).

Supplemental Figure 1

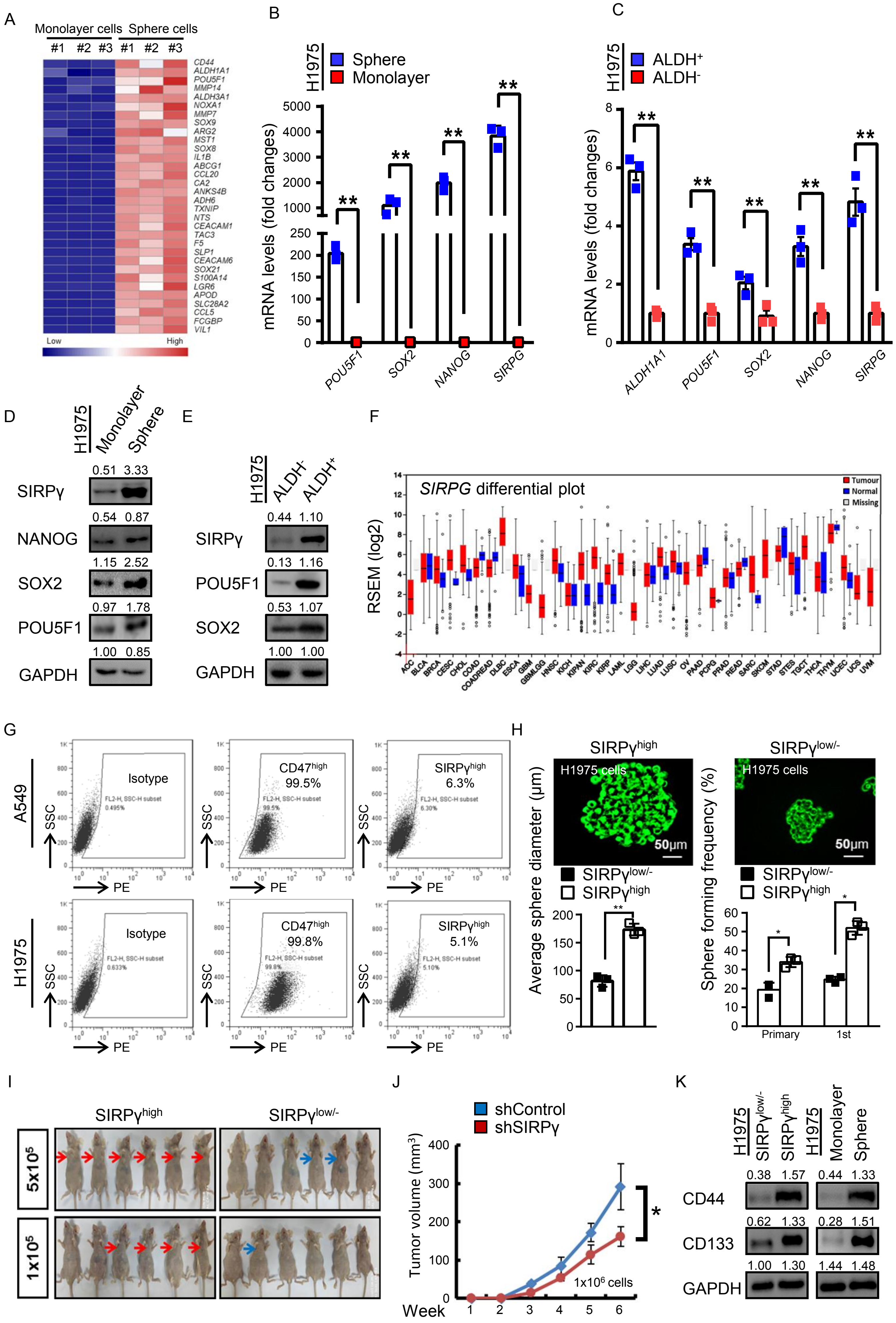

Supplemental Figure 2

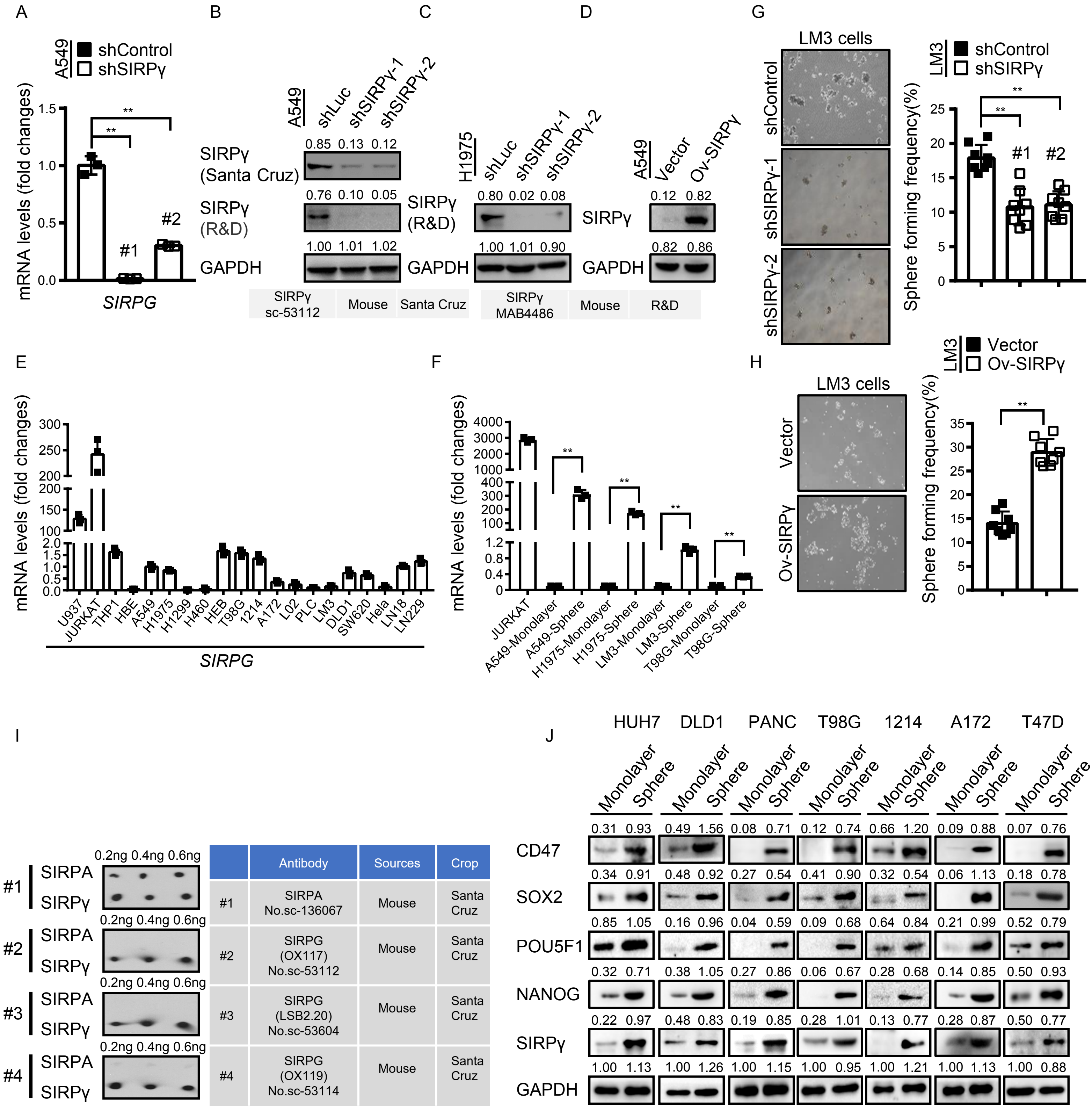

	Antibody	Sources	Crop
#1	SIRPA No.sc-136067	Mouse	Santa Cruz
#2	SIRPG (OX117) No.sc-53112	Mouse	Santa Cruz
#3	SIRPG (LSB2.20) No.sc-53604	Mouse	Santa Cruz
#4	SIRPG (OX119) No.sc-53114	Mouse	Santa Cruz

Supplemental Figure 3

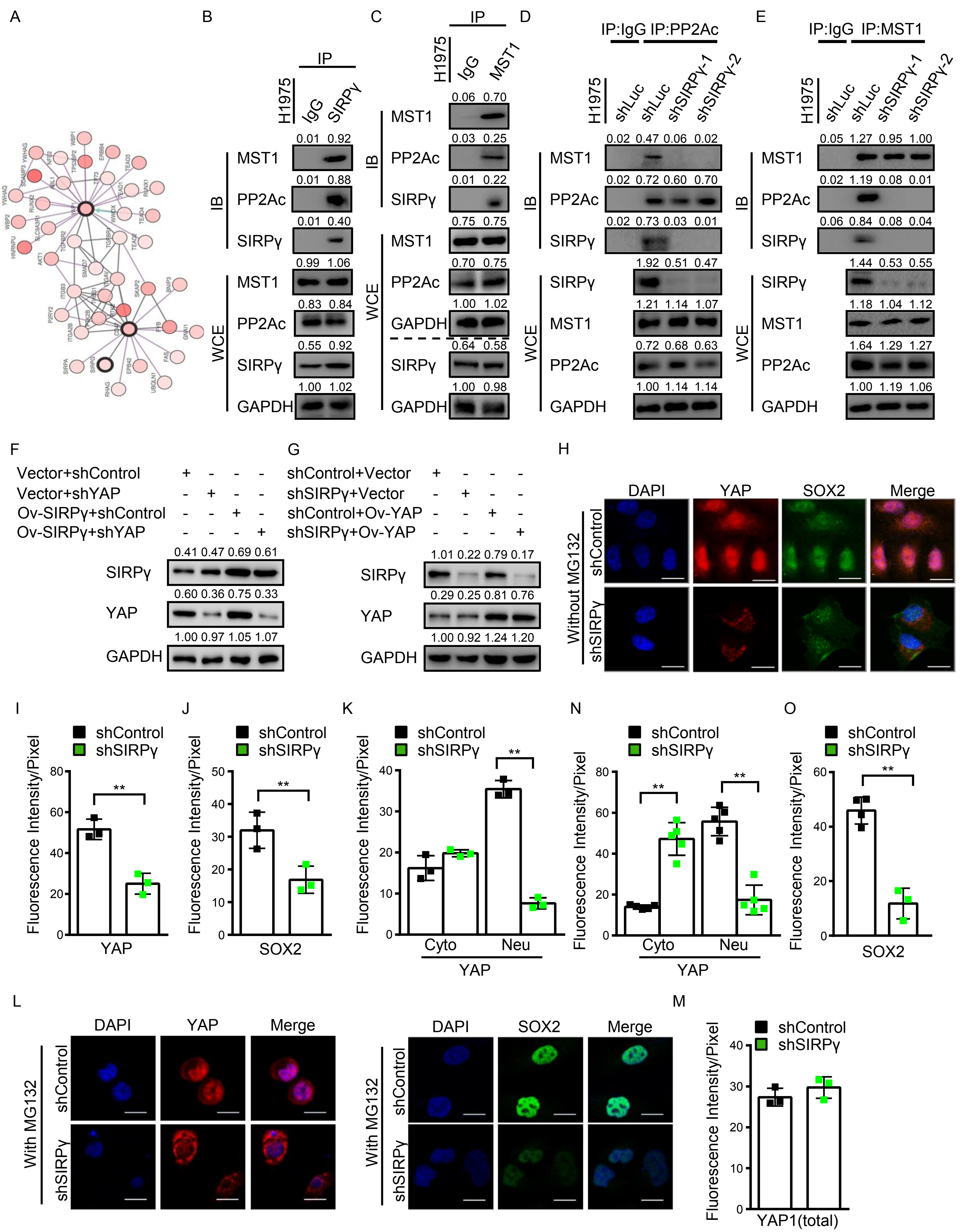

Supplemental Figure 4

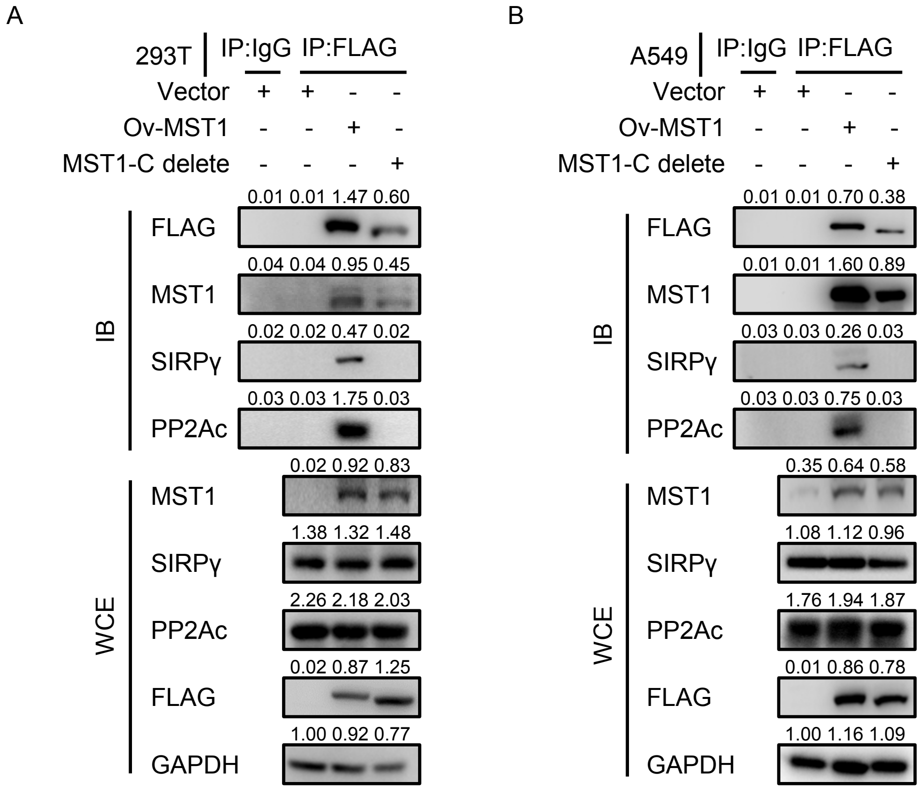

Supplemental Figure 5

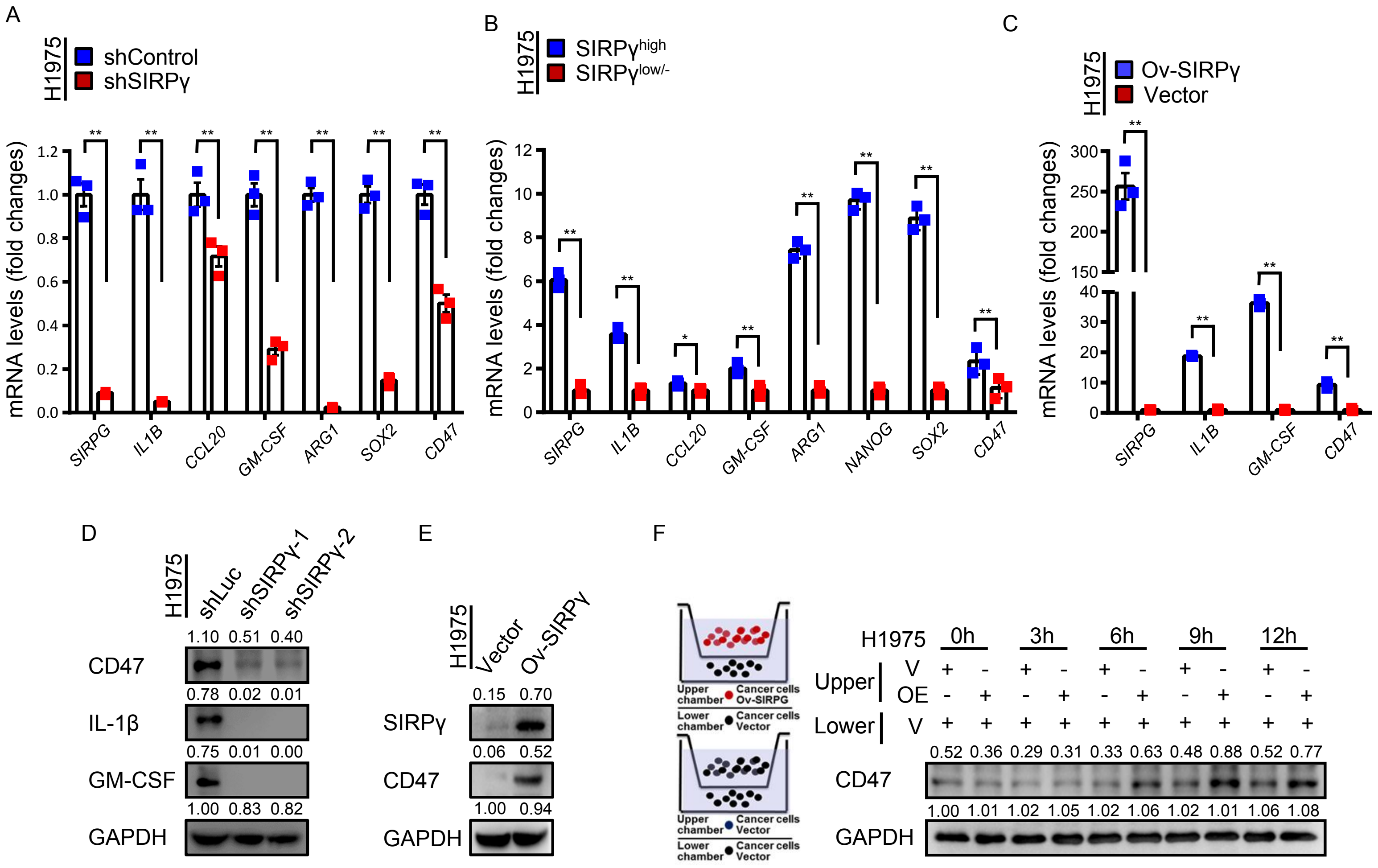

Supplemental Figure 6

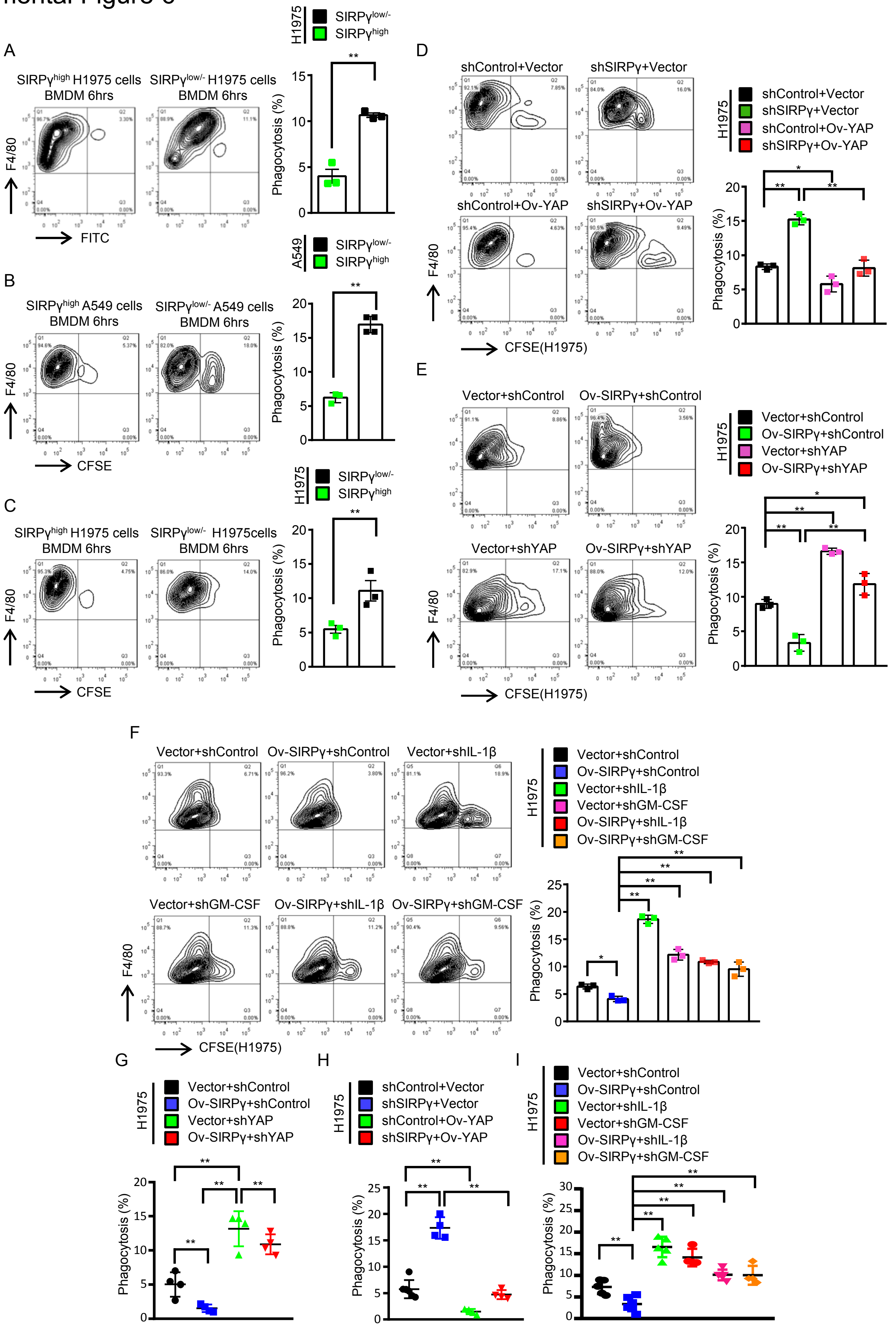

Supplemental Figure 7

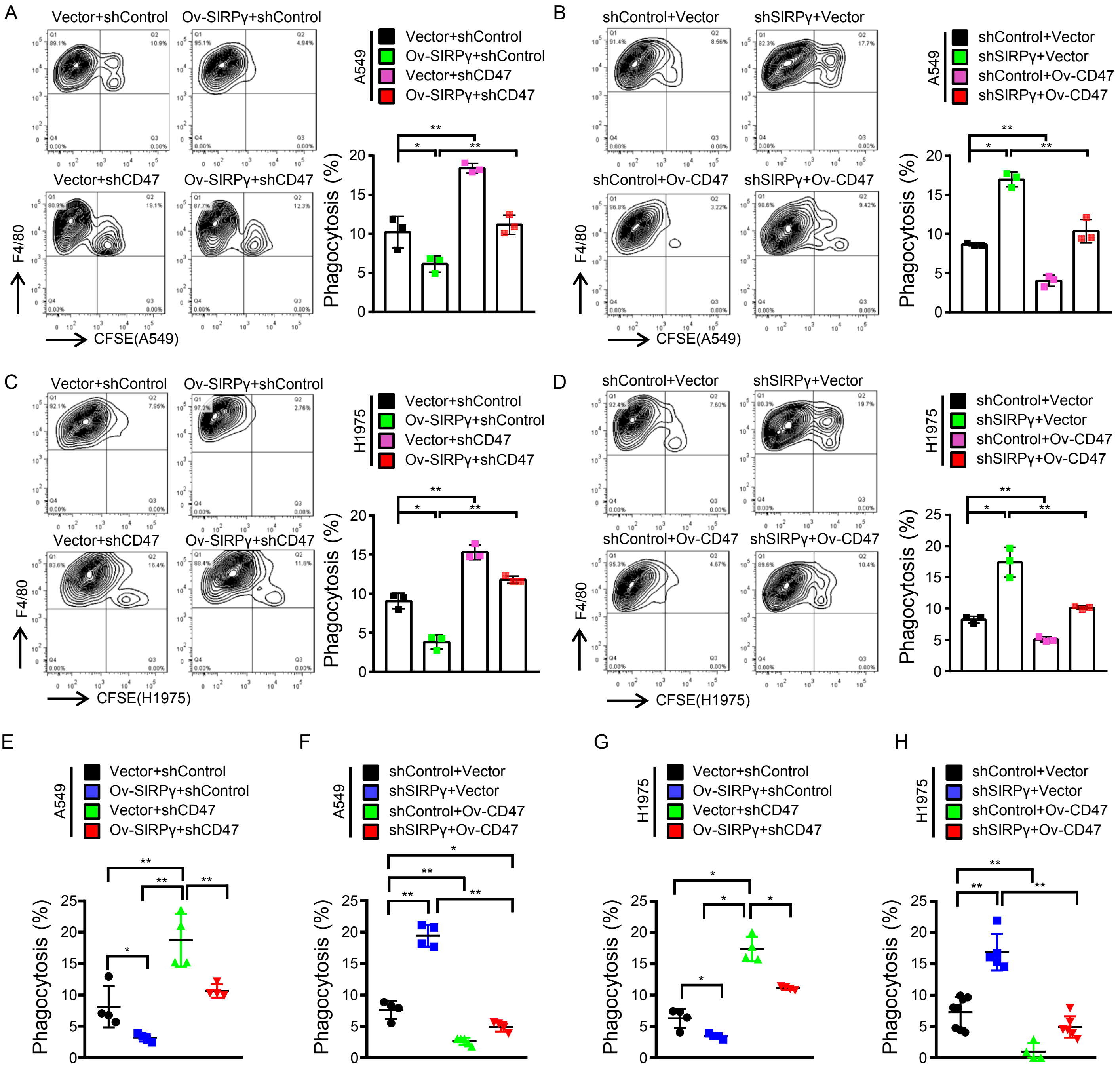

Supplemental Figure 8

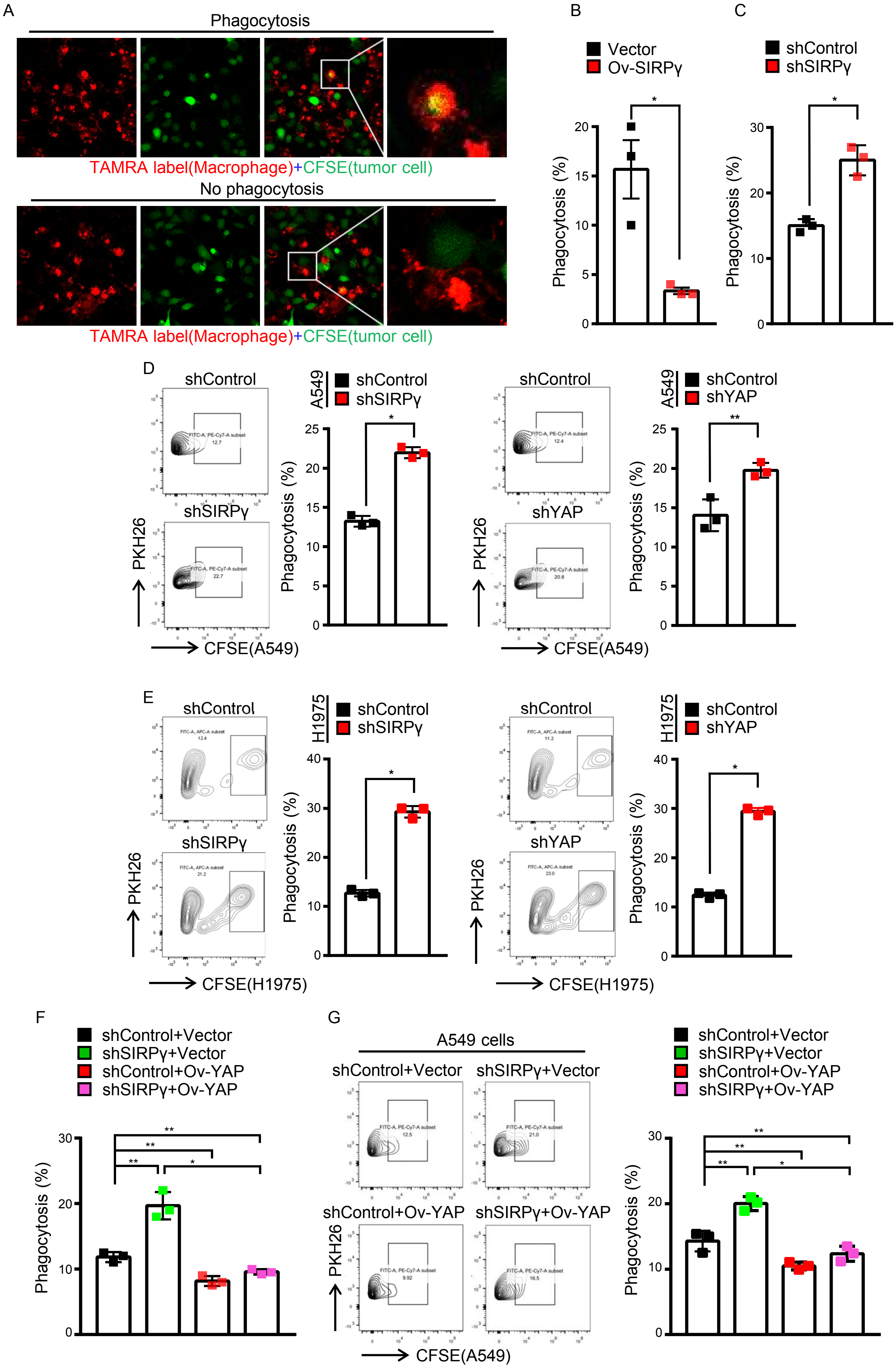

Supplemental Figure 9

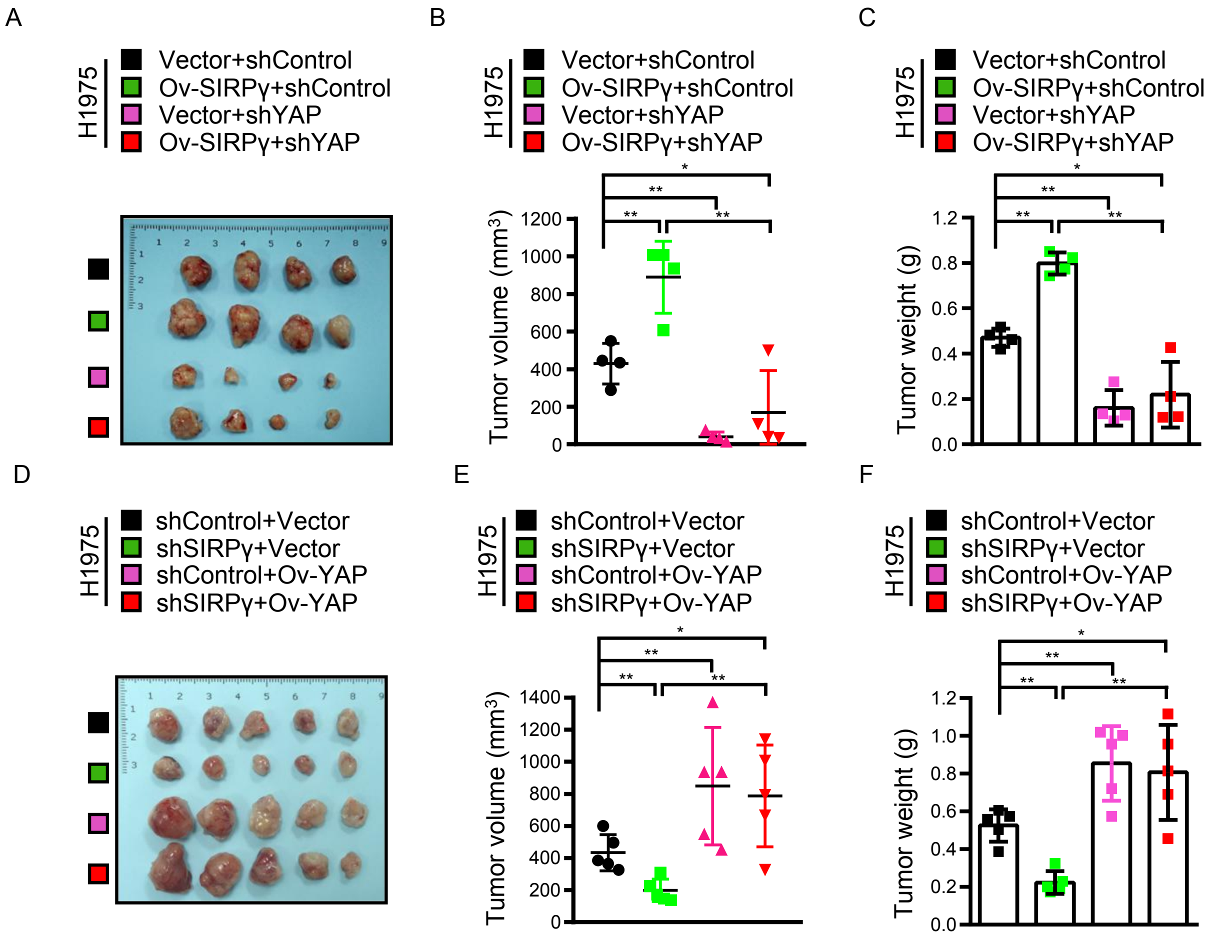

Supplemental Figure 10

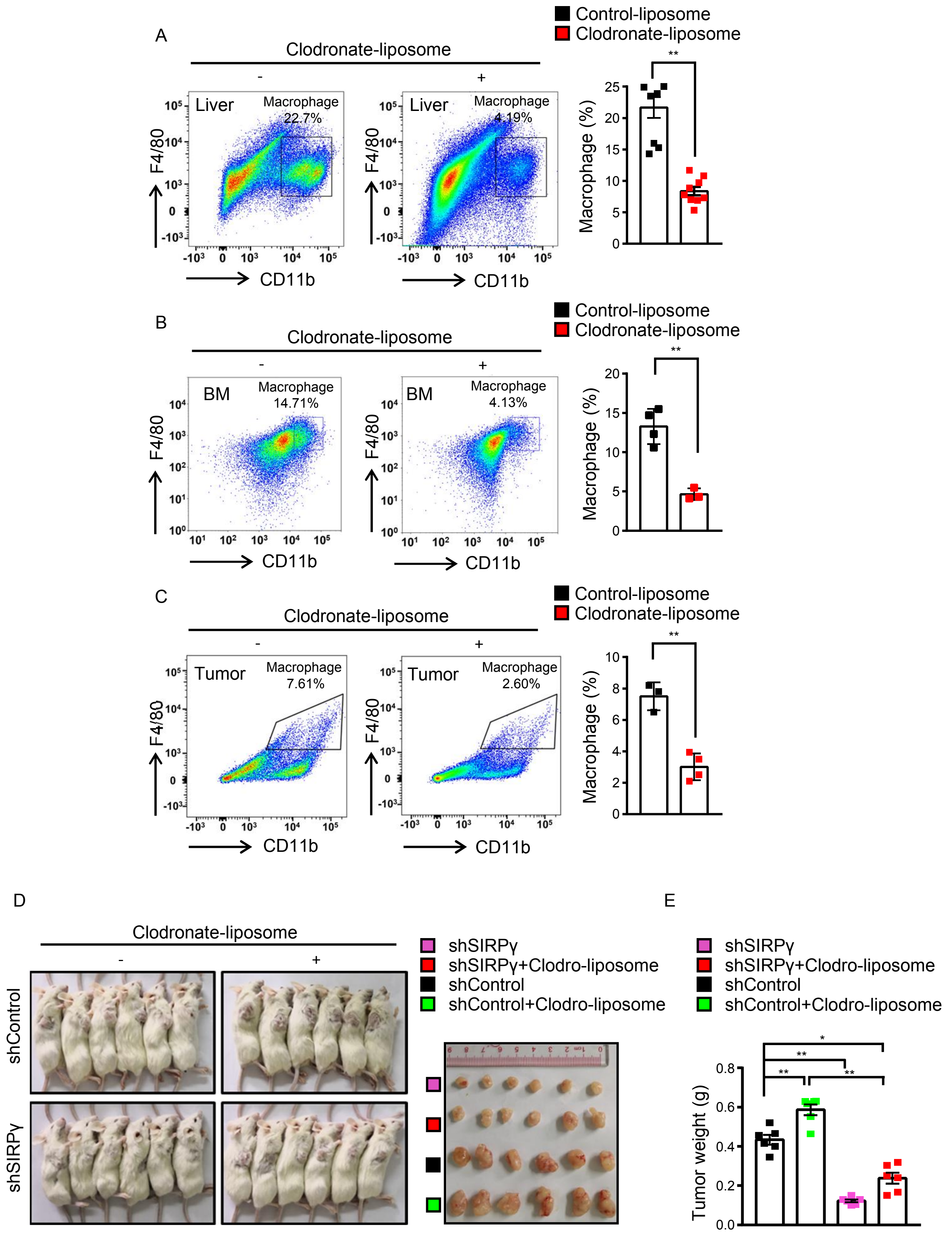

Supplemental Figure 11

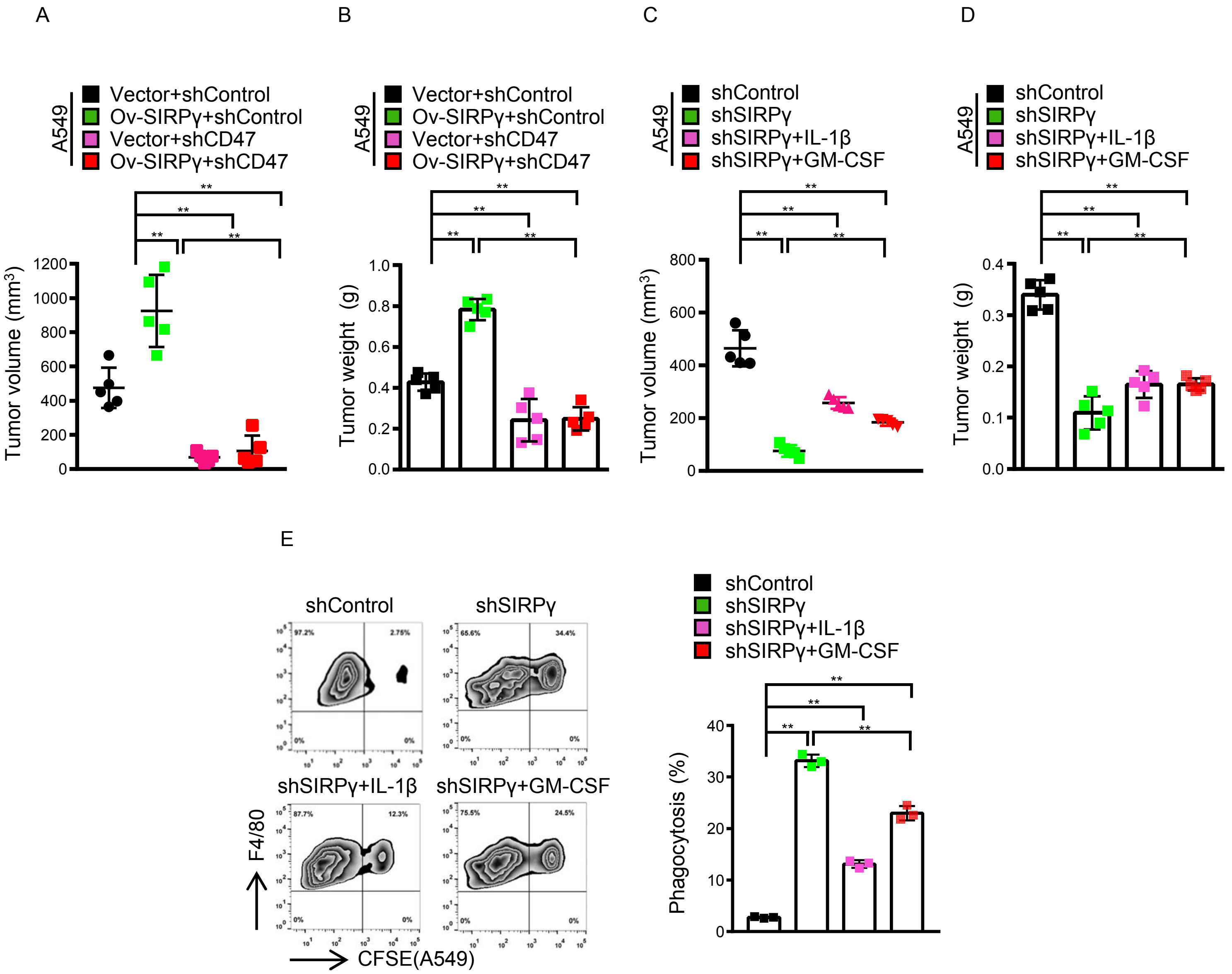

Supplemental Figure12

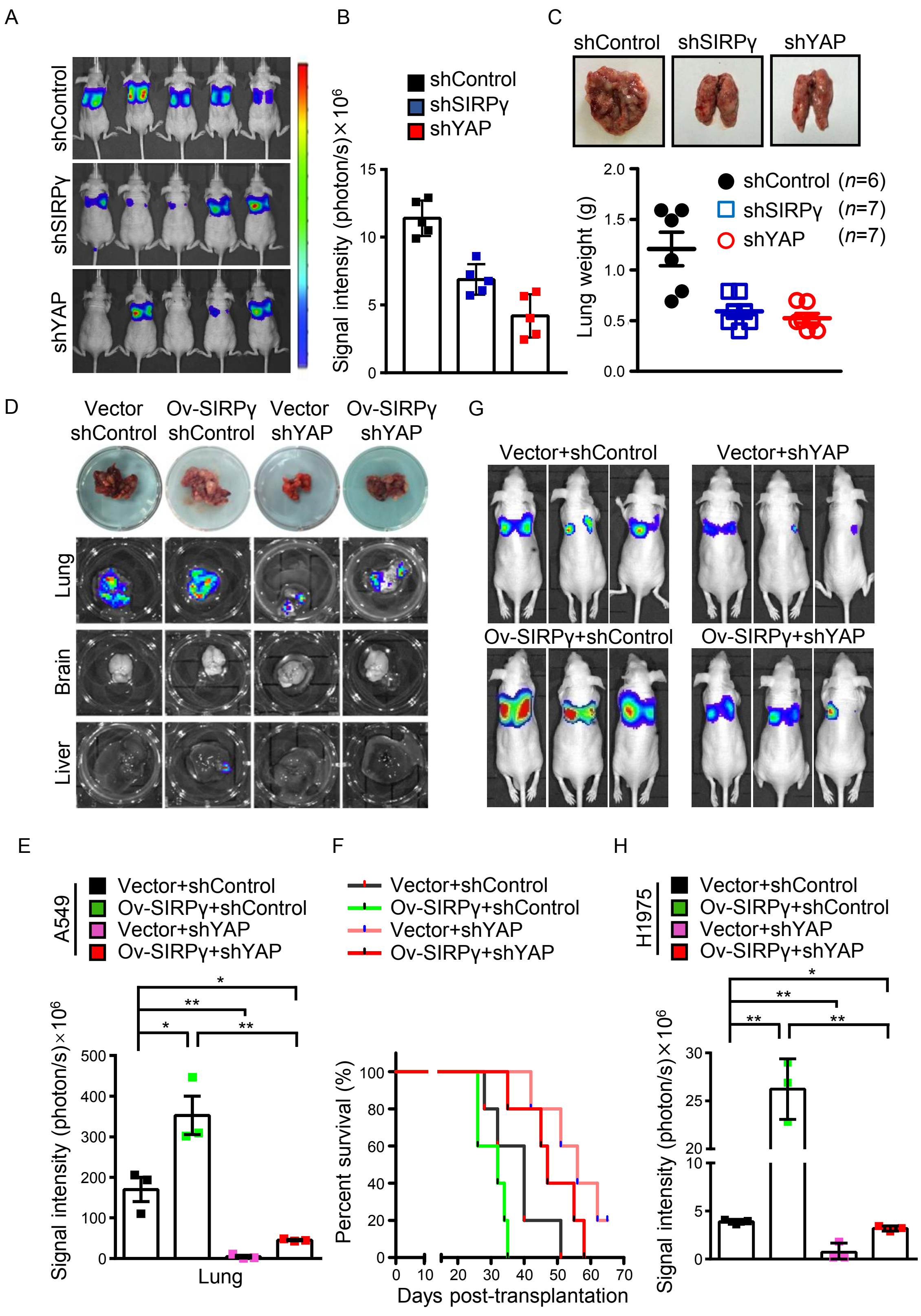

Supplemental Figure13

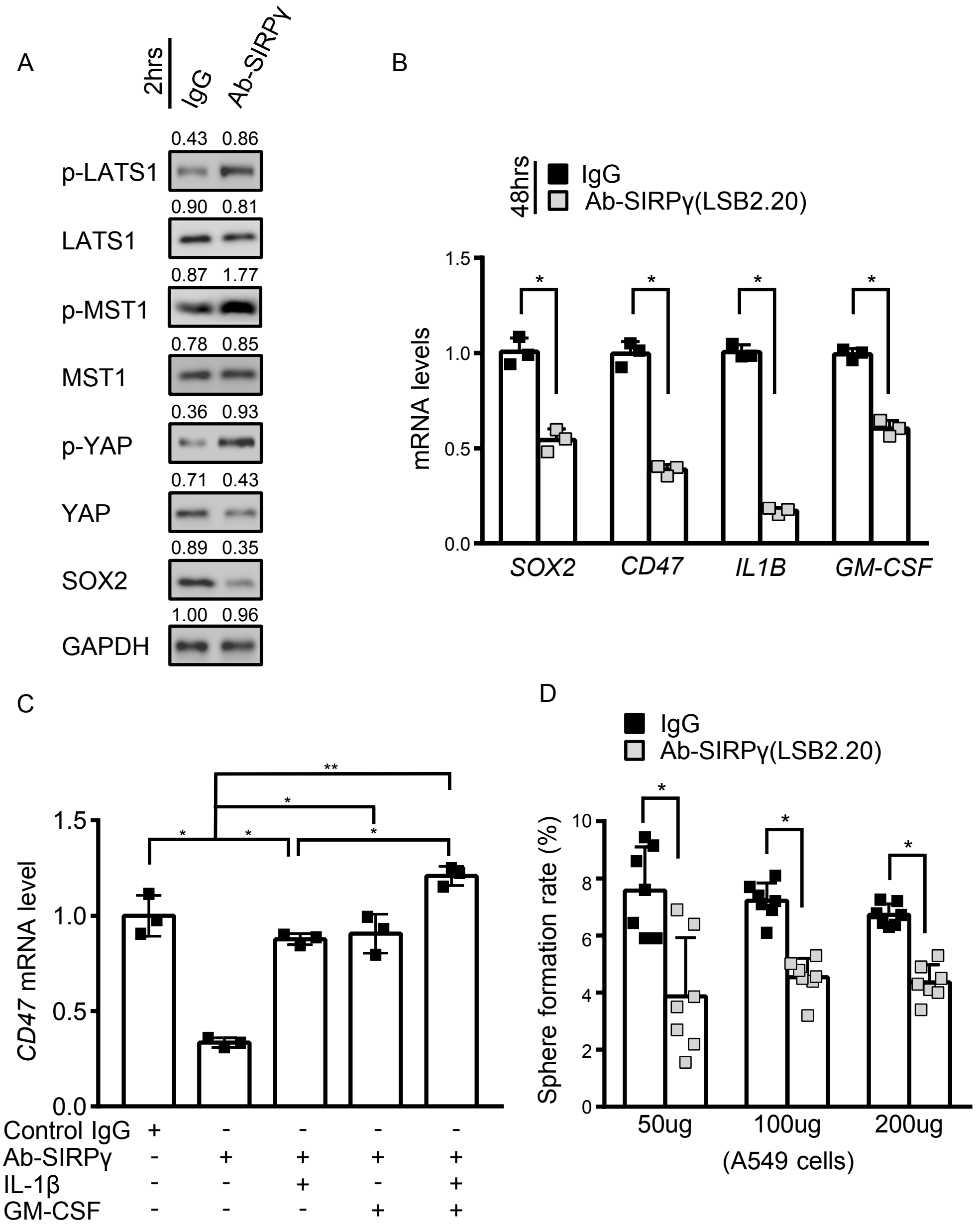

Supplemental Figure 14

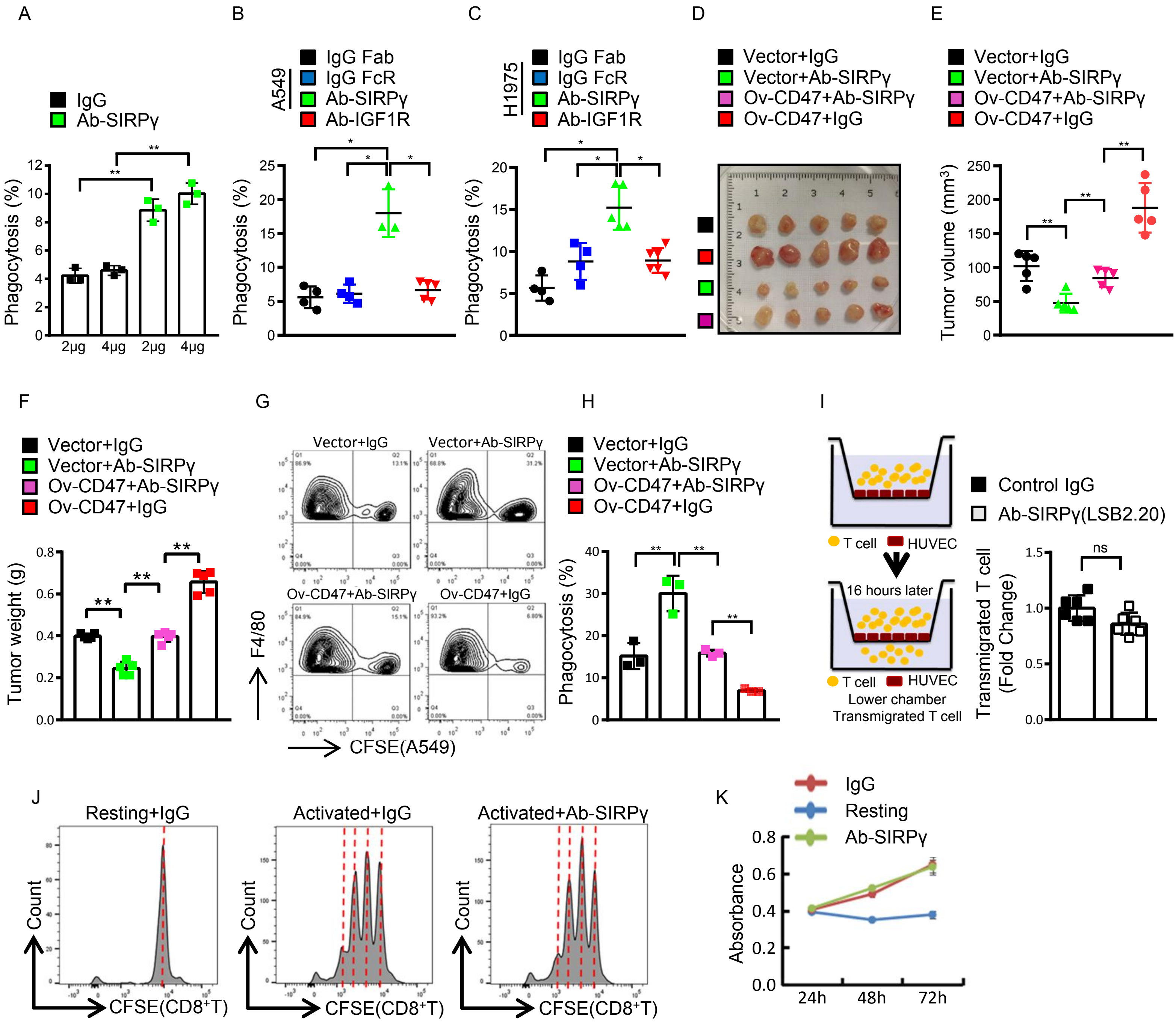

Supplemental Figure 15

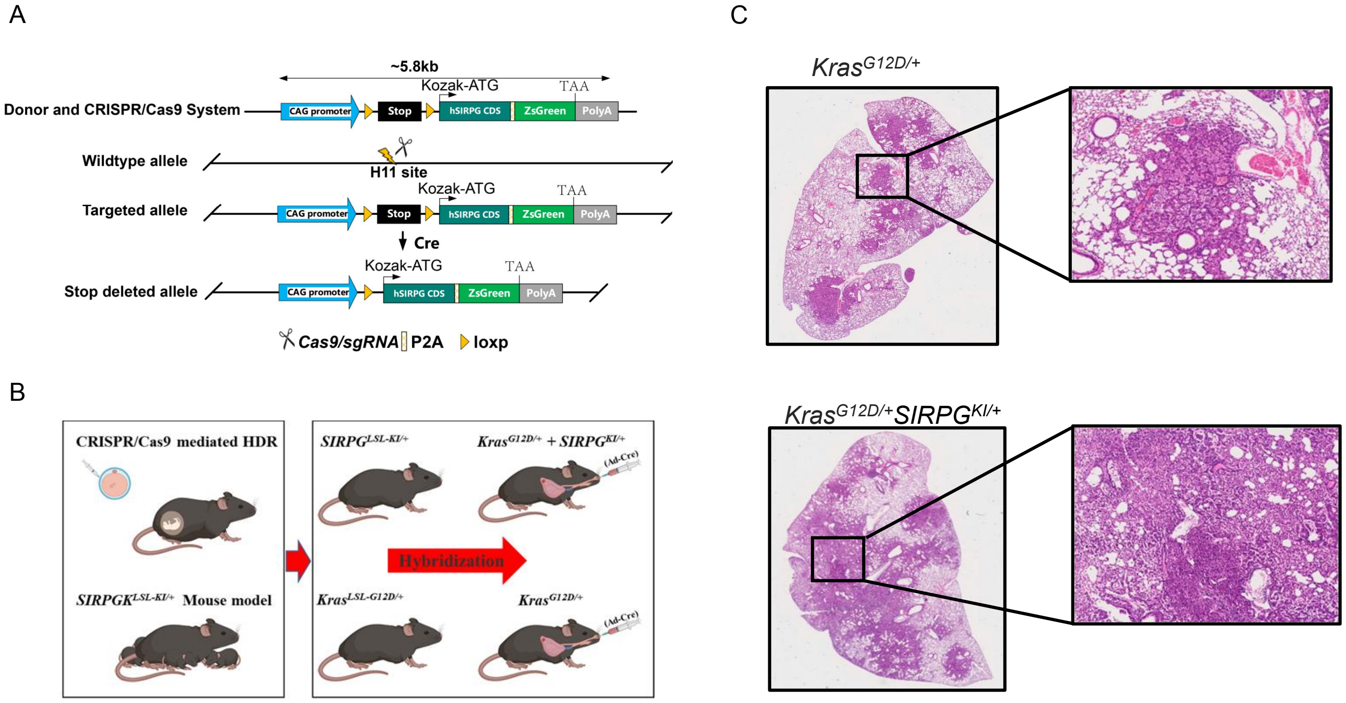

Supplemental Figure 16

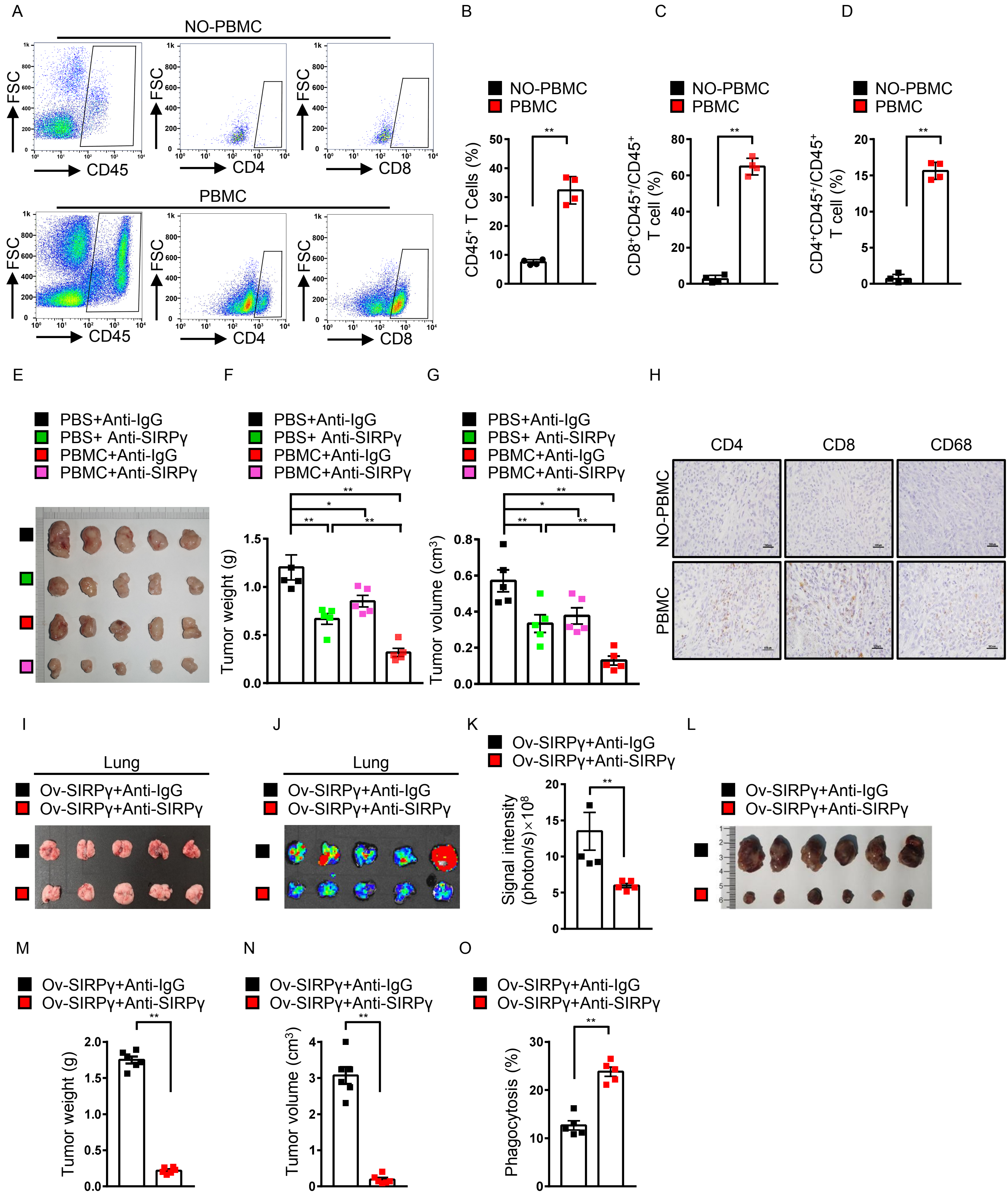

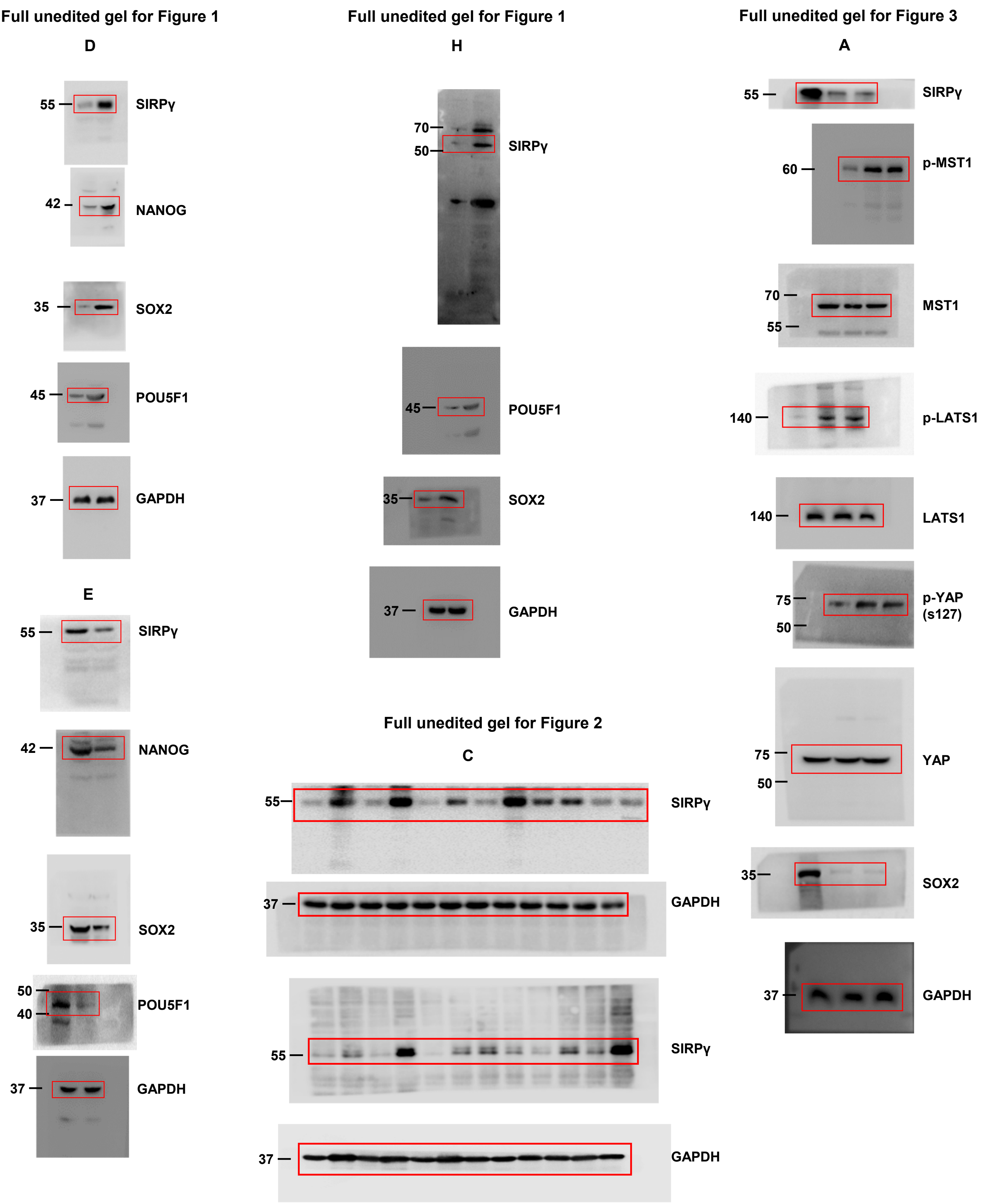

Supplemental Figure 17 (Page 1 of 9).

Presented are full, unedited, uncropped blots in order of appearance. Red boxes indicate the lanes presented in the corresponding figures.

Full unedited gel for Figure 3
B

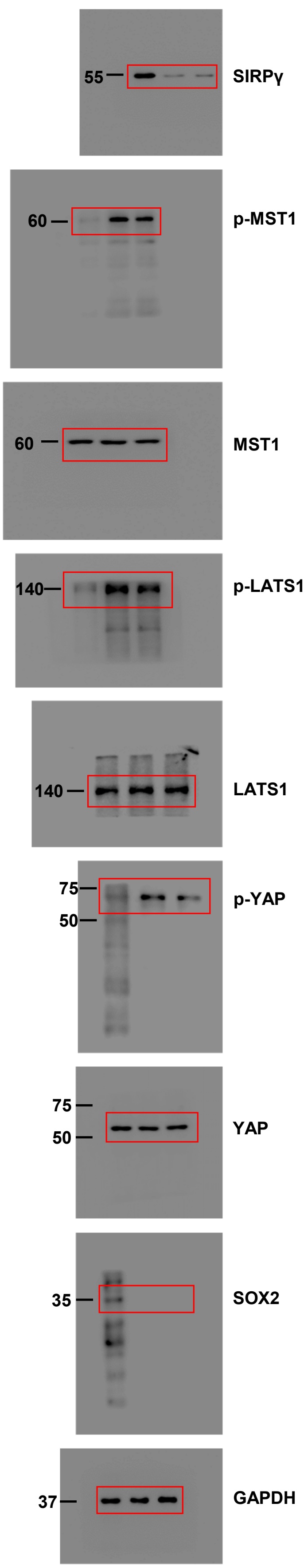

Full unedited gel for Figure 3
C

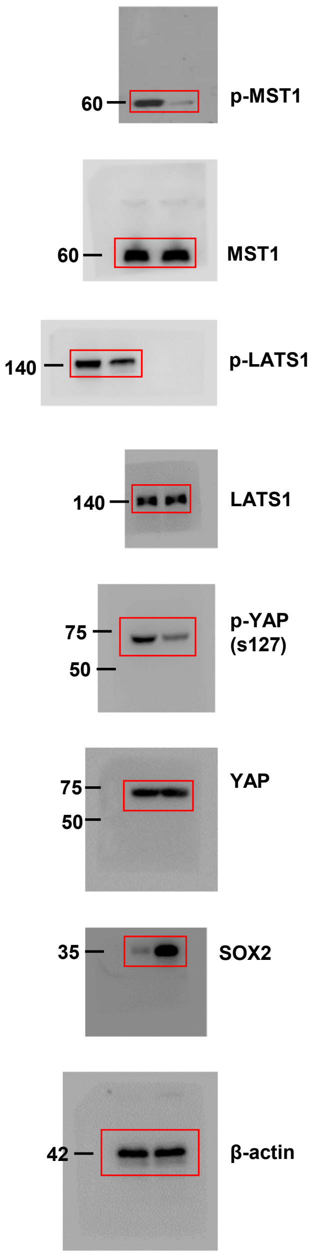

Full unedited gel for Figure 3
D

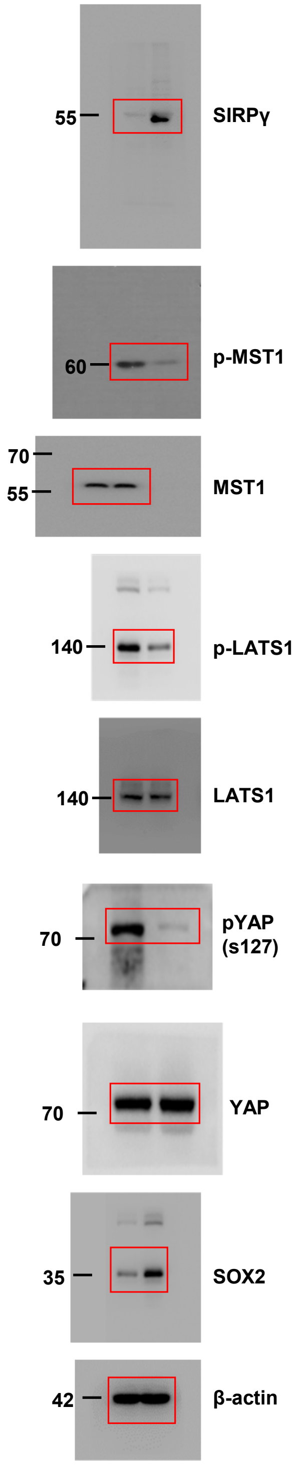

Full unedited gel for Figure 3
E

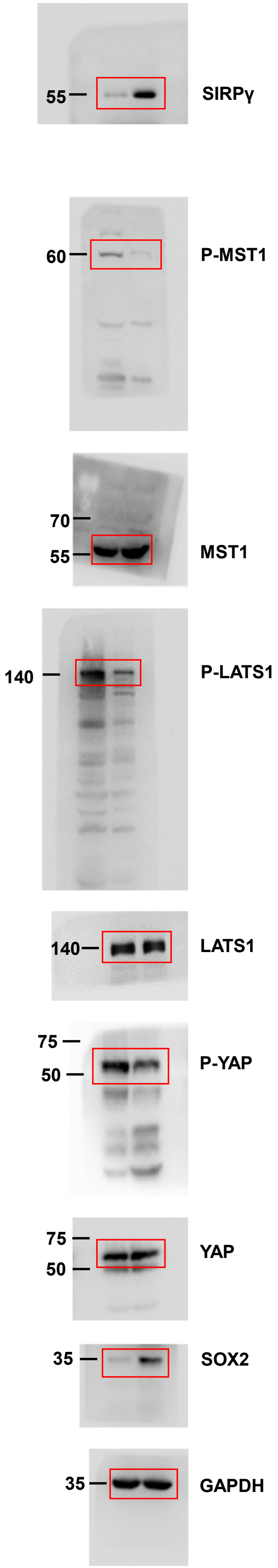

Supplemental Figure 17 (Page 2 of 9).

Presented are full, unedited, uncropped blots in order of appearance. Red boxes indicate the lanes presented in the corresponding figures.

Full unedited gel for Figure 3
H

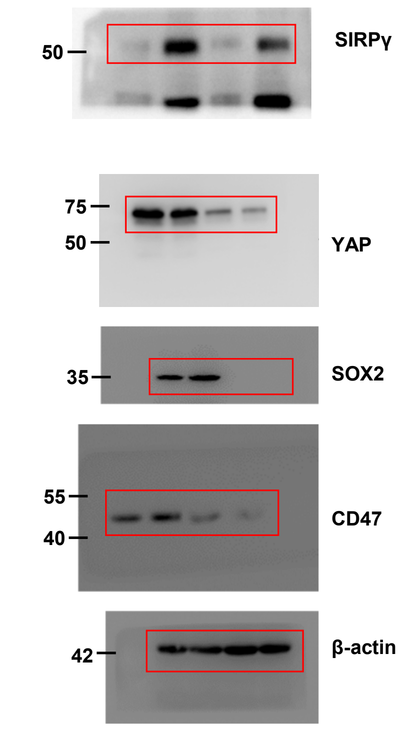

I

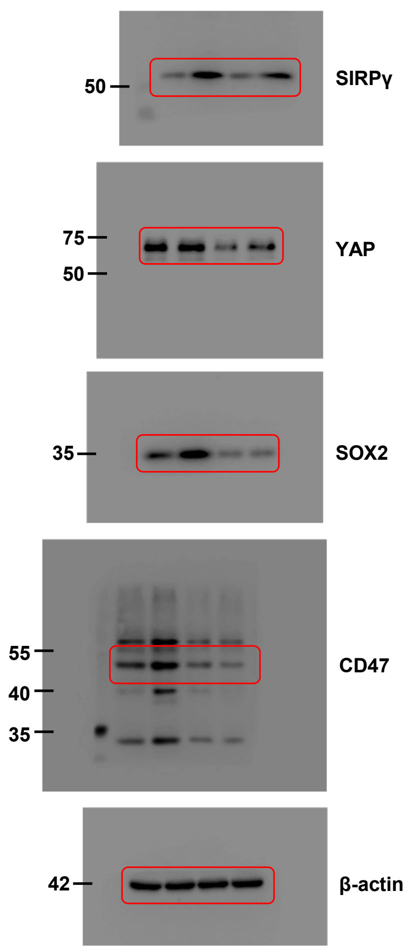

Full unedited gel for Figure 4
A

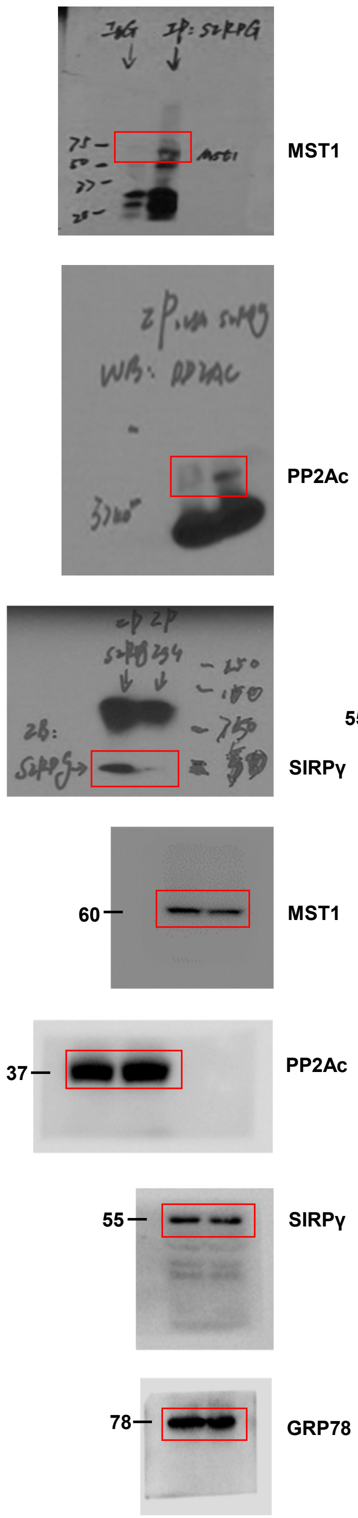

Full unedited gel for Figure 4
B

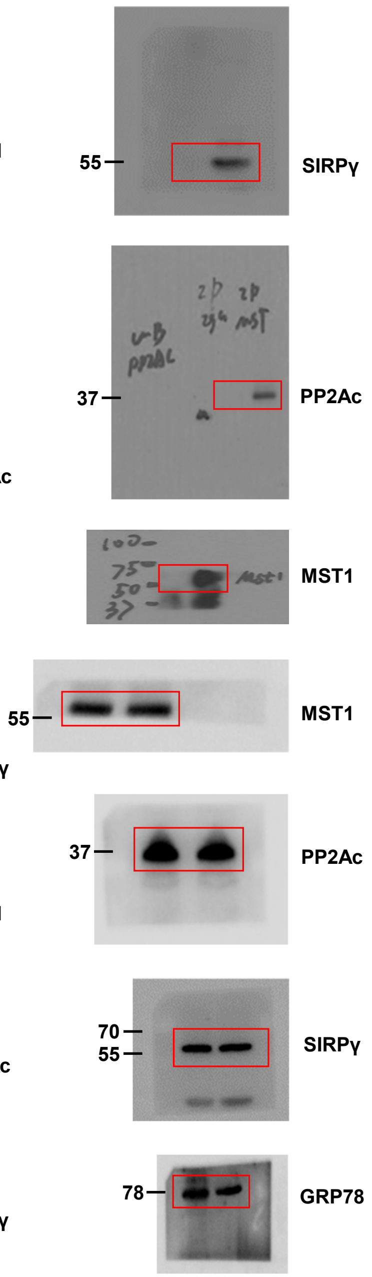

Full unedited gel for Figure 4
C

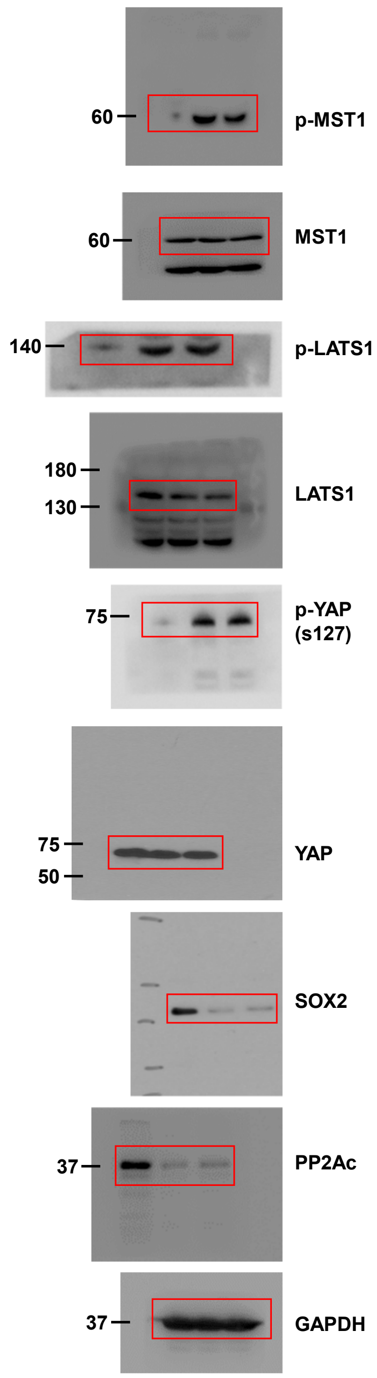

Supplemental Figure 17 (Page 3 of 9).

Presented are full, unedited, uncropped blots in order of appearance. Red boxes indicate the lanes presented in the corresponding figures.

Full unedited gel for Figure 4
D

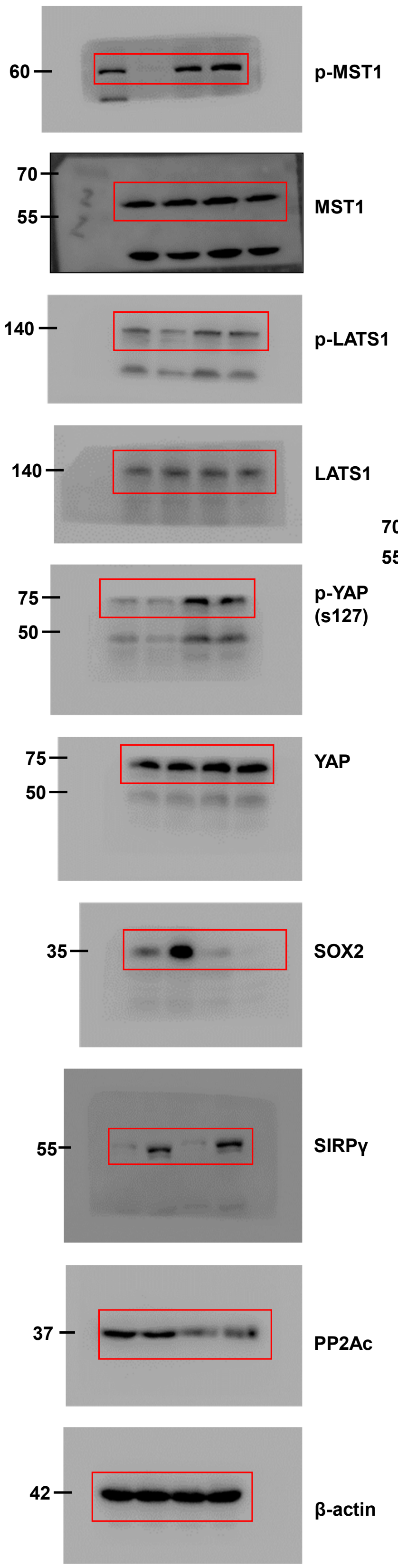

Full unedited gel for Figure 4
E

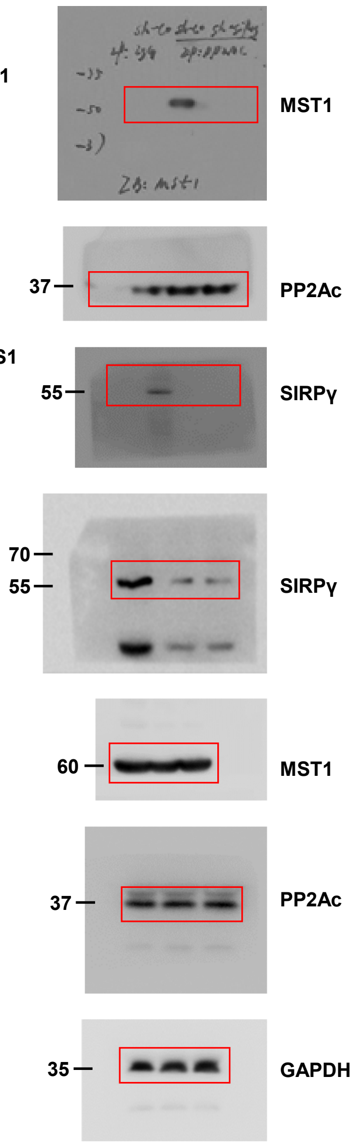

Full unedited gel for Figure 4
F

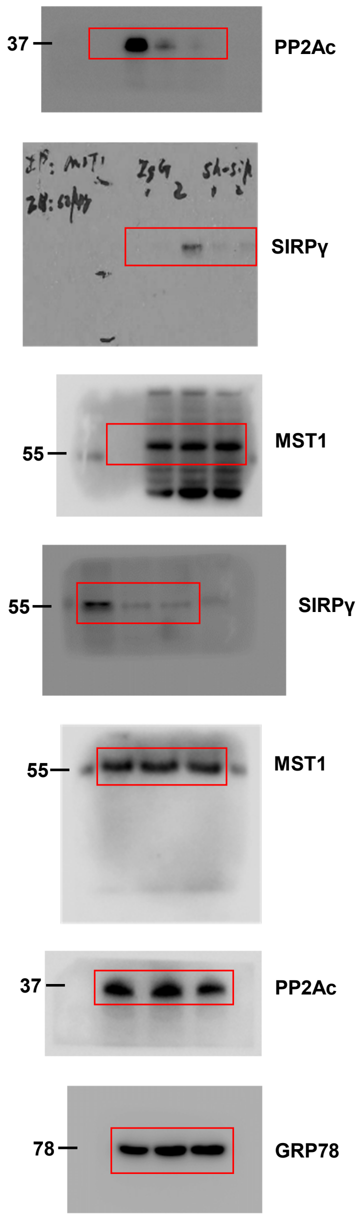

Full unedited gel for Figure 4
G

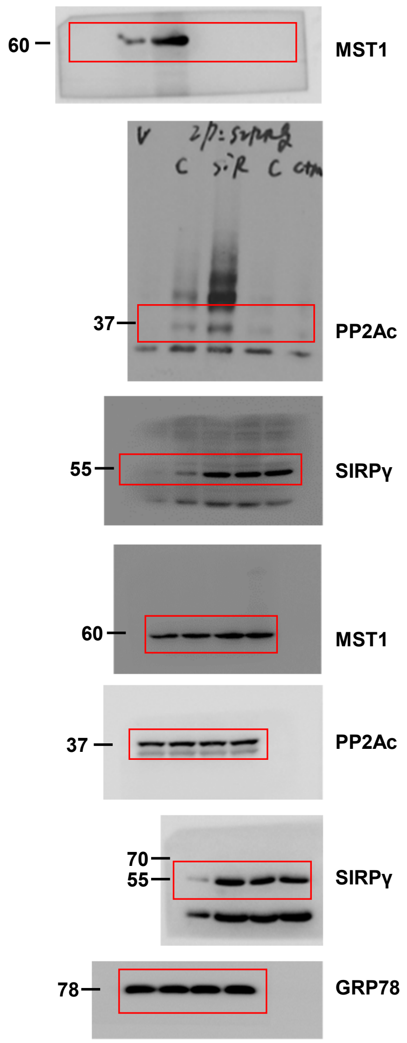

Full uncropt gel fot Figure 5

B

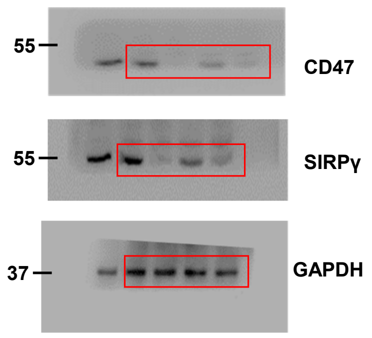

G

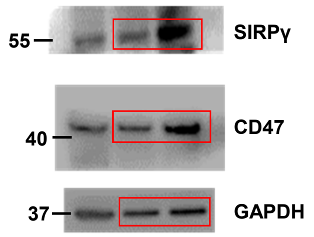

Supplemental Figure 17 (Page 4 of 9).
Presented are full, unedited, uncropped blots in order of appearance. Red boxes indicate the lanes presented in the corresponding figures.

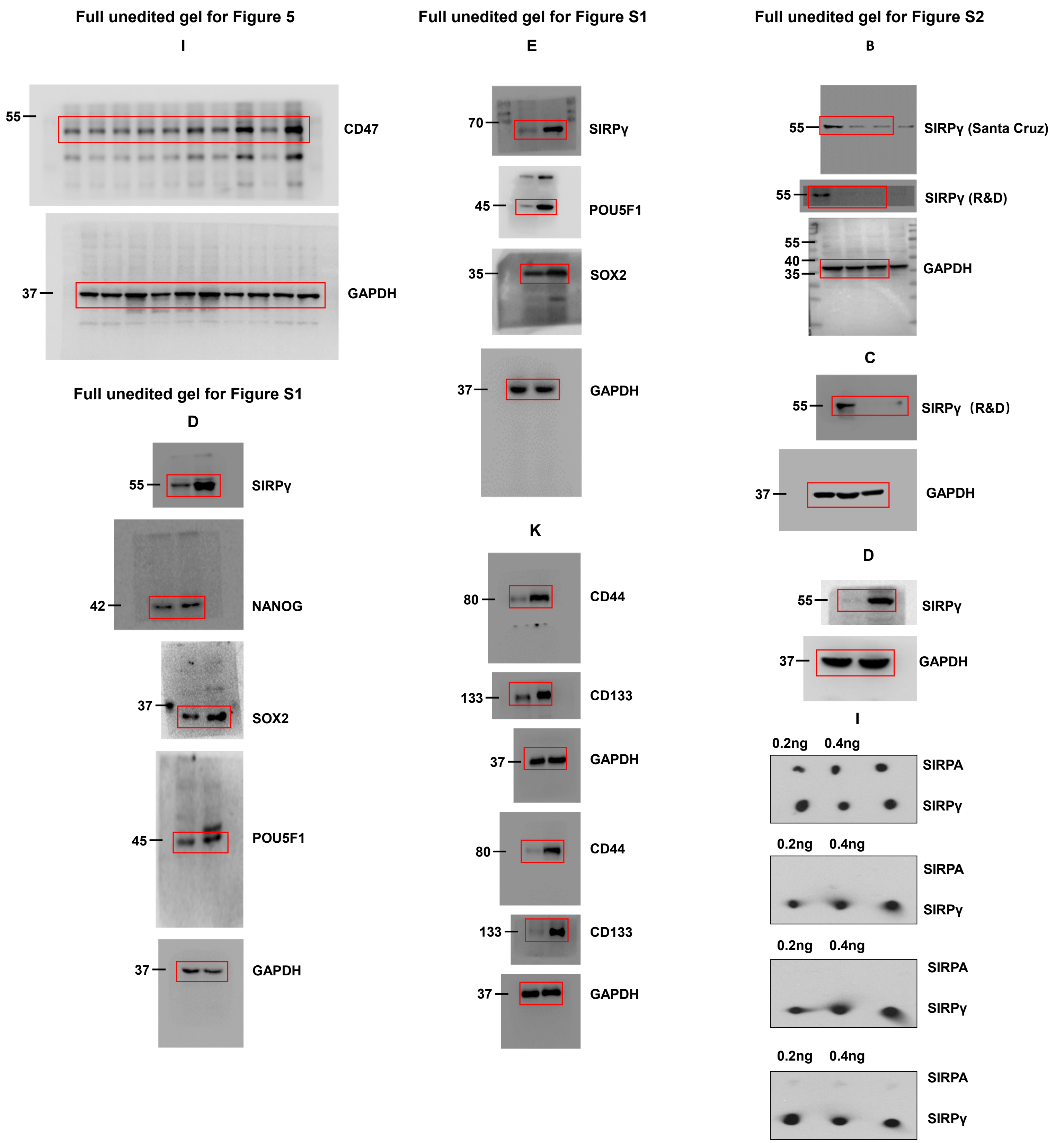

Supplemental Figure 17 (Page 5 of 9).
Presented are full, unedited, uncropped blots in order of appearance. Red boxes indicate the lanes presented in the corresponding figures.

Full unedited gel for Figure S2

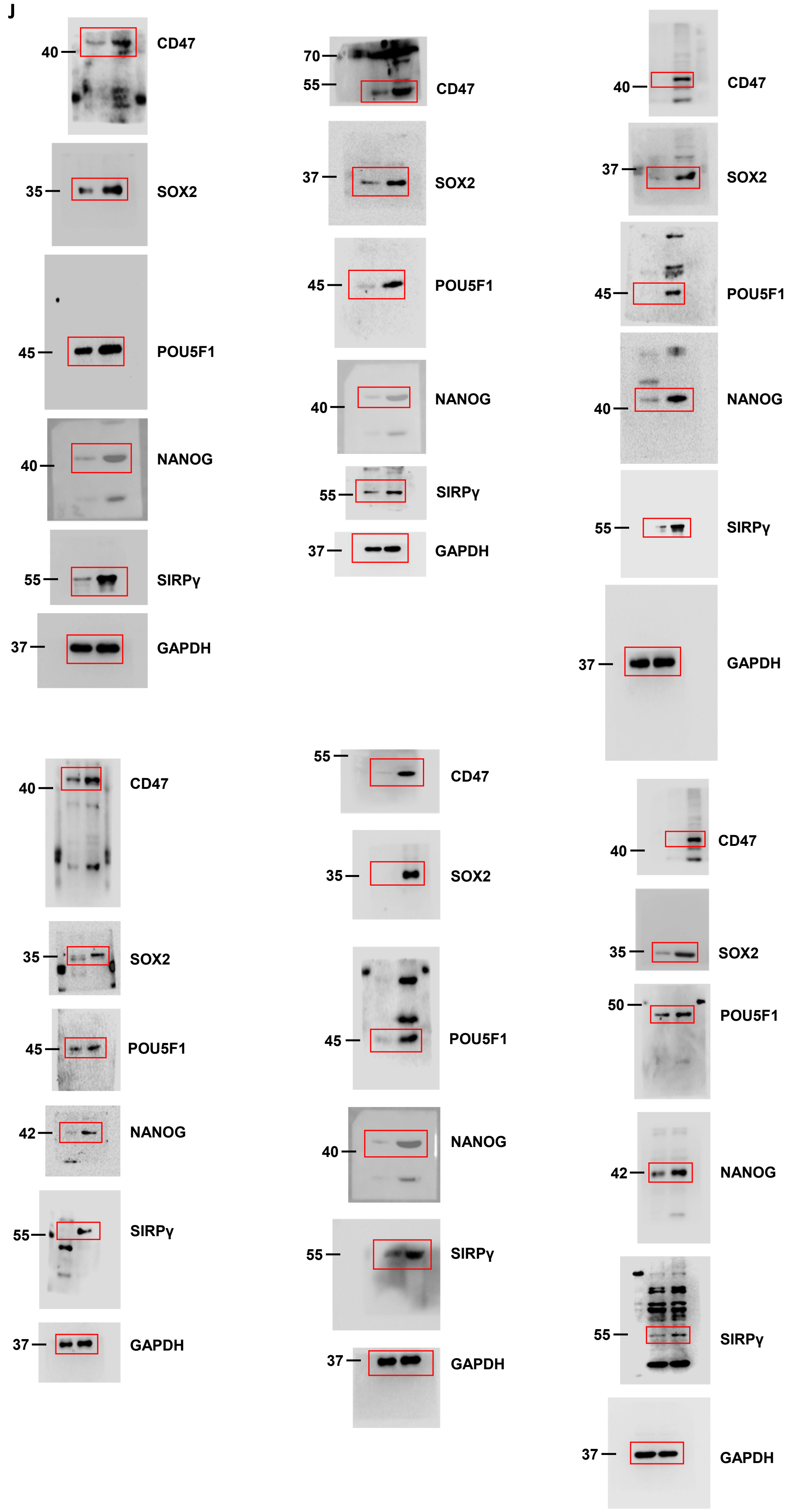

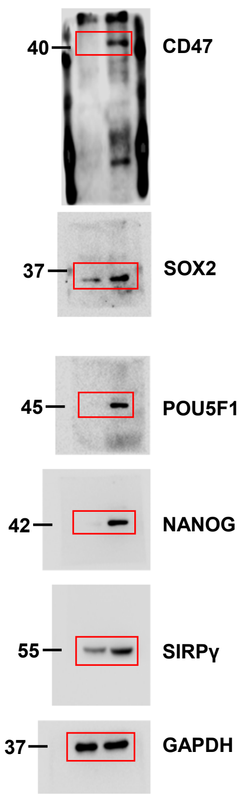

Supplemental Figure 17 (Page 6 of 9).

Presented are full, unedited, uncropped blots in order of appearance. Red boxes indicate the lanes presented in the corresponding figures.

Full unedited gel for Figure S3 B

Full unedited gel for Figure S3 C

Full unedited gel for Figure S3 D

Full unedited gel for Figure S3 E

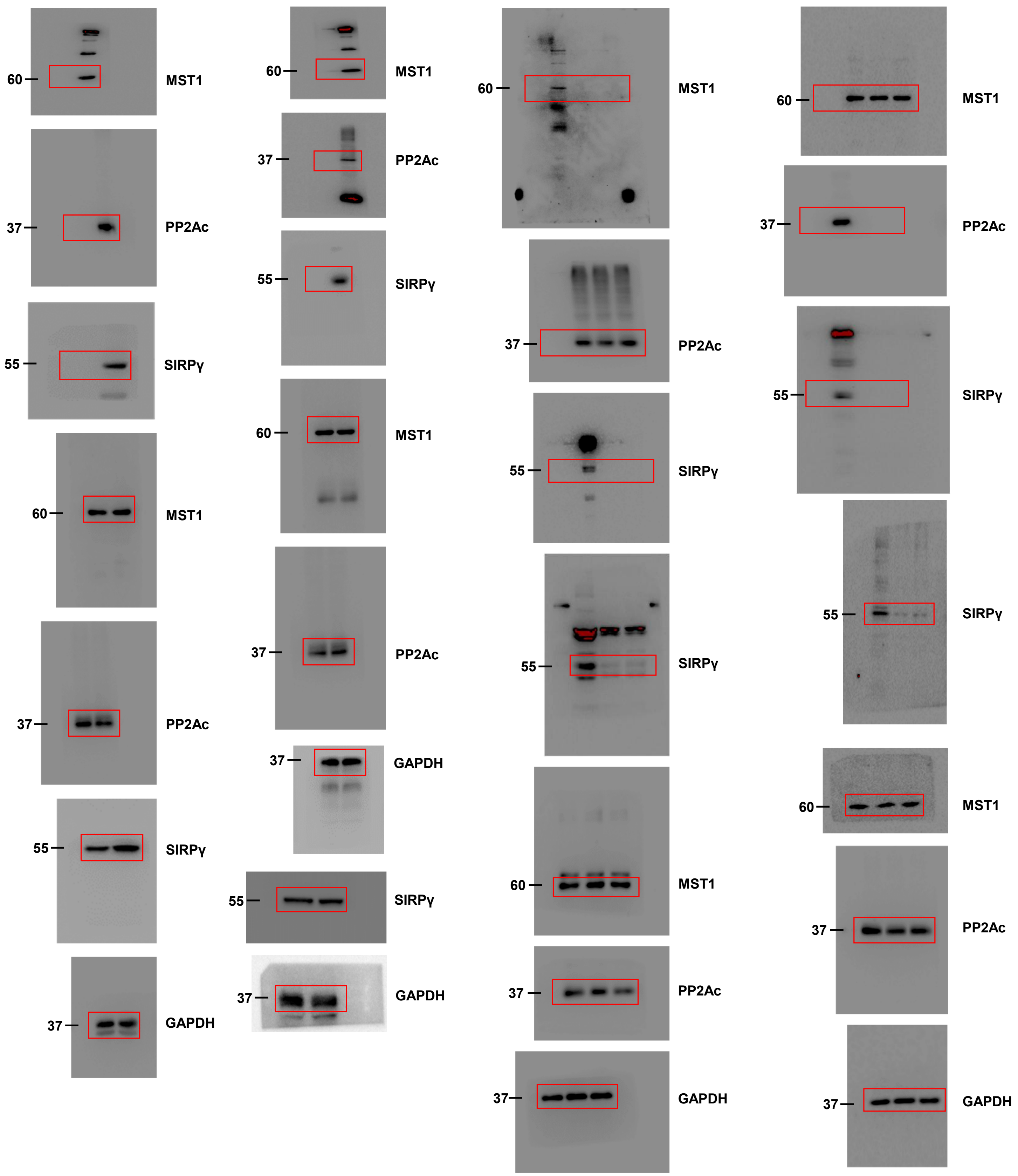

Supplemental Figure 17 (Page 7 of 9).

Presented are full, unedited, uncropped blots in order of appearance. Red boxes indicate the lanes presented in the corresponding figures.

Full unedited gel for Figure S3

F

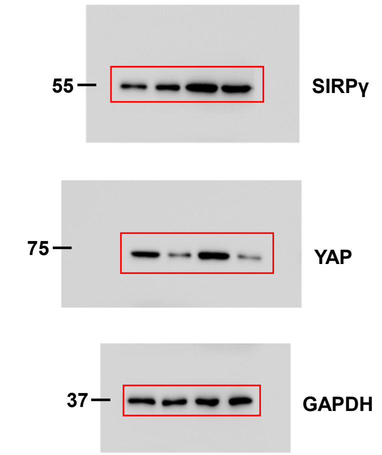

G

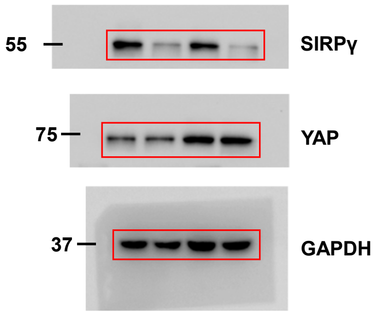

Full unedited gel for Figure S4

A

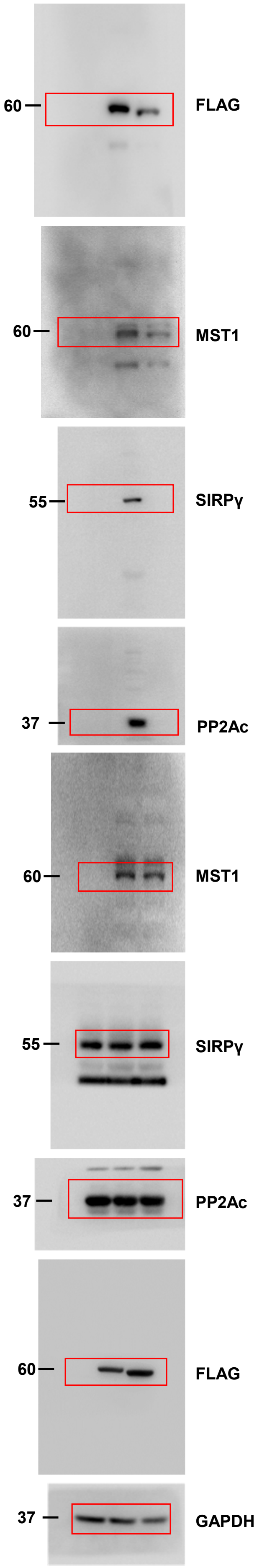

Full unedited gel for Figure S4

B

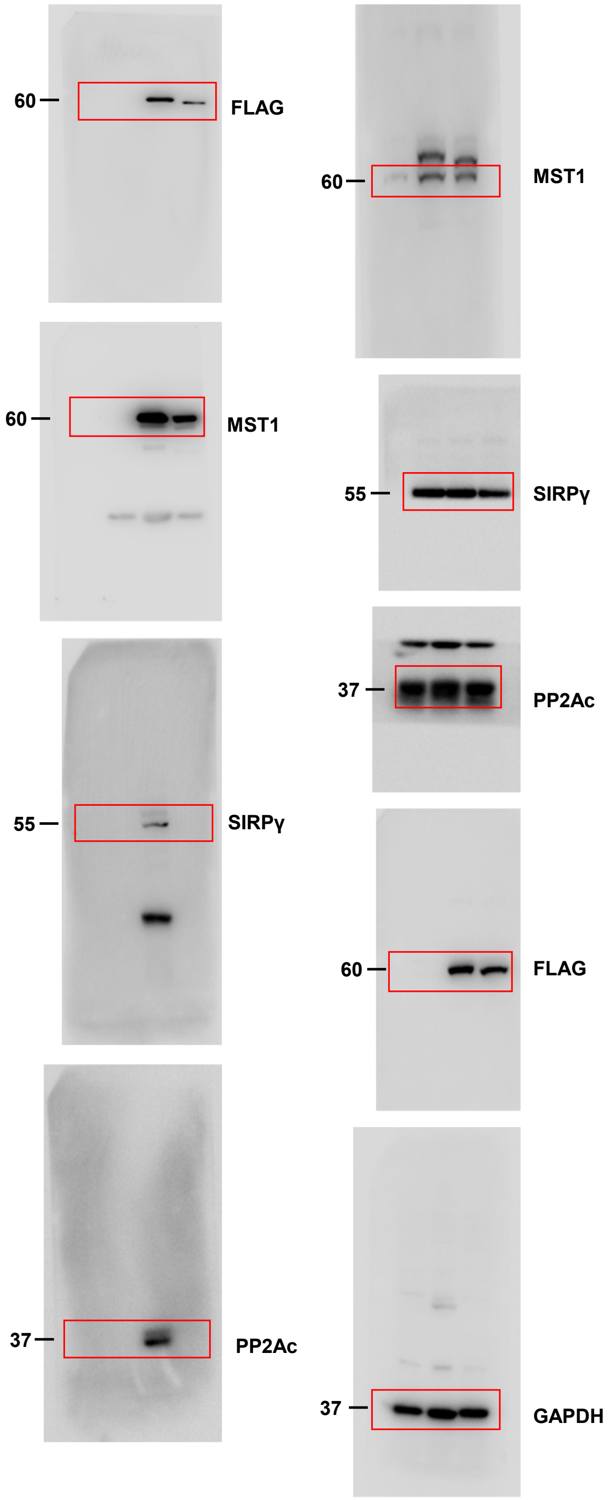

Supplemental Figure 17 (Page 8 of 9). Presented are full, unedited, uncropped blots in order of appearance. Red boxes indicate the lanes presented in the corresponding figures.

Full unedited gel for Figure S5

D

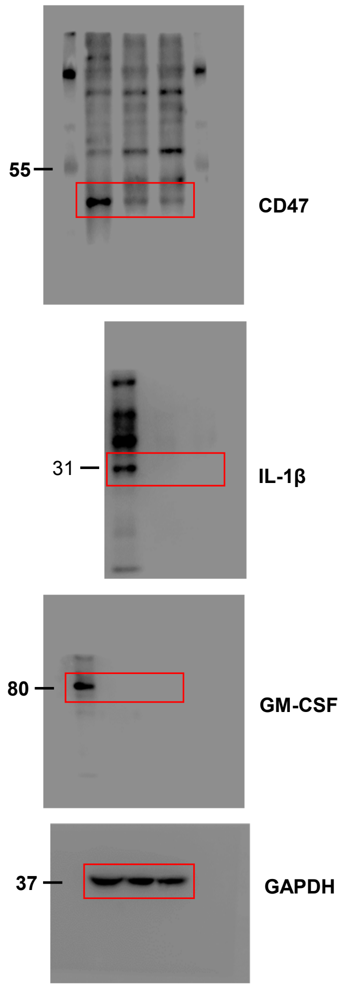

Full unedited gel for Figure S5

E

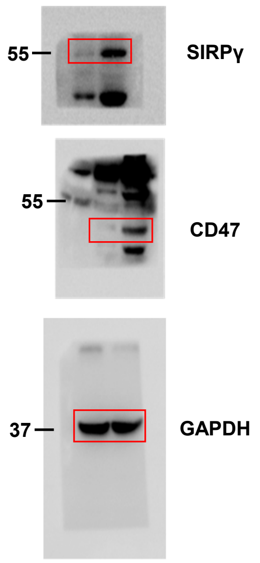

F

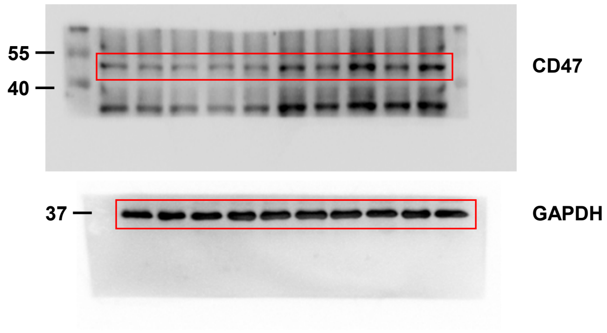

Full unedited gel for Figure S13

A

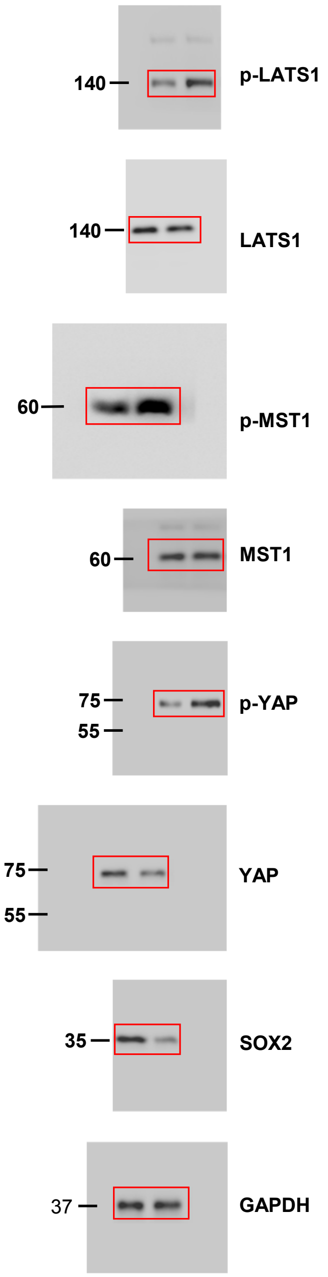

Supplemental Figure 17 (Page 9 of 9). Presented are full, unedited, uncropped blots in order of appearance. Red boxes indicate the lanes presented in the corresponding figures.
